# Cyclic nucleotide phosphodiesterases as drug targets

**DOI:** 10.1016/j.pharmr.2025.100042

**Published:** 2025-01-22

**Authors:** Michy P. Kelly, Viacheslav O. Nikolaev, Leila Gobejishvili, Claire Lugnier, Christian Hesslinger, Peter Nickolaus, David A. Kass, Walma Pereira de Vasconcelos, Rodolphe Fischmeister, Stefan Brocke, Paul M. Epstein, Gary A. Piazza, Adam B. Keeton, Gang Zhou, Mohammad Abdel-Halim, Ashraf H. Abadi, George S. Baillie, Mark A. Giembycz, Graeme Bolger, Gretchen Snyder, Kjetil Tasken, Nathaniel E.B. Saidu, Martina Schmidt, Manuela Zaccolo, Ralph T. Schermuly, Hengming Ke, Rick H. Cote, Soroush Mohammadi Jouabadi, Anton J.M. Roks

**Affiliations:** 1 Department of Neurobiology, Center for Research on Aging, University of Maryland School of Medicine, Baltimore, Maryland; 2 Institute of Experimental Cardiovascular Research, University Medical Center Hamburg-Eppendorf, Hamburg, Germany; 3 Department of Physiology, School of Medicine, University of Louisville, Kentucky, Louisville; 4 Translational CardioVascular Medicine, CRBS, UR 3074, Strasbourg, France; 5 Boehringer Ingelheim Pharma GmbH & Co. KG, Biberach, Germany; 6 Division of Cardiology, Department of Medicine, Johns Hopkins University School of Medicine, Baltimore, Maryland; 7 Department of Pharmacology and Molecular Sciences, Johns Hopkins University School of Medicine, Baltimore, Maryland; 8 Université Paris-Saclay, Inserm, Signaling and Cardiovascular Pathophysiology, UMR-S 1180, Orsay, France; 9 Department of Immunology, UConn Health, Farmington, Connecticut; 10 Department of Cell Biology, UConn Health, Farmington, Connecticut; 11 Department of Drug Discovery and Development, Harrison College of Pharmacy, Auburn University, Auburn, Alabama; 12 Georgia Cancer Center, Augusta University, Augusta, Georgia; 13 Department of Pharmaceutical Chemistry, Faculty of Pharmacy and Biotechnology, German University in Cairo, Cairo, Egypt; 14 School of Cardiovascular and Metabolic Health, University of Glasgow, Glasgow, UK; 15 Department of Physiology and Pharmacology, Cumming School of Medicine, University of Calgary, Calgary, Alberta, Canada; 16 BZI Pharma LLC, Beverly, Massachusetts; 17 Molecular Neuropharmacology, Intra-Cellular Therapies Inc (ITI), New York, New York; 18 Department of Cancer Immunology, Institute for Cancer Research, Oslo University Hospital, Oslo, Norway; 19 Institute of Clinical Medicine, University of Oslo, Oslo, Norway; 20 Department of Molecular Pharmacology, University of Groningen, Groningen, The Netherlands; 21 Groningen Research Institute for Asthma and COPD, GRIAC, University Medical Center Groningen, University of Groningen, Groningen, The Netherlands; 22 Department of Physiology, Anatomy and Genetics and National Institute for Health and Care Research Oxford Biomedical Research Centre, University of Oxford, Oxford, United Kingdom; 23 Department of internal Medicine, Justus Liebig University of Giessen, Giessen, Germany; 24 Department of Biochemistry and Biophysics, The University of North Carolina, Chapel Hill, North Carolina; 25 Department of Molecular, Cellular, and Biomedical Sciences, University of New Hampshire, Durham, New Hampshire; 26 Section of Vascular and Metabolic Disease, Department of Internal Medicine, Erasmus MC University Medical Center, Erasmus University Rotterdam, Rotterdam, The Netherlands

## Abstract

Cyclic nucleotides are synthesized by adenylyl and/or guanylyl cyclase, and downstream of this synthesis, the cyclic nucleotide phosphodiesterase families (PDEs) specifically hydrolyze cyclic nucleotides*.* PDEs control cyclic adenosine-3’,5’monophosphate (cAMP) and cyclic guanosine-3’,5’-monophosphate (cGMP) intracellular levels by mediating their quick return to the basal steady state levels. This often takes place in subcellular nanodomains. Thus, PDEs govern short-term protein phosphorylation, long-term protein expression, and even epigenetic mechanisms by modulating cyclic nucleotide levels. Consequently, their involvement in both health and disease is extensively investigated. PDE inhibition has emerged as a promising clinical intervention method, with ongoing developments aiming to enhance its efficacy and applicability. In this comprehensive review, we extensively look into the intricate landscape of PDEs biochemistry, exploring their diverse roles in various tissues. Furthermore, we outline the underlying mechanisms of PDEs in different pathophysiological conditions. Additionally, we review the application of PDE inhibition in related diseases, shedding light on current advancements and future prospects for clinical intervention.

**Significance Statement:**

Regulating PDEs is a critical checkpoint for numerous (patho)physiological conditions. However, despite the development of several PDE inhibitors aimed at controlling overactivated PDEs, their applicability in clinical settings poses challenges. In this context, our focus is on pharmacodynamics and the structure activity of PDEs, aiming to illustrate how selectivity and efficacy can be optimized. Additionally, this review points to current preclinical and clinical evidence that depicts various optimization efforts and indications.

## I Chapter 1: General introduction

Downstream of transmembrane receptors, intracellular signaling plays a major role in governing normal and pathological cell responses. The intracellular second messenger cyclic nucleotide cascades, driven by cyclic adenosine-3’,5’monophosphate (cAMP) and cyclic guanosine-3’,5’-monophosphate (cGMP), are conserved among species and ubiquitously expressed throughout mammalian tissues. Their involvement in many disease processes has led to enzymes within these cascades, being considered important drug targets. Sutherland (1972), Nobel laureate in 1971, showed how cAMP is a second messenger (Sutherland and Rall, 1960), is inactivated by phosphodiesterases (PDEs) through hydrolysis of 5’-adenosine monophosphate (5’-AMP; Butcher and Sutherland, 1962). Ashman et al (1963) showed the same for cGMP.

**Fig. 1:**
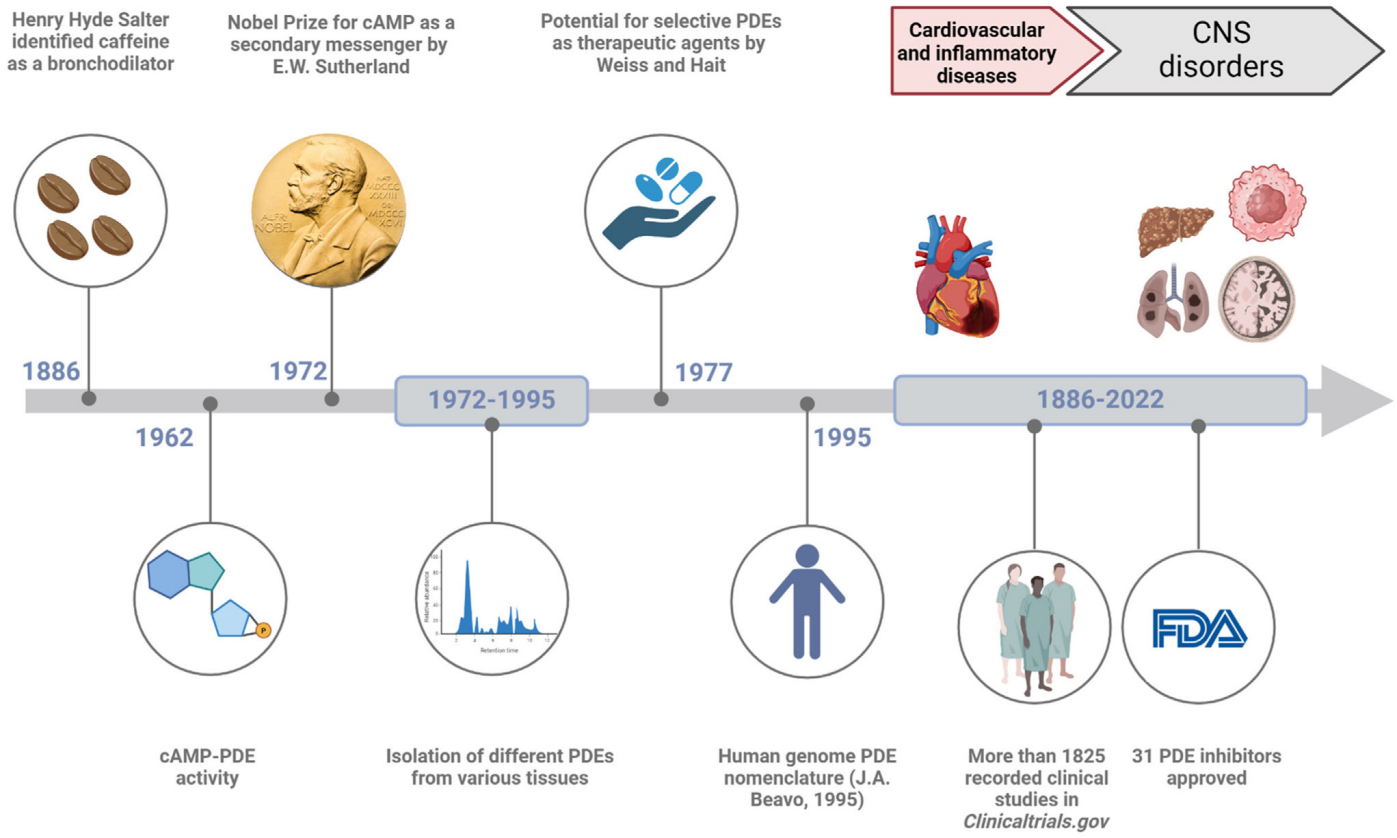
Timeline of PDEs in medicinal history. Henry Hyde Salter's discovery of caffeine's bronchodilator effects contributed to the exploration of PDEs, though the effect of caffeine is more likely to occur through adenosine receptors. In the mid-20th century, the discovery of PDEs as enzymes degrading cyclic nucleotides like cAMP and cGMP marked the beginning of their history. Early research by scientists such as Earl W. Sutherland Jr elucidated their roles in cellular signaling, flag stoned the way for drug development. These inhibitors have since evolved to treat conditions like pulmonary arterial hypertension and erectile dysfunction. Over time, research continued, uncovering new isoforms and therapeutic possibilities, extending the application of PDE inhibitors beyond cardiovascular diseases to conditions like cognitive dysfunction and fibrosis. Created with BioRender.com.

PDEs act in the presence of H_2_O and Mg^2+^ and hydrolyze the phosphate bond present 3’ in both cAMP and cGMP, producing H^+^ (Keravis et al, 2005). Thus, PDEs control the intracellular levels of cAMP (cAMP-PDE) and cGMP (cGMP-PDE) by rapidly catalyzing their inactivation and returning levels to the basal or resting state. PDEs as a result control short-term protein phosphorylation, long-term protein expression, and even epigenetic modifications (Abusnina et al, 2011). The role of cyclic nucleotides and PDEs in health and disease is widely investigated and has a long history (Fig. 1). In this article, we review the role of PDEs in various tissues (section General Introduction), explore the utility of PDE inhibitors in the treatment of various diseases (section Physiology and Clinical Development), and discuss considerations for future clinical applications (section General Perspectives and Future Directions).

### A Overview

Cyclic nucleotide PDEs are encoded by 11 gene families (PDE1 to PDE11), resulting in a large and complex number of proteins. Each family includes 1–4 distinct genes (a total of 21 in mammals) that produce more than 100 different proteins or isoenzymes [for reviews; see Conti and Beavo (2007), Keravis and Lugnier (2012), Azevedo et al (2014), Maurice et al (2014), and Ahmad et al (2015)]. PDEs were classified as a superfamily of metalophosphohydrolases and assigned the number EC 3.1.4.17.

**Fig. 2:**
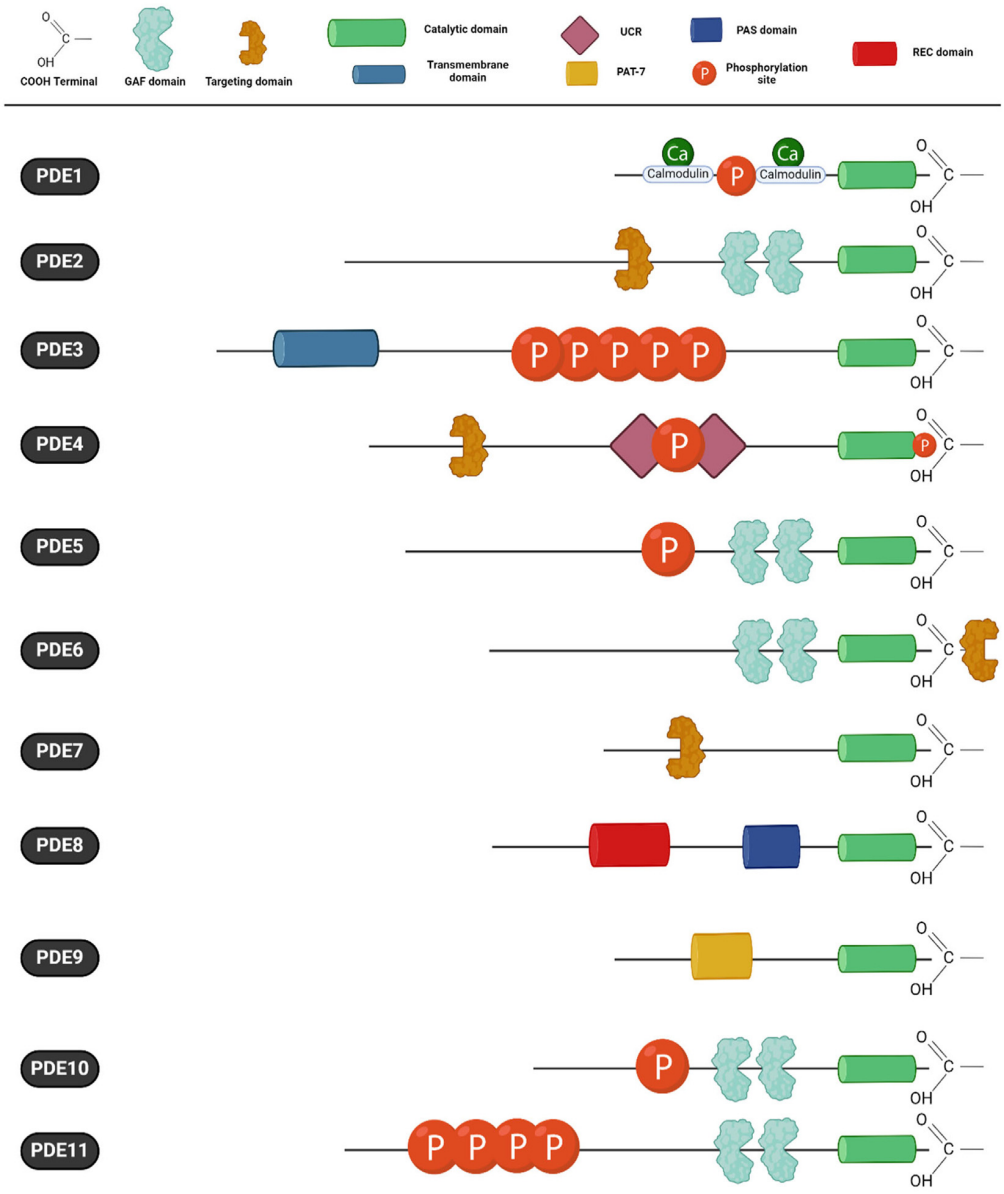
Phosphodiesterase (PDE) superfamily structure. PDEs are enzymes categorized into cAMP-specific, cGMP-specific, and dual-substrate families based on their affinity for cyclic nucleotides. Each of the 11 families possesses a catalytic domain at the COOH terminal. These isoforms also exhibit specific structural domains such as REC (Signal regulatory domain), PAS (PerARNT-Sim), UCR (upstream conversed region), PAT-7 (7-residue nuclear localization signal), and GAF (cGMP-binding ubiquitous motif), which play roles in regulating enzymatic activity, sensing cellular signals, influencing localization, and binding to cyclic nucleotides. lines indicate the length of the amino acid sequence of a representative member of each family. Adapted from Baillie et al (2019). Created with BioRender.com.

PDEs generally exist as dimers. Each monomer of the dimer has a common structure composed of 3 distinct domains: (1) the *N*-terminal regulatory domain, which characterizes each family and its variants; (2) the catalytic domain, which consists of about 340 amino acids and is relatively conserved among PDEs (∼78% amino acid identity); and (3) the *C*-terminal domain, which can be prenylated or phosphorylated (Anant et al, 1992; Dousa, 1999; Qin et al, 1992; Fig. 2).

The multiplicity of biochemical and structural properties of PDEs contributes to their tissue specificities, as well as cellular and subcellular distributions. The possibility of designing (relatively) disease-specific pharmacotherapy is not only an attractive feature of PDE as a drug target but also a challenge for pharmaceutical chemists (Fig. 1).

### B Family organization and evolutionary perspective

In the early years of PDE research (1980–1995), before an official nomenclature was established, PDEs were isolated from various tissues by chromatography. Their biochemical characteristics were determined according to the substrate hydrolyzed and how they were regulated (Thompson and Appleman, 1971; Keravis et al, 1980, Keravis et al, 2005), and PDEs were named accordingly, eg, cGMP-stimulated PDE (cGS-PDE) and cAMP-PDE, cGMP-inhibited PDE (cGI-PDE), (Lugnier and Schini, 1990); CaM-activated PDE (Lugnier et al, 1986); rolipram-inhibited PDE (ROI-PDE; Komas et al, 1989), etc. To avoid confusion, an official nomenclature system based on the human genome was developed in 1995 (Beavo, 1995), creating a unique descriptor for each PDE. For example, PDE4D7 indicates a 3’,5’-cyclic nucleotide PDE of the PDE4 gene family, gene D, splice variant 7. We now discuss the PDEs accordingly.

#### 1 PDE1 family

PDE1, initially named CaM-PDE, represents the sole family that is Ca^2+^-dependently regulated via CaM (a 16 kDa Ca^2+^-binding protein complexed with 4 Ca^2+^ ions). The PDE1 isoenzyme family is encoded by 3 genes: *PDE1A* (mapped on human chromosome 2q32), *PDE1B* (human chromosome location (hcl): 12q13), and *PDE1C* (hcl: 7p14.3). More than 10 human isoforms have been identified with molecular weights that vary from 58 to 86 kDa per monomer. The *N*-terminal regulatory domain contains 2 Ca^2+^/CaM-binding domains and 2 phosphorylation sites that modulate the biochemical activity of these enzymes (Fig. 2). PDE1A and PDE1B preferentially hydrolyze cGMP, whereas PDE1C hydrolyzes cAMP and cGMP with similar *K*_m_ values. Phosphorylation of PDE1A1 (59 kDa) and PDE1A2 (61 kDa) by PKA, and phosphorylation of PDE1B1 by CaM kinase II, decreases their sensitivity to Ca^2+^ and CaM and, thereby, reduces PDE1 activity (Zhao et al, 1997). PDE1 isoenzymes are mainly cytosolic, although PDE1A has been found in the nucleus where it contributes to the regulation of transcription factor activity and epigenetic control of gene transcription (Abusnina et al, 2011). Very few selective PDE1 inhibitors are available. To be effective, PDE1 inhibitors must inhibit both basal- and CaM-activated activity. Nimodipine was the first compound with this property, acting in the micromolar range (Epstein et al, 1982; Keravis and Lugnier, 2012). Dioclein followed, showing to relax the human saphenous vein (Goncalves et al, 2009). Nowadays, IC86340 (PDE1A/PDE1C) and lenrispodun (ITI-214) are available as selective inhibitors with submicromolar potency (Maurice et al, 2014; Wennogle et al, 2017).

#### 2 PDE2 family

The PDE2 family, formerly cGS-PDE (Martins et al, 1982; Yamamoto et al, 1983), consists of a single gene (hcl: 11q 13.4) that produces 3 splice variants of dual cAMP and cGMP hydrolyzing enzymes: cytosolic PDE2A1 (Sonnenburg et al, 1991) and membrane-bound PDE2A2 and PDE2A3 (Rosman et al, 1997). The *N*-termini direct these isoenzymes to their different subcellular locations. The *N*-terminal domain has 2 cGMP-binding domains, GAF-A and GAF-B (Fig. 2). The GAF-A domain mediates PDE2 dimerization. GAF-B binds cGMP allosterically (1–5 *μ*M), positively stimulating cAMP hydrolysis up to 30-fold with *K*_m_ values from 10 to 30 *μ*M (Lugnier and Schini, 1990). EHNA (IC_50_ = 2 *μ*M) was the first PDE2 inhibitor (Duncan et al, 1982; Méry et al, 1995; Podzuweit et al, 1995; Masood et al, 2009). Later, Bay 60-7550 (IC_50_ = 4.7 nM) was discovered, effective only against cGMP-activated PDE2 (Boess et al, 2004; Masood et al, 2009). PDE2 inhibitor ND7001 was patented (WO2004041258-A2) and inhibits both basal- and cGMP-stimulated PDE2 (Masood et al, 2009), putatively the most effective tool to inspect the role of PDE2 in cellular signaling (Lueptow et al, 2016).

#### 3 PDE3 family

The PDE3 family, formerly cGI-PDE, is encoded by 2 genes: *PDE3A* (hcl: 12p12) and *PDE3B* (hcl: 11p15.1). Three variants are expressed for PDE3A: *PDE3A1* (136 kDa), *PDE3A2* (118 kDa), and *PDE3A3* (94 kDa), whereas only a single *PDE3B1* (137 kDa) variant has been identified, despite PDE3B of various sizes having been reported. PDE3 hydrolyses both cAMP and cGMP, and a unique 44-amino acid insert in the catalytic domain is present, which is different between PDE3A and PDE3B (Ahmad et al, 2015; Fig. 2). The *V*_max_ for cAMP hydrolysis is 10-fold higher than for cGMP hydrolysis. cGMP has a higher affinity for PDE3 than cAMP and is a competitive inhibitor of cAMP hydrolysis (Lugnier, 2006). Accordingly, PDE3 participates in cAMP/cGMP cross-talk (Lugnier et al, 1999a). The PDE3 variants possess *N*-terminal hydrophobic membrane association regions allowing the targeting of PDE3 to various specific intracellular domains (Keravis and Lugnier, 2012). PDE3 is located in the cardiac sarcoplasmic reticulum (SR; Lugnier et al, 1993), cardiac nuclear envelope, near nucleopore complexes (Lugnier et al, 1999b), and in liver Golgi endosomal fraction (Geoffroy et al, 2001). A PKA phosphorylation site is present in PDE3A1 and PDE3A2 for activation, acting as negative feedback for cAMP signaling, whereas an Akt/protein kinase B (PKB) phosphorylation site is only present on PDE3A1 (Wechsler et al, 2002) to promote 14-3-3-protein binding and inhibit phosphatase-catalyzed inactivation (Palmer et al, 2007). PKB-dependent phosphorylation also activates PDE3B. PDE3A3 lacks PKA and PKB phosphorylation sites. PKC phosphorylates and activates PDE3A (Pozuelo Rubio et al, 2005).

The first potent and selective inhibitor described of PDE3 was cilostamide (Hidaka et al, 1979) and showed potential for application in cardiac disease. This led to the development of amrinone, milrinone, and enoximone. Milrinone (IC_50_ = 2.1 *μ*M) was the first PDE3 inhibitor developed to improve myocardial contraction in heart failure and safe for short-term use, avoiding death by arrhythmia after chronic use (Lanfear et al, 2009). Another PDE3 inhibitor, cilostazol (IC_50_ = 0.2 *μ*M) has been approved by the United States Food and Drug Administration (FDA) for the treatment of intermittent claudication and is marketed as *Pletal* (Real et al, 2018).

#### 4 PDE4 family

The cAMP-selective PDE4 family is arguably the most studied family. The more than 25 human isoforms (50–125 kDA; Paes et al, 2021b) are encoded by 4 genes: *PDE4A* (hcl: 19p13.2), *PDE4B* (hcl: 1p31), *PDE4C* (hcl: 19p13.1), and *PDE4D* (hcl: 5p12). These genes feature different promoters and are subject to alternative mRNA splicing. Although the expression of all predicted splice variants of the PDE superfamily in general is a matter of debate, there is evidence that many of the PDE4 isoforms are expressed as functional proteins in various human tissues, including the brain, heart, and immune cells, supporting their role as genuine protein products rather than hypothetical precursors, possibly unlike other PDE subtypes (Bolger, 1994; Conti, 2000; Houslay and Adams, 2003; Baillie, 2009).

PDE4 variants contain 2 unique stretches of amino acids called upstream conserved region (UCR) 1 and UCR2 (Beard et al, 2000; Fig. 2). The so-called “long” PDE4 isoforms contain both UCR1 and UCR2, whereas the “short” isoforms are *N*-terminally truncated and contain only UCR2, and even shorter (“super-short”) isoforms lack either a portion or the entirety of the UCR2 element (Paes et al, 2021b; Kyurkchieva and Baillie, 2023). Allosteric PDE4 activity regulation is detailed in section Allosteric Mechanisms for Regulating Cyclic Nucleotide Hydrolysis by PDEs. The catalytic region contains an extracellular signal-regulated kinase (ERK) phosphorylation site for the activation of PDE4 short forms and inhibition of PDE4 long forms (Baillie et al, 2000; Houslay, 2001). Long-form PDE4 variants containing UCR1 and UCR2 can form dimers, which involve the UCRs (Bolger et al, 1993; Bolger et al, 2015). PDE4 variants are localized to specific subcellular nanodomains by A-kinase anchoring proteins (AKAP; Dodge et al, 2001). PDE4s interact directly with many other intracellular proteins, thereby defining the PDE4 interactome.

Rolipram (IC_50_ = 5 μM) and Ro 20-1724 (IC_50_ = 18 μM) were the first inhibitors shown to be highly selective for PDE4 (Lugnier et al, 1986; Reeves et al, 1987). Thereafter, pharmaceutical companies synthesized many PDE4 inhibitors, particularly, as anti-inflammatory agents (Houslay et al, 2005; Warren et al, 2023). Some of these inhibitors have been approved for medicinal use and are described in the section Physiology and Clinical Development.

#### 5 PDE5 family

PDE5 specifically hydrolyses cGMP and is encoded by 1 gene *PDE5A* (hcl: 4q27) with 3 variants being expressed: PDE5A1 (100 kDa), PDE5A2 (95 kDa), and PDE5A3 (95 kDa). Their *N*-termini contain tandem GAF-A and GAF-B domains (Fig. 2). Only the GAF-A domain binds cGMP (Turko et al, 1998), promoting protein kinase G (PKG)-catalyzed PDE5 phosphorylation (Nakamura et al, 2018). This prompts catalytic activity and increases cGMP-binding affinity converting PDE5 (see section PDE Regulation of Cardiac Function Through cGMP for further details). Zaprinast (M&B 22948) was the first PDE5 inhibitor described (IC_50_ = 0.4 *μ*M; Lugnier et al, 1986), followed by sildenafil (*Viagra*), the first clinically used oral PDE5 inhibitor (IC_50_ = 4 nM; Boolell et al, 1996; Ballard et al, 1998). Zaprinast also inhibits PDE9 with an IC_50_ value of 35 *μ*M (Fisher et al, 1998), and sildenafil’s IC_50_ is only 10-fold to 80-fold lower for PDE5 than for PDE1 and PDE6, respectively. More selective PDE5 inhibitors are vardenafil, tadalafil, and avanafil (Andersson, 2018). However, tadalafil has an affinity for PDE11 that is about 20 times lower than for PDE5 (Weeks et al, 2009). Thus, there is still a need to develop more selective PDE5 inhibitors. The use of molecular fingerprint-based virtual screening protocols and structure-based pharmacophore development could facilitate the identification of more selective compounds (Kayik et al, 2017).

#### 6 PDE6 family

The cGMP-selective PDE6 family consists of 3 genes that encode catalytic subunits (PDE6A, PDE6B, and PDE6C) that are expressed at high concentrations in rod and cone photoreceptors in the retina [for a recent review, see Cote (2021)]. PDE6A (also referred to as the *α*-subunit) and PDE6B (*β*-subunit) are localized to rod photoreceptor cells, whereas PDE6C (*α*’-subunit) is expressed in cone photoreceptor cells. PDE6 catalytic subunits (and other components of the visual signaling pathway) have also been identified in the pineal gland (Carcamo et al, 1995). The PDE6 family is unique in several respects: (1) rod PDE6 is the only PDE that can form a heterodimer (*αβ*), (2) enzyme regulation is mediated by binding of regulatory *γ*-subunits (PDE6G (rod, *γ*) and PDE6H (cone, *γ*’) that inhibit the catalysis of cGMP in the nonactivated state, (3) activation of the PDE6 holoenzyme (*αβγγ* or *α*’*α*’*γ*’*γ*’) results upon binding of the photoreceptor G-protein *α*-subunit (GNAT1 in rods, GNAT2 in cones) and displacement of the *γ*-subunit from the enzyme active site, (4) activated PDE6 is the only PDE family that catalyzes cGMP hydrolysis at a diffusion-controlled rate, (5) the C-terminus of each catalytic subunit is prenylated, conferring tight association of PDE6 with the membrane, and (6) the expression and proper assembly of the PDE6 holoenzyme requires a photoreceptor-specific chaperone, aryl hydrocarbon receptor-interacting protein-like 1 (Yadav and Artemyev, 2017; Cote, 2021). It is well established that the rate-limiting step for the activation and deactivation of the visual signaling pathway in photoreceptors is controlled by the kinetics of PDE6 activation and inactivation, respectively (Pugh and Lamb, 2000; Arshavsky and Wensel, 2013). The rapid reduction in cGMP concentration in the photoreceptor cell upon PDE6 activation results in the closure of cyclic nucleotide-gated ion channels and the generation of an electrical response.

Similar to PDE5, PDE6 catalytic subunits consist of 2 tandem GAF domains attached to the catalytic domain, and cGMP binding to the GAF-A domain contributes to the allosteric regulation of the holoenzyme (Kameni Tcheudji et al, 2001; Zhang et al, 2008c). The molecular organization of the PDE6 holoenzyme has revealed that the inhibitory *γ*-subunits bind to the catalytic dimer in an extended conformation that interacts with each of the GAF and catalytic domains (Gulati et al, 2019; Irwin et al, 2019). The pharmacological properties of PDE6 are similar in many respects to PDE5 (as described in the previous section), with zaprinast being notable for having a 10-fold higher affinity for rod and cone PDE6 compared with PDE5 (Zhang et al, 2005c).

#### 7 PDE7 family

The PDE7 family specifically hydrolyses cAMP. There is no known regulatory domain in the *N*-terminal region (Fig. 2). This family includes 2 genes, *PDE7A* (hcl: 8q13) and *PDE7B* (hcl: 6q23-q24), with alternative splicing for *PDE7A* giving rise to PDE7A1 (57 kDa), PDE7A2 (50 kDa), and PDE7A3 (50 kDa). PDE7A3 lacks a part of the catalytic domain structure and retains the capacity of PDE7A1 to interact and inhibit the catalytic subunit of PKA. Four alternative splice *PDE7B* transcripts have been identified, of which PDE7B1 and PDE7B3 show 2 putative phosphorylation sites for PKA. Although PDE7B protein is expressed in various cell types, endogenous translation has not been confirmed for all predicted splice variants. Yet, no endogenous PDE7B proteins have been detected. IC242 was the first selective PDE7 inhibitor to be reported (IC_50_= 0.84 *μ*M; Lee et al, 2002), and several new PDE7 inhibitors have been listed since (Zorn and Baillie, 2023).

#### 8 PDE8 family

The PDE8 isoenzyme family specifically hydrolyses cAMP with the highest affinity among all PDEs. It is encoded by *PDE8A* (hcl: 15q25.3) and *PDE8B* (hcl: 5q13.3). The primary structure of PDE8 includes *N*-terminal response regulator receiver (REC), Per-Arnt-Sim (PAS), and 3 putative PKA and PKG phosphorylation sites (Fig. 2). Various splice variants exist. PDE8A1, the longest (93 kDa) and most frequently expressed variant, contains REC and PAS domains. PDE8A2 lacks the PAS domain, whereas PDE8A3 and the truncated PDE8A4 and PDE8A5 lack both REC and PAS domains (Wang et al, 2001). PDE8B1 and PDE8B4 contain both REC and PAS domains, whereas PDE8B2 and PDE8B3 have a deletion in the PAS domain (Gamanuma et al, 2003). I*κ*B proteins activate PDE8A1 through interaction with the PAS domain. Interestingly, PDE8A regulates the Raf-1 and ERK signaling networks (Ahmad et al, 2015). PF-04957325 is a selective PDE8 inhibitor with possible application in airway disease and autoimmune encephalomyelitis (see section The Role of PDEs in Specific Immune Cell Types; Johnstone et al, 2018; Basole et al, 2022).

#### 9 PDE9 family

The PDE9 family specifically hydrolyses cGMP with the highest affinity among all PDE families. Twenty-one *N*-terminal mRNA variants can be encoded by a single gene, *PDE9A* (hcl: 21q22.3), along with 3 PDE9A protein isoforms that are much larger than the predicted molecular weight of these mRNA variants [named by molecular weight: PDE9X-100, PDE9X-120, and PDE9X-175; Patel et al (2018)]. No regulatory function or phosphorylation of the *N*-terminal domain has been reported. PDE9A isoforms are differentially expressed and subcellularly localized in various tissues, and this changes with age (Wang et al, 2003b; Patel et al, 2018). PDE9A inhibitor BAY 73-6691 has 25-fold selectivity for the target over all other PDEs (Wunder et al, 2005). Orally available, brain-penetrant PDE9A inhibitors, BI-409306 and PF-04447943, have been developed for use in age-related cognitive decline (see section PDE Isoforms, Cognitive Impairment, and Alzheimer).

#### 10 PDE10 family

PDE10 represents a dual-substrate family of enzymes encoded by the gene, *PDE10A*. Human *PDE10A* maps to chromosome 6q26-27. There are 18 splice variants that can be derived from *PDE10A* (*PDE10A1 to PDE10A19*; MacMullen et al, 2016). The deduced amino acid sequence contains 779 amino acids (88 kDa), including 2 GAF domains in the *N*-terminal region (Fujishige et al, 2000; Fig. 2). In contrast to other PDEs, the GAF-A domain apparently binds only cAMP. Due to its kinetic properties for cAMP and cGMP hydrolysis, cGMP hydrolysis by PDE10 is potently inhibited by cAMP and thus opposite to PDE3. Papaverine (IC_50_= 36 nM; Tian et al, 2011) and PF-2545920 are PD10A inhibitors, and were reported to cause seizures through neuronal excitability enhancement (Zhang et al, 2017c). Various possible clinical applications are highlighted in the section Physiology and Clinical Development.

#### 11 PDE11 family

The dual-substrate PDE11 isoenzyme family has a catalytic site more similar to PDE5 than to PDE10A. Four *N*-terminal variants are encoded by the *PDE11A* gene, which maps to human chromosome 2 (2q31.2): PDE11A1 to PDE11A4. PDE11A1 (491 amino acids; predicted molecular mass 56 kDa) was cloned from human skeletal muscle and contains a partial GAF-B domain. PDE11A2 (65.8 kDa) and PDE11A3 (78 kDa) contain a complete GAF-B domain, with PDE11A3 also containing an incomplete GAF-A domain in the *N*-terminal region. PDE11A4 (100 kDa) is the longest protein within this family and includes 2 full GAF domains and multiple phosphorylation sites within the *N*-terminal region (Fawcett et al, 2000; Yuasa et al, 2000; Pilarzyk et al, 2022). The PDE11A4 GAF-A domain allosterically binds cGMP (Gross-Langenhoff et al, 2006). The degradation-resistant cGMP analog *Rp*-8pCPT-PET-cGMP binds the GAF-A domain to stimulate the catalytic activity of PDE11A4, but cGMP binding does not (Jager et al, 2012). The GAF-B domain is involved in oligomerization of the enzyme (Weeks et al, 2007). Reports of PDE11A expression at the protein level are highly contradictory, probably due to antibody nonspecificity and species differences (Kelly, 2015). Tissue and species differences in PDE11A isoforms have been abundantly reported (Kelly, 2015, 2018b; Pilarzyk et al, 2022; Sbornova et al, 2023). The first published selective PDE11A inhibitors were identified using a, which showed a moderate affinity for PDE11A (Ceyhan et al, 2012). With the help of yeast-based high-throughput assays that yielded inhibitors with IC_50_ of 0.11–0.33 *μ*M, the compounds BC11–28 and BC11–38 have been identified, showing highly selective inhibition (>350-fold selective for PDE11A vs other PDE families). Improvement of potency and pharmacokinetic properties based on these scaffolds is ongoing (Mahmood et al, 2023).

### C Structure-activity relationship

As reviewed in section Family Organization and Evolutionary Perspective, PDEs are divided into a variable regulatory domain at the *N*-terminus and a conserved catalytic domain at the *C*-terminus, and specifically recognize substrates and inhibitors (Conti and Beavo, 2007; Francis et al, 2011; Maurice et al, 2014; Baillie et al, 2019). In the present section, the structure-activity relationships and the exploitation thereof for the design of PDE subtype-selective inhibitors are reviewed.

#### 1 Regulation of catalytic domain

**Fig. 3:**
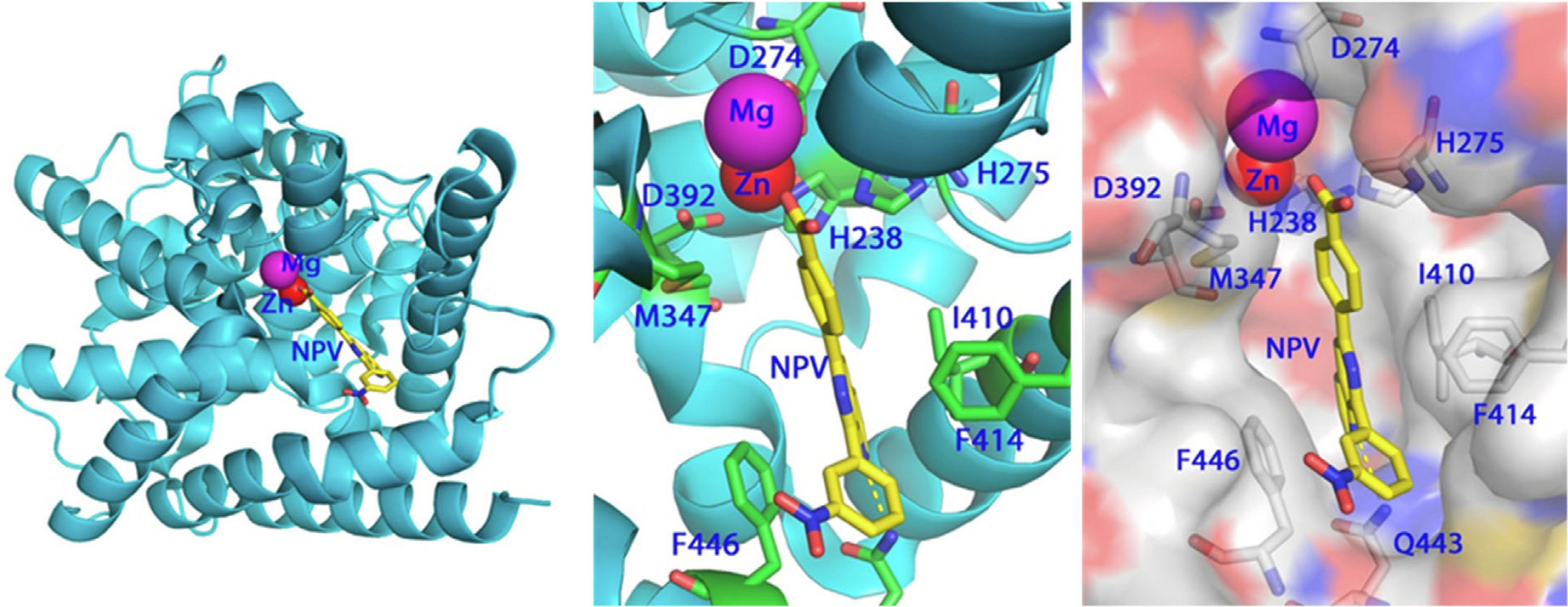
The catalytic domain of PDE4B2B in complex with NPV. This folding is conserved in the catalytic domains of all PDE families. The catalytic domain (Pfam: PF00233) typically consists of 16 *α*-helices that bind two divalent metals, Zn^2+^ and (typically) Mg^2+^ that are essential for the catalysis of cAMP and cGMP to their linear form. The 10-membered ring of NPV stacks against the benzyl ring of Phe446 on 1 side and interacts with hydrophobic residues of Ile410 and Phe414 on another side (Wang et al, 2007b).

**Fig. 4:**
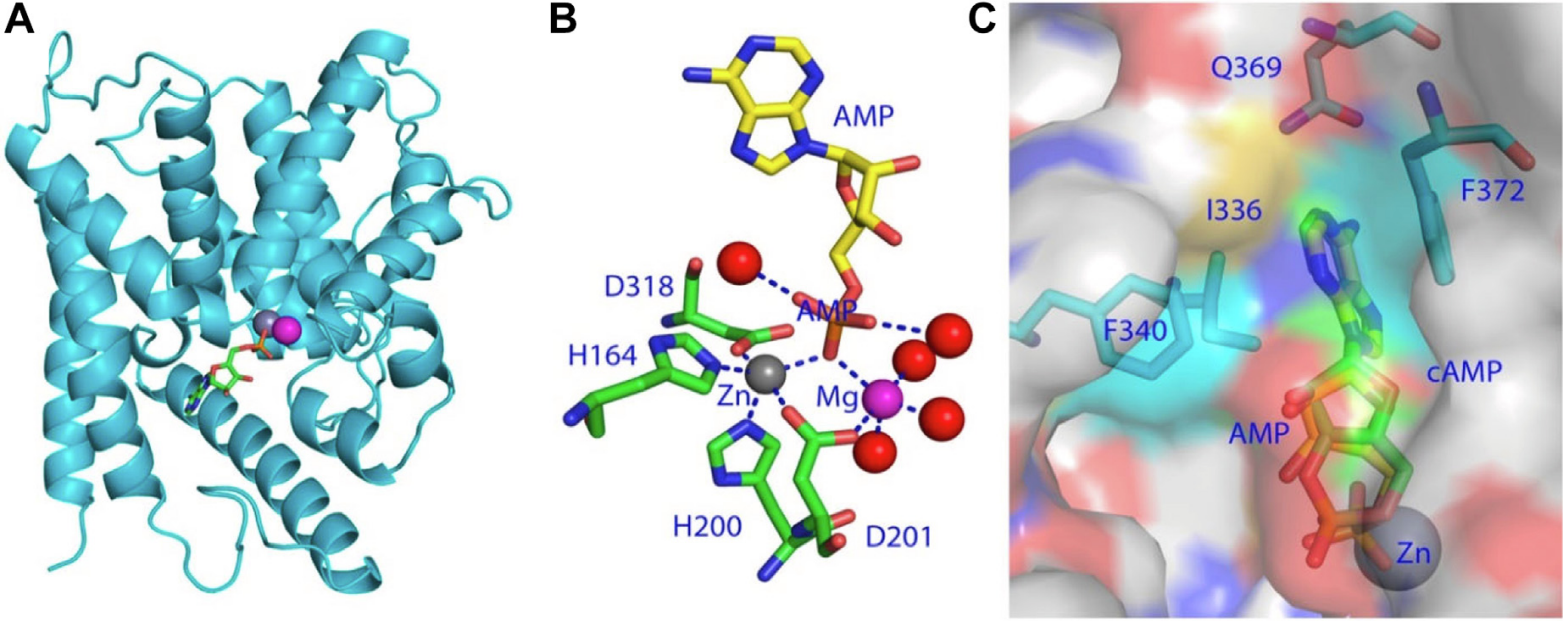
Structures of PDE4. (A) Ribbon model of catalytic domain of PDE4D in complex with 5′-AMP [PDB code of 1PTW; adapted from Huai et al (2003) and Wang et al (2008b)]. Gray and pink spheres are zinc and magnesium, respectively. Sticks represent 5′-AMP. (B). Interaction of metal ions with 5′-AMP. (C) Surface representation of the active site of D201N PDE4D mutant in complex with substrate cAMP [PDB code, 2PW3 (Wang et al, 2007b)]. Phe372 on 1 side of the purine ring and Phe230 and Ile336 on another side form the “hydrophobic clamp.” The invariant Gln369 forms a hydrogen bond with substrate cAMP.

The first atomic-level structure of the PDE4B2B catalytic domain (Xu et al, 2000) is representative of all of the class I PDE catalytic domains (ie, those present in mammals and flies; Ke and Wang, 2007). The catalytic domain (Pfam: PF00233) typically consists of 16 *α*-helices that bind 2 divalent metals, Zn^2+^ and (typically) Mg^2+^ that are essential for the catalysis of cAMP and cGMP (Fig. 3). Zn^2+^ coordinates with His^238^, His^274^, Asp^275^, and Asp^392^ of PDE4B2B, and 2 water molecules. Mg^2+^ chelates with Asp^274^ and 5 water molecules. The folding of the catalytic domain and metal interactions is conserved in all PDE families (Ke and Wang, 2006). Binding of the substrate in the active site, a conserved hydrophobic pocket, is stabilized not only by the hydrogen bond with invariant glutamine but also by interactions between the substrate’s purine ring and several hydrophobic residues residing in the, so-called, “hydrophobic clamp” (Ke et al, 2011) that positions the cyclic monophosphate group for hydrolysis of the phosphodiester bond by a nucleophilic attack (Wang et al, 2007a). For example, Phe^372^ stacks against the purine ring of cAMP in the structures of PDE4D2-AMP and D201N PDE4D2-cAMP on 1 side (Fig. 4C), whereas Phe^340^ and Ile^336^ make hydrophobic interaction with the purine on the other side (Wang et al, 2007a). The highly conserved hydrophobic pocket is primarily responsible for the high affinity and selectivity of family specific PDE inhibitors (Ke and Wang, 2007), as exemplified by the PDE4B2B—inhibitor NPV interaction that is called the “hydrophobic slot” (Hartley, 1964; Wang et al, 2007a). In contrast, several “subpockets” were proposed for the binding of PDE family selective inhibitors, as discussed in the later sections.

#### 2 Allosteric mechanisms for regulating cyclic nucleotide hydrolysis by PDEs

##### a General aspects

Perhaps the best-studied structural elements hypothesized to regulate catalytic activity of PDEs are the flexible H-loop (including 2 short *α*-helices) and the flexible M-loop [the flexible region between *α*14 and *α*15; for review see Ke and Wang (2007)]. Alignments of the catalytic domains of the entire superfamily suggest the involvement of these flexible regions in the regulation of substrate diffusion into the enzyme active site due to their vicinity to the entrance (Ke and Wang, 2007; Huang et al, 2015). However, the mechanisms for restricting substrate diffusion into the catalytic center differ between PDE families. Specific for PDE4, an *α*-helix downstream of the *α*16 helix in the catalytic domain putatively participates in phosphorylation-dependent auto-inhibition. The H- and M-loop can also undergo major conformational changes upon binding of PDE inhibitors to the active site, emphasizing their pharmacological importance in structure-aided drug design. In the following sections, we examine in detail the allosteric regulation of several PDE families with an emphasis on how knowledge of atomic-level PDE structures can inform and guide efforts to rationally design novel, family, and isoform-selective PDE inhibitors.

##### b PDE2

**Fig. 5:**
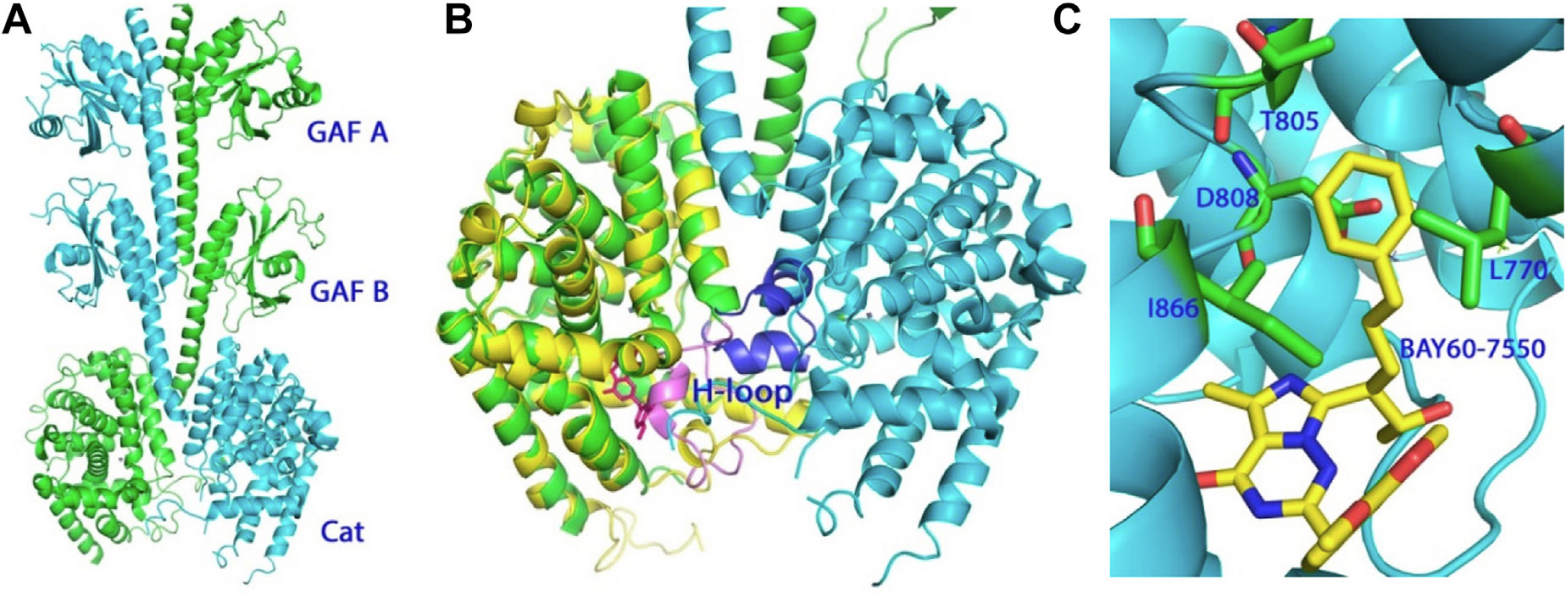
Structure of PDE2. (A) Dimer of PDE2A3 adapted from Pandit et al (2009). Catalytic subunits are shown in green and cyan. (B) Superposition of the unliganded nearly full-length PDE2 (green and cyan) over the PDE2 catalytic domain (yellow) complexed with inhibitor Bay60-7550 (red sticks; adapted from Zhu et al (2013)). The H-loop conformations are shown for the PDE2-Bay60-7550 structure (blue) and the unliganded structure (pink). (C) A unique hydrophobic pocket may aid in the future design of PDE2 inhibitors with high affinity and selectivity.

Structure studies (Martinez et al, 2002; Pandit et al, 2009) have revealed that cGMP binding at a flexible binding pocket within the GAF-B domain induces structural changes to enhance cGMP affinity. The X-ray structure of PDE2A (Pandit et al, 2009) shows that the 2 catalytic subunits cross over at the juncture of the GAF-B and catalytic domains (Fig. 5). The H-loop (residues Gly^702^–Ser^724^ of PDE2A) from the opposite subunit is in proximity to, and restricts substrate access to, the active site of each monomer. Binding of the inhibitor BAY 60-7550 to the active site caused an outward movement (and change in conformation) of the H-loop (Zhu et al, 2013). It has been hypothesized that cGMP binding to the GAF-B domain induces conformational changes within GAF-B, which after propagation through the *α*-helix and the adjacent catalytic domain changes the H-loop conformation to enhance substrate entry and, thus, its catalysis (Pandit et al, 2009; Zhu et al, 2013). This thesis awaits confirmation.

The structure of PDE2 in complex with BAY 60-7550 (Fig. 5C) identified a hydrophobic pocket consisting of Leu^770^, Ile^866^, and the hydrophobic side chains of The^805^ and Asp^808^ (Fig. 5C; Zhu et al, 2013). This knowledge may enable the design of PDE2-selective inhibitors in the future, as evidenced by the subsequent discovery of a novel PDE2 inhibitor (Qiu et al, 2018).

PDE2A is widely expressed, and most abundant in the brain (Lakics et al, 2010), implying application in CNS disorders, eg, depression and anxiety (Masood et al, 2009; Xu et al, 2013; Zhang et al, 2015). Because past studies were hampered by the lack of selective and brain-penetrant compounds further inhibitor design is required.

##### c PDE5

There is ample information to support the idea that PDE5 forms a homodimer with an overall domain organization similar to those of PDE2 (Fig. 5A) and PDE6 (Francis et al, 2006; Schultz, 2009; Ahmed et al, 2021; Cote et al, 2022). For the 5 PDE families containing 2 tandem GAF regulatory domains [GAF-A and GAF-B (Pfam PF01590); Aravind and Ponting (1997)], only 1 GAF domain per monomer binds cyclic nucleotides: cGMP binds to GAF-A in PDE5 PDE6, and PDE11 and to GAF-B in PDE2, whereas cAMP binds to GAF-B in PDE10 (Zoraghi et al, 2004; Heikaus et al, 2009; Schultz, 2009; Jager et al, 2012).

PDE5 catalytic activity is modulated by 3 allosteric mechanisms. First, PDE5 catalytic activity is stimulated upon binding of cGMP to noncatalytic sites localized to the GAF-A domain. The GAF-A binding site undergoes a major, local conformational change upon cGMP binding (Heikaus et al, 2008) that is allosterically communicated to the catalytic domain to cause an increase in cGMP hydrolysis. This allosteric effect is reciprocal in that occupancy of the PDE5 active site enhances the affinity of cGMP binding to GAF-A. In addition, cGMP binding to GAF-A stimulates phosphorylation of a serine residue in the *N*-terminal region that precedes the GAF-A domain; this effect is also reciprocal in that phosphorylation of PDE5 at this site enhances cGMP binding affinity to GAF-A and, in a cooperative manner, stimulates catalytic activity [for a comprehensive review, see Francis et al (2011)]. Delineation of the allosteric communication pathway from the regulatory to the catalytic domains of PDE5 awaits determination of the atomic structure of the full-length enzyme and identification of ligand-induced conformational changes.

**Fig. 6:**
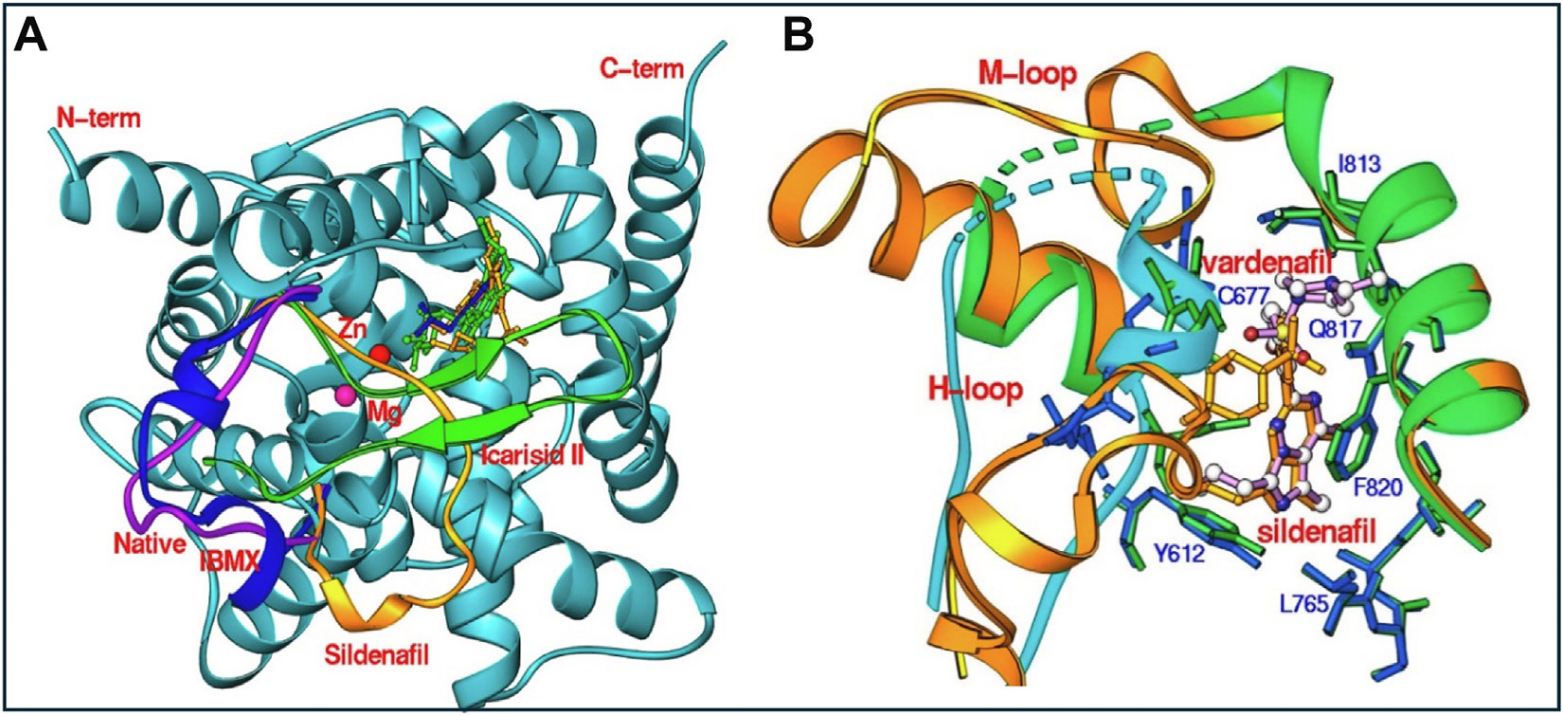
Conformational changes in the catalytic domain of PDE5 upon binding of inhibitors. (A) Dramatic conformational changes in the H-loop of the PDE5 catalytic domain upon binding of IBMX (blue), sildenafil (gold), icarisid II (green) or in the unliganded state (purple). (B) Superposition of PDE5 structures in complex with sildenafil (gold ribbons and sticks) and vardenafil (cyan), duplicated from Wang et al (2008b). The shared structural elements of PDE5 are shown in green.

The second intrinsic mechanism for allosteric regulation of PDE5 catalysis is observed in local conformational changes of the flexible elements around the active site (Fig. 6). The most important of these is the H-loop (residues 661–678 of PDE5A1) that adopts different conformations upon binding different inhibitors (Wang et al, 2006). The ligand-free H-loop has a coiled conformation and migrates dramatically to form a small 3_10_ helix upon binding of IBMX or sildenafil (Fig. 6A). Binding of the natural product, icarisid II (purified from the Chinese herb Ying Yang Hu which is known to promote sexual activity of goats) induced the formation of 2 short anti-parallel *β*-strands in the H-loop (Fig. 6A). In addition, sildenafil and vardenafil—2 PDE5 inhibitors with similar molecular structures—induce completely different conformational changes in both the H-loop and the adjacent M-loop motif [residues 790–810; see Fig. 5B, adapted from Wang et al (2008b)], possibly explaining their distinctive affinity for PDE5 and their physiological effects.

**Fig. 7:**
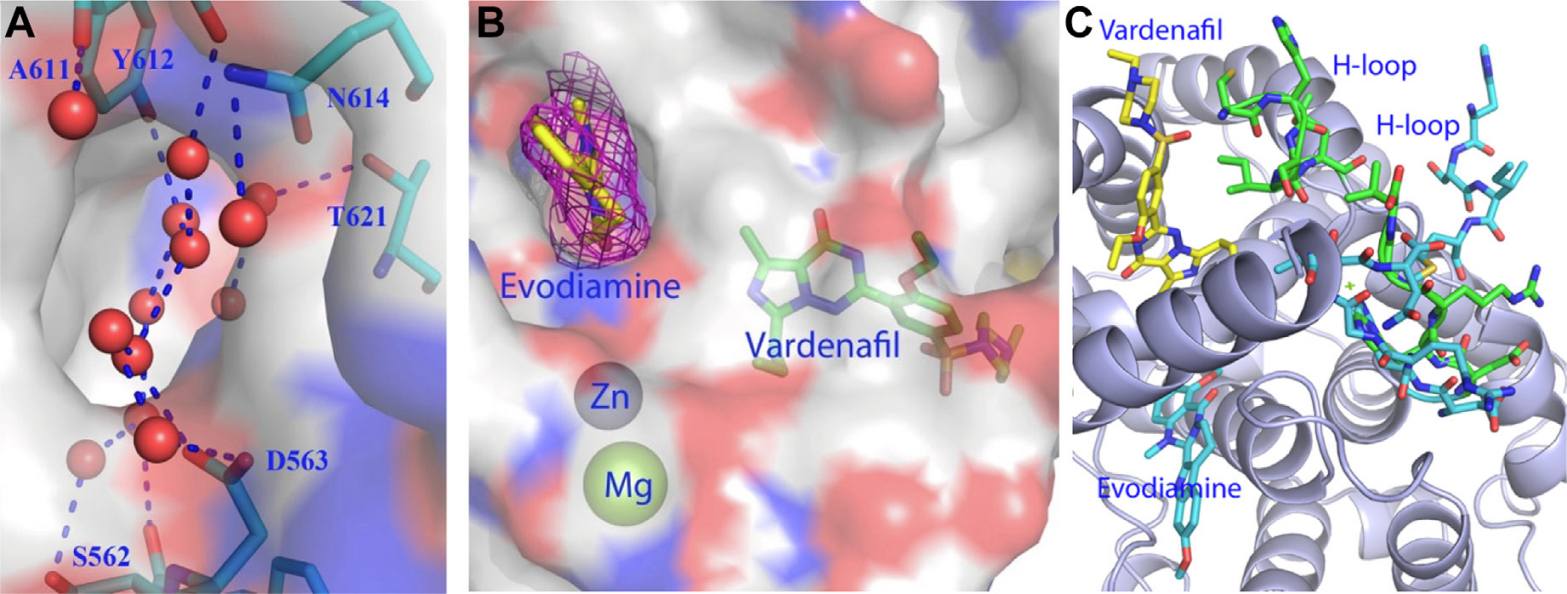
Binding of the natural product evodiamine to PDE5. Evodiamine occupies an allosteric effector site that adjoins the active site of PDE; adapted from Zhang et al (2020c). (A) Surface representation of the allosteric effector site in the catalytic domain that is filled with a bundle of water molecules (red spheres) in the unliganded state. (B) Surface representation of the binding of evodiamine. The purple mesh shows the electron density of the binding; vardenafil occupies the active site of PDE5. (C) Ribbon presentation of the conformational changes in the H-loop upon binding of vardenafil (green sticks) and evodiamine (cyan sticks); adapted from Zhang et al (2020c).

A third mechanism of regulating PDE5 activity has been identified for the allosteric inhibitor evodiamine, which upon binding to a site adjacent to the active site (Fig. 7) induces conformational changes in the active site (Zhang et al, 2020c). Binding of evodiamine displaces several water molecules occupying the unliganded binding site and induces conformational changes to the H-loop at the active site (Fig. 7C).

##### d PDE4

UCR1 and UCR2 participate in regulating cAMP hydrolysis in a coordinated manner. In long isoforms of PDE4, UCR1 and UCR2 interact to form a regulatory unit in which the *C*-terminal portion of UCR2 folds onto the catalytic domain and blocks substrate access to the active site (Burgin et al, 2010; Cedervall et al, 2015). As mentioned above, occlusion of the active site can also occur when a C-terminal *α*-helix folds onto the active site (Burgin et al, 2010).

The 4 genes that comprise the PDE4 family have very high (∼78%) sequence identity within the 340 amino acids of the catalytic domain. This is also reflected in the structural superposition of the catalytic domains of PDE4D with PDE4A, PDE4B, and PDE4C (root-mean-squared deviations of 0.67, 0.73, and 0.64 Å for the C*α* atoms; [Wang et al, 2007a]). Consequently, most PDE4 inhibitors do not discriminate between the 4 members of this family in their affinity for binding to the catalytic pocket. After it was suggested that inhibition of PDE4D produces many of the side-effects of the PDE4 inhibitor rolipram, developing family selective PDE4 inhibitors, especially against PDE4B, became an important medicinal chemistry objective (Azam and Tripuraneni, 2014). Moreover, attention has more recently turned toward the therapeutic potential of allosteric inhibitors that are more likely to bind specifically to a member of the PDE4 subfamily and may display an improved therapeutic ratio.

**Fig. 8:**
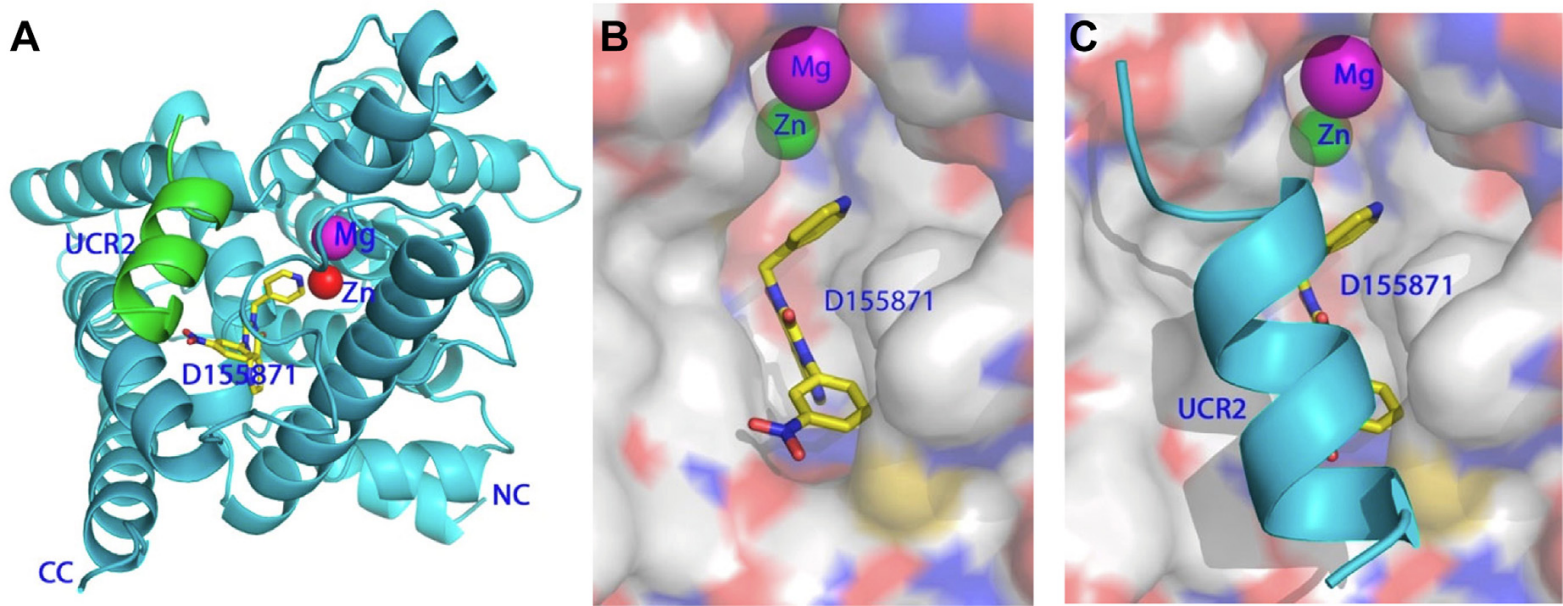
The structures of PDE4D in complex with the allosteric inhibitor D155871. (A) Ribbon presentation of the PDE4D catalytic domain (cyan) with a helix from the UCR2 region (residues 191-201 of PDE4D7, green) interacting with the inhibitor D155871 (yellow sticks); NC and CC, N- and C-termini, respectively, of the catalytic domain. Note that residues 204–254 that connect UCR2 helix and the catalytic domain have disordered conformations and are not traceable. (B) Surface representation of D155871 binding in the absence (panel B) and presence (panel C) of the UCR2 helix (Burgin et al, 2010).

Two examples of modulators of PDE4 catalytic activity that are not simple competitive inhibitors at the enzyme active site are D 155871 (Burgin et al, 2010) and zatolmilast (BPN 14770; Gurney et al, 2019), both of which inhibit the long forms of PDE4D. Binding of D 155871 to the active site of PDE4D (Fig. 8) induces dramatic migration of an *α*-helix of UCR2 from the back of the catalytic domain to cover the active site. However, it remains unclear if allosteric PDE4 inhibitors will have therapeutic applications.

##### e PDE9

The PDE9 catalytic subunit consists of a coiled *N*-terminal region and a conserved catalytic domain at the *C*-terminus and exists as a dimer. The crystal structure of PDE9 reveals that the catalytic sites in the 2 subunits have slightly different shapes, leading to different binding conformations of the same inhibitor in the dimer (Huang et al, 2015).

**Fig. 9:**
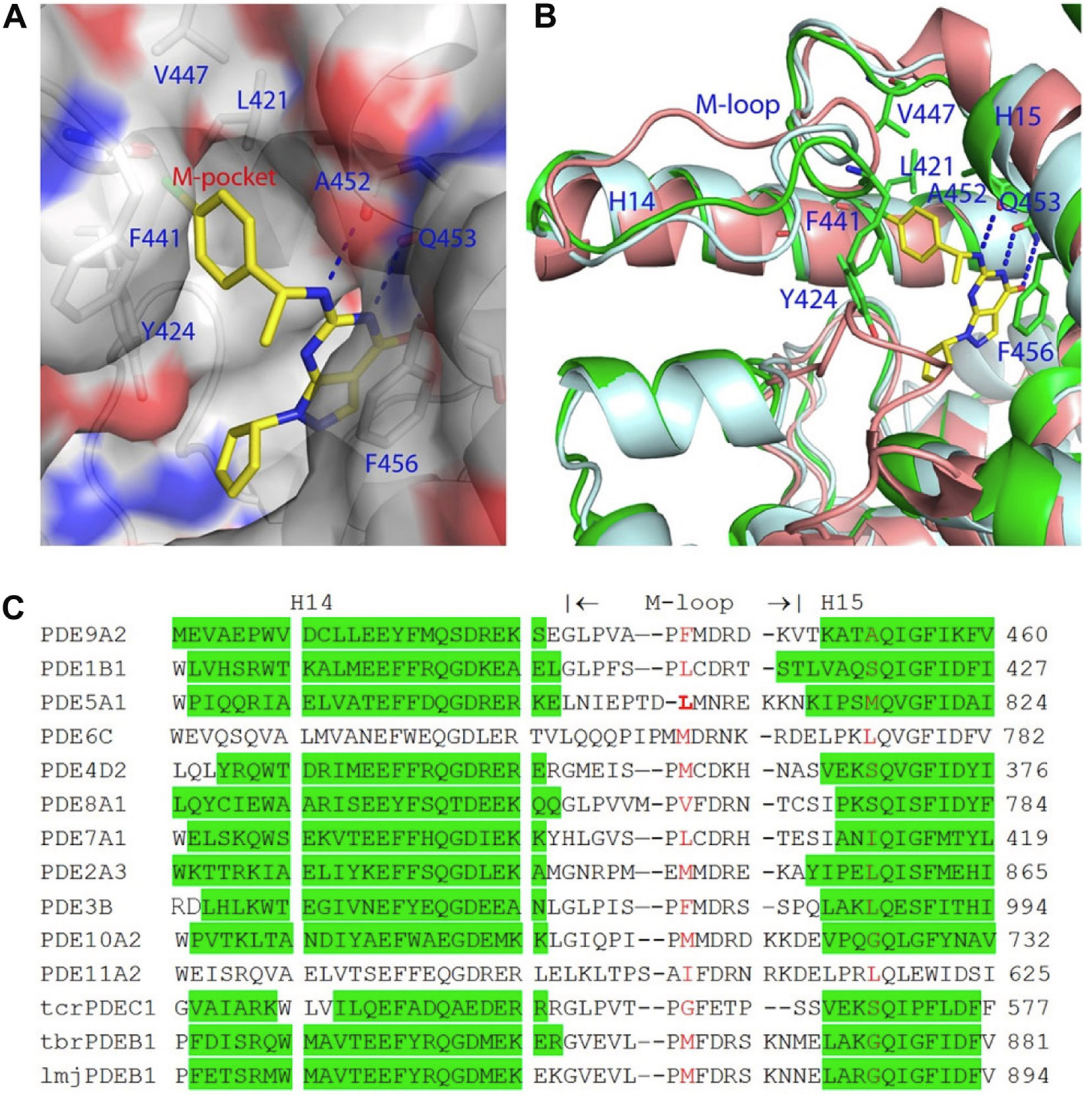
The M-pocket of PDE9 binds the inhibitor C33. (A) Surface representation of the PDE9 M-pocket occupied with C33 (yellow sticks). (B) Superposition of PDE9 (green ribbons) with PDE5 (light cyan) and PDE8A (salmon) showing structural conservation of the M-pocket. (C) Sequence alignment around the M-pocket of PDEs. The green color highlights helices H14 and H15 which are connected by the M-loop. Two residues (red) gate the pocket. Duplicated from Huang et al (2015).

Examination of inhibitor binding suggests that a small “M-pocket” may determine the selectivity of PDE9 inhibitors (Huang et al, 2015). The M-pocket is in an open conformation in PDE9, enabling large functional groups such as benzene to bind (Fig. 9A). In contrast, sequence alignment of PDEs in the vicinity of the M-pocket (Fig. 9C) illustrates that Phe^441^ and Ala^452^ of PDE9 (that serve as gates for the M-pocket) are substituted with bulky side chains in PDE5 (Leu^804^ and Met^816^) that would allow only small functional groups to penetrate into the M-pocket, as shown for the ethoxy group in the PDE5-sildenafil crystal structure (Wang et al, 2008b). In the case of the PDE8A1 structure (Wang et al, 2008a), the M-pocket is too small to accommodate binding of the PDE9 inhibitor, C33, providing a structural basis for the observed selectivity of C33 over PDE5 and PDE8 (Huang et al, 2015). In summary, the M-pocket of PDE9 may be an excellent target for the structure-aided design of highly selective PDE9 inhibitors.

#### 3 Peptide inhibitors, regulators, and activators

In addition to cyclic nucleotide binding, post-translational modifications, and the various pharmacological PDE inhibitors that have been characterized, other methods of altering PDE activity have been used as research tools, namely: (1) techniques for PDE depletion, (2) activators or methods that upregulate PDE protein, and (3) techniques that displace a defined PDE “pool” from 1 specific nanodomain within a cell.

##### a Depletion of endogenously expressed PDEs: RNA and DNA-based mechanisms

To define the role of specific PDEs, overexpression by constructs encoding full-length PDE proteins or, conversely, gene silencing using antisense or small interfering (si) RNA oligonucleotides has been employed. Detrimental to interpretation, overexpression can lead to aberrant cellular localization of the enzyme, and as is apparent from earlier sections of this review (see sections PDE1 Family and PDE11 Family), the localization of a cAMP-PDE is central to its function. Antisense oligonucleotides offered the first method to specifically target PDE isoforms (Epstein, 1998), and this approach has also been used to target different PDE families concomitantly for therapeutic benefit (Fortin et al, 2009). Soon, the development of siRNA sequences to evaluate the functional relevance of a variety of PDEs followed, as exemplified by PDE2, PDE4D, PDE7, and PDE8 (Pekkinen et al, 2008; Li et al, 2011; Hiramoto et al, 2014).

Another method for PDE silencing that has come to the fore recently is CRISPR/Cas9. It has been used to characterize the differential roles of PDE2A isoforms and PDE3A in shaping cAMP dynamics in neonatal and adult rat cardiomyocytes following *β*-adrenoceptor stimulation (Skryabin et al, 2023). The ability to pinpoint isoforms rather than subfamilies or families may lead to the development of therapies that result in fewer side-effects. This is particularly relevant for PDE4D isoforms in Alzheimer disease (AD) where a body of work using knock-out animals (Li et al, 2011), dominant negatives (Bolger et al, 2020), siRNA (Li et al, 2011), and PDE4D-selective inhibitors (Ricciarelli et al, 2017) has identified the subfamily as playing a crucial role. Recently, CRISPR/Cas9 genome editing has determined that specific silencing of the long PDE4D isoforms, PDE4D3, D5, D7, and D9, confers protection against *β*-amyloid-induced reductions in neuronal plasticity (Paes et al, 2023).

MicroRNAs (miRNA) are endogenous regulators of protein expression. Specific miRNA has been identified for PDE4 in synovial fibroblasts modulating proinflammatory processes (Wade et al, 2019), PDE3 in cerebral microvascular endothelial cells affecting cognitive decline in cerebral small vessel disease (Yasmeen et al, 2019) and PDE1 in lung fibroblasts where the miRNA is protective against lung fibrosis (Ren et al, 2017). miRNAs are, themselves, known to be regulated by long noncoding RNAs (lncRNA) and lncRNA GAS5 “sponges” miRNA that prevents the translation of *PDE4B2* to increase expression of the PDE, preventing the accumulation of lipid in cell models of nonalcoholic fatty liver disease (Xu et al, 2022).

##### b PDE (in)activation by manipulation at the protein level: proteolysis targeting chimeras (PROTACs)

Besides targeting DNA and RNA, there is a novel way to specifically reduce the activity of active enzymes in a cell via targeted protein degradation. This involves a small molecule that can link the enzyme of interest to the ubiquitin proteasome system by anchoring a ubiquitin E3 ligase close to the target. These small-sized molecules, of which the pharmacokinetic properties can be optimized for use in vivo, partly independently of Lipinski’s rule of 5, have been termed proteolysis targeting chimeras (PROTACs) and are now in phase II clinical trials for oncology (Wang et al, 2023). PROTACs appear to be ideal as PDE inhibitors (Konstantinidou et al, 2019). First, because rather than relying on the constant presence of high concentrations, a reason inhibitors cause side effects, to warrant occupation of the active site, PROTACS deactivates the PDE in 1 event. Second, protein degradation attenuates the enzymatic and nonenzymatic functions, allowing also the inhibition of PDEs as scaffolds for protein interactions (Susuki-Miyata et al, 2015). Finally, as the PROTAC molecule is formed of 3 integral units (warhead, linker, and E3 recruiter molecule), enhanced selectivity and potency can be engineered into the structural design via molecular modeling, providing a better opportunity for isoform specificity.

PROTACs that evoke degradation of the Kirsten Rat Sarcoma Virus (KRAS)-shuttling PDE6*δ*, a PDE6 subunit without enzymatic function, results in mislocalization of KRAS preventing its activation (Zhang et al, 2005a; Cheng et al, 2020; Teng et al, 2022). This has been a promising strategy to treat KRAS mutation-related cancer. “SNIPER” protein erasers demonstrated that a PDE4 inhibitor could be used as a warhead to colocalize E3 ubiquitin ligases for PDE4 degradation (Ohoka et al, 2017). The work might inspire the development of PROTACS for the other families.

##### c PDE (in)activation by manipulation at the protein level: disruptors of PDE compartmentalization

Cellular expression of cAMP-PDEs is often low but highly localized in order to shape cAMP in nanodomains (Baillie, 2009). Peptide disruptors that relocate cAMP-PDEs allow researchers to define the role of specific isoforms [reviewed in Blair and Baillie (2019), Lee et al (2013)]. As single isoforms can exist in more than 1 cellular locale and have multiple functions depending on proximity to various cAMP effector proteins (eg, PDE4D5; Wills et al (2016)], displacement is the only method that takes compartmentalization into account. This benefit was demonstrated recently when revealing the role of PDE4-Popeye domain-containing 1 interaction in nanodomain calcium transient regulation for sinoatrial pace-making (Tibbo et al, 2022). The use of a pan-PDE4 inhibitor would not have been as effective due to pools of various PDE4 subfamilies and isoforms associated with cardiac calcium handling (Maurice et al, 2014).

The use of catalytically dead, dominant-negative PDEs that displace endogenously active forms is applicable in transfected cell lines (McCahill et al, 2005) and whole organism models of disease (McGirr et al, 2016; Bolger et al, 2020). Although this approach involves PDE displacement, it also relies on overexpression; hence, it is likely all complexes containing the PDE of interest being overexpressed as a dominant negative will be disrupted concomitantly, limiting the deconvolution of data pertaining to compartmentalized responses. In the case of PDE11A, compartmentalization can be changed by disrupting homodimerization via the expression of its isolated GAF-B domain or by phosphorylation of select residues in its regulatory *N*-terminal domain, both of which are sufficient to alter memory formation in mice [see II Chapter 2, E 1k for more detail; (Pathak et al, 2017; Pilarzyk et al, 2022, Pilarzyk et al, 2023)].

##### d PDE (in)activation by manipulation at the protein level: molecules that enable increases in PDE activity

Recently, Mironid developed small molecules that activate PDE4 via allosteric binding to the UCR 1/2 regions to lock the long-form dimer in the “open” conformation (Omar et al, 2019). Activation of PDE4 long-forms in this way is sufficient to counteract chronic cAMP elevation. Application in polycystic kidney disease and prostate cancer has been proposed for these compounds (Hansen et al, 2022; Gulliver et al, 2023). This activation mechanism has been observed historically with lipids (Grange et al, 2000) and peptides (Wang et al, 2015a), and now, the small molecules have opened the door for the development of therapies that require PDE activation. Another technologically advanced way to enhance the protein stability of active enzymes is by using the converse approach to PROTACs. Targeted protein stabilization using deubiquitinase-targeting chimeras can stabilize target proteins by preventing them from engaging with the proteosome (Henning et al, 2022). This relatively new approach for selective PDE isoform activation holds promise for the future.

### D Role of PDEs in cyclic nucleotide compartmentalization

#### 1 cAMP compartments: the birth of a new rationale

The canonical model stating that cAMP signaling links G-protein coupled receptor (GPCR) ligand binding to cAMP generation, PKA activation, and substrate phosphorylation in a linear cascade from the plasma membrane to the terminal intracellular effector cannot explain the functional versatility of cAMP within 1 cell type. This enigma persisted for decades, until an alternative model developed over the last 20 years: the paradigm of compartmentalization. This spatial confinement proves critical for hormonal specificity, with G_s_-coupled receptor activation generating spatially distinct cAMP pools that, in turn, activate defined subsets of the effector enzyme PKA.

Selective PKA activation hinges on the immobilization of the kinase to specific subcellular locations through interaction with AKAPs, tethering the enzyme in proximity to specific phosphorylation targets (Scott et al, 2013). AKAPs nucleate signaling hubs, or signalosomes, through protein-protein interactions that include varying assortments of signaling molecules such as GPCR, adenylyl cyclases, phosphatases, and cAMP-PDEs. Each signalosome results from a unique combination of signaling components, facilitating specific regulation of distinct cellular functions at distinct locations. This paradigm started from studies on cardiac *β*-adrenoceptors and has since spread to virtually all cell types (Zaccolo et al, 2021).

Early imaging studies demonstrated the critical role of cAMP-hydrolyzing enzymes in limiting the spatial propagation of the second messenger as inhibition of PDEs disrupted the local cAMP gradients (Jurevicius and Fischmeister, 1996; Zaccolo and Pozzan, 2002; Terrin et al, 2006; Iancu et al, 2007; Chen et al, 2008; Leroy et al, 2008; Feinstein et al, 2012; Mika et al, 2012; Guellich et al, 2014). The advent of genetically encoded probes for real-time cAMP monitoring was a breakthrough in demonstrating the steep intracellular cAMP gradients that create nanodomains (Zaccolo et al, 2000; Zaccolo and Pozzan, 2002; Nikolaev et al, 2004; Ponsioen et al, 2004). These probes typically consist of a cAMP-binding domain sandwiched between 2 fluorescent protein spectral variants, enabling Förster resonance energy transfer (FRET) imaging. The altered FRET signaling that occurs upon cAMP binding is detectable with a conventional optical microscope. The first reported probe consisted of a PKA regulatory subunit fused to a cyan fluorescent protein and a PKA catalytic subunit fused to a yellow fluorescent protein, allowing measurement of cAMP binding to the PKA holoenzyme (Zaccolo and Pozzan, 2002). On application of noradrenaline to cardiac myocytes expressing the probe, the elicited cAMP response was not homogeneous across the cell but was localized to subcellular compartments where PKA is anchored to AKAPs. In agreement, a probe variant lacking the domain necessary for AKAPs anchoring resulted in the sensor being unable to pick up any significant cAMP signal. Studies using these biosensors demonstrated that different GPCRs couple with distinct adenylyl cyclases, generating specific local cAMP pools that activate a limited subset of effectors, thus creating functional diversity between GPCRs (Di Benedetto et al, 2008).

Further compartmentalization mechanisms include physical barriers (Richards et al, 2016), buffering (Bock et al, 2020), phase separation of the regulatory subunit of PKA (Zhang et al, 2020a), and generation of cAMP from internalized GPCRs (Lohse et al, 2023).

#### 2 Organization in signalosomes and participation of PDE

Recent mounting evidence that G_s_PCRs can initiate cAMP production postinternalization introduces a novel and intriguing aspect to the cAMP compartmentalization model (Calebiro et al, 2009; Ferrandon et al, 2009; Irannejad et al, 2013). Traditionally, internalization of GPCRs upon ligand binding has been considered a mechanism that leads to receptor desensitization, offering protection against excessive stimulation. However, recent studies combining real-time cAMP imaging with observations of GPCR trafficking events in intact cells, challenged this notion, as they demonstrated sustained cAMP signaling from G_s_PCRs embedded in internalized vesicles (Ferrandon et al, 2009; Calebiro et al, 2010; Lan et al, 2012; Pavlos and Friedman, 2017). Internalized GPCRs were shown to encounter G proteins on internal membranes, such as endosomes or Golgi-associated vesicles, and create a fully functional signaling complex initiating a subsequent wave of signaling by triggering the generation of localized pools of second messenger. Alternatively, a substantial pool of some GPCRs, such as *β*_1_-adrenoceptor, can be permanently located at intracellular membranes, as, for example, in the SR of cardiac myocytes to locally regulate contractility and relaxation (Lin et al, 2023b).

**Fig. 10:**
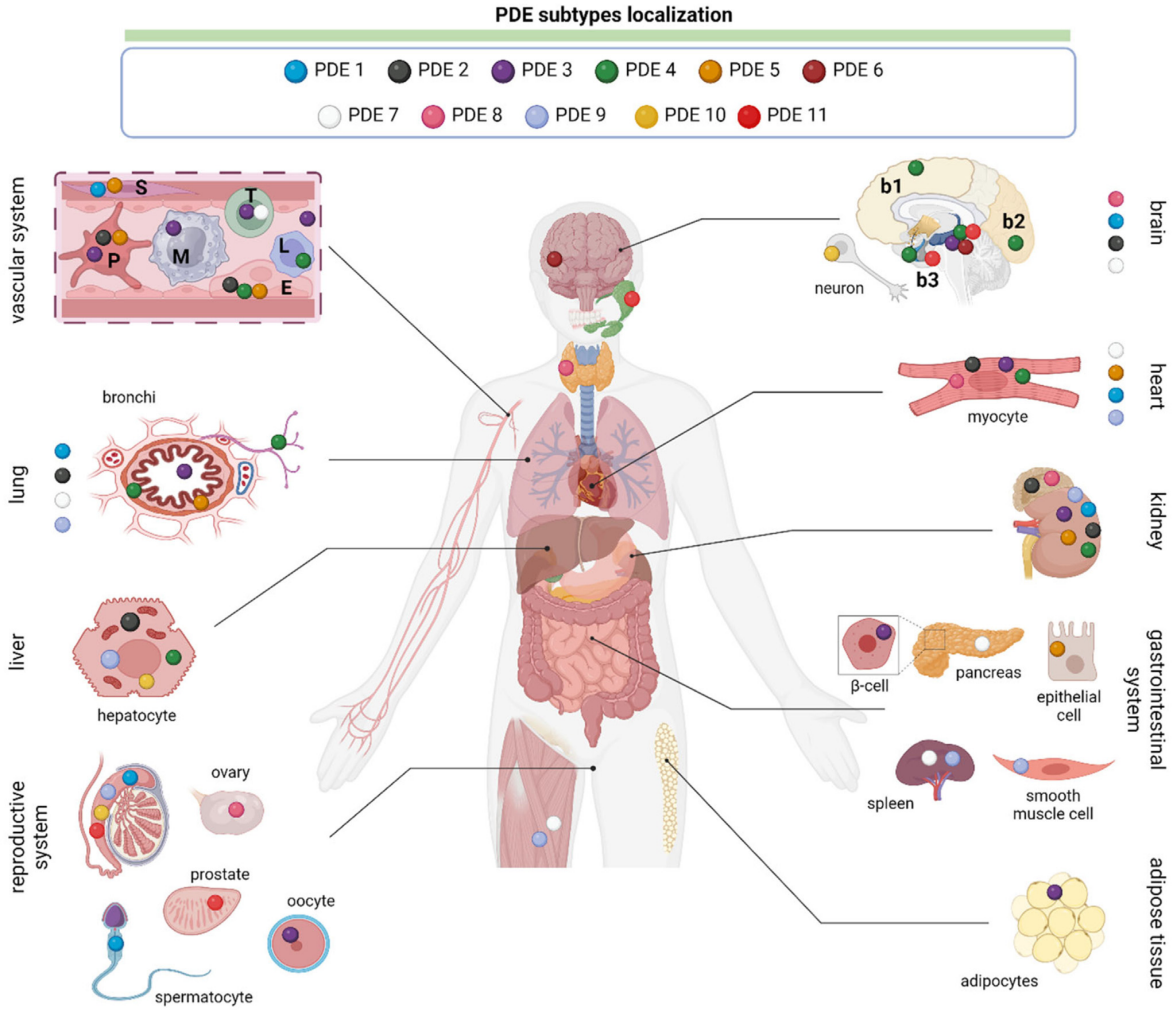
Mapping the tissue localization of PDEs. This figure illustrates the diverse tissue localization of PDEs, enzymes crucial for regulating cyclic nucleotide levels. PDE expression patterns vary among species and can be altered in pathological conditions, influencing various physiological processes such as cardiovascular function, neuronal signaling, immune responses, and reproductive function. In cases where the precise tissue localization of PDEs is unknown, their representation is depicted vertically adjacent to the respective tissue name. b1, cerebral cortex/temporal lobe; b2, ocipital lobe; b3, temporal lobe; E: endothelial cells; L: lymphocytes; M, macrophages; P, platelet; S, smooth muscle cells; T, T-cells. Created with BioRender.com.

The complexity of the PDE system allows for a further highly sophisticated regulation of local cAMP levels. The number of possible permutations in combinations of PDE isoform, location, activity regulation by Ca2+, cGMP, or other mechanisms is extensive and can all determine the subcellular topography of cAMP nanodomains (Beltejar et al, 2017; Subramaniam et al, 2023; Fig. 10). The resulting dynamic cellular landscape of cAMP gradients remains largely to be defined in its details as is any remodeling that occurs in pathological conditions.

#### 3 Size matters, when measuring cAMP in nanodomains

The organizational complexity of PDEs and their interactions to generate local steep cAMP gradients proves highly effective for achieving hormonal specificity. Compartmentalized cAMP in cardiac myocytes, for example, allows *β*-adrenoceptors to increase PKA-dependent phosphorylation of phospholamban, whereas activation of the prostaglandin receptor does not, despite generating a similar overall cAMP amount (Hayes et al, 1980). Targeted FRET reporters further demonstrated that distinct cAMP signals and heterogeneous cAMP responses are generated by the activation of different cardiac GPCRs with positive inotropic effects (Di Benedetto et al, 2008), By contrast, a homogeneous cAMP increase throughout the cell, achieved through pharmacological PDE inhibition, results in a diminished inotropic response (Surdo et al, 2017). This emphasizes the functional relevance of this spatial organization.

As an alternative to nanodomain-targeted sensors, recent developments using integrated analysis of PDE isoform-selective interactomes and PDE family dependent phosphoproteomes allowed the identification of multiple novel, nonobvious cAMP subcellular nanodomains (Subramaniam et al, 2023). Early imaging experiments estimated the size of cAMP subcellular compartments to be in the micrometer range (Zaccolo and Pozzan, 2002). Recent studies, however, revealed that the cAMP subcellular domains can be as small as tens of nanometers (Surdo et al, 2017; Anton et al, 2022). FRET reporters that include spacers with known length (nanorulers) revealed cAMP compartment sizes as small as 10 nm, ruling out the necessity for a physical barrier to achieving cAMP compartmentalization (Anton et al, 2022). Experiments with cAMP reporters fused directly to PDEs (Herget et al, 2008) demonstrated that PDEs can create regions of low cAMP concentrations, obliterating PKA activation within their immediate surroundings. The action radius varies based on the enzymatic properties of the involved PDE isoforms. For instance, PDE4A1, a relatively high-affinity (low *K*_m_) but low-turnover (low *V*_max_) enzyme (Bender and Beavo, 2006), creates a domain with low cAMP with a 10-nm radius, whereas PDE2A3, a lower affinity but faster enzyme, can deplete cAMP within a radius exceeding 30 nm (Bock et al, 2020). Recent development of nanodomain-targeted cAMP sensors provided more detailed insights into cAMP signaling (DiPilato et al, 2004; Allen and Zhang, 2006; Liu et al, 2011; Lefkimmiatis et al, 2013; Pendin et al, 2017; Surdo et al, 2017). Sensors targeted to plasma membrane nanodomains (Perera et al, 2015; Bastug-Özel et al, 2019) and SR domains in close proximity to the ryanodine receptor (Berisha et al, 2021) and SR Ca^2+^ ATPase (Sprenger et al, 2015) could reveal that cardiac hypertrophy and heart failure lead to a dramatic alteration of subcellular localization of multiple PDEs, resulting in changes of contractility, relaxation, and cardiac arrhythmias. Therefore, a better understanding and specific targeting of such nanodomain remodeling may pave the way to new approaches for cardiovascular therapies. Possible approaches that have been proposed include overexpression of distinct PDE isoforms (Karam et al, 2020), their specific knockdown (Skryabin et al, 2023), or other means aimed at the restoration of proper nanodomain architecture. Obviously, the future of thorough unraveling of cAMP-PDE signaling depends on the development of sophisticated, high-resolution sensors.

## II Chapter 2: Physiology and clinical development

### A Modulation of cardiac function by PDEs

#### 1 Introduction: (patho)physiology of the heart related to cyclic nucleotide signaling

Heart failure and reduced ejection fraction (HFrEF) represent a persistent clinical syndrome characterized by the gradual decline of cardiac function. Ultimately, the heart’s ability to pump blood efficiently becomes inadequate to meet the body’s oxygen demands, resulting in organ failure and, in severe cases, death. HFrEF can be caused by various factors, including arrhythmias, cardiomyopathies, coronary artery disease, congenital heart defects, infections, hypertension, valve problems, and the cardiotoxicity of certain anticancer drugs (McDonagh et al, 2022). Regardless of its origin, reduced cardiac function triggers the activation of neurohormonal systems and the development of cardiac hypertrophy to normalize ventricular wall stress (Hartupee and Mann, 2017). However, this compensated state is usually short-lived and gradually progresses toward chamber enlargement, eventually leading to HFrEF.

In normal physiological conditions, cAMP and cGMP have contrasting roles in cardiac function. *β*-adrenoceptor-stimulated cAMP release increases cardiac output through PKA. Conversely, nitric oxide (NO) or natriuretic peptide (NP) -induced cGMP can exert both synergistic and opposing effects on cAMP. In HF, decreased cardiac function chronically elevates sympathetic catecholamines (Cohn et al, 1984). This initiates a pathophysiological “vicious circle” of excessive *β*-adrenoceptor stimulation, explaining the beneficial effects of *β*-blockers in HFrEF (El-Armouche and Eschenhagen, 2009). Continuous cAMP-PKA signaling triggers the maladaptive remodeling leading to HFrEF, featuring hypertrophy, cardiomyocyte death, and fibrosis. Conversely, NO and NPs are anti-hypertrophic and anti-fibrotic. Therefore, NP-, NO- and cGMP-increasing drugs enhance conventional treatments for patients with HFrEF (McMurray et al, 2014; Armstrong et al, 2020; Petraina et al, 2022).

PDE control of cyclic nucleotides in nanodomains with PKA-AKAP is pivotal in cardiac function regulation (see section Role of PDEs in Cyclic Nucleotide Compartmentalization; Kokkonen and Kass, 2017; Bock et al, 2020; Anton et al, 2022). The nanodomain organization undergoes significant remodeling in cases of pathological hypertrophy and HF, believed to contribute to heart function deterioration. In HFrEF, changes in PDE expression, activity, and subcellular localization alter cAMP and cGMP signaling and are associated with modulated *β*-adrenoceptor signaling, decreased NO bioavailability, and impaired NP signaling (Lohse et al, 2003; Katz et al, 2005; Nikolaev et al, 2010; Dickey et al, 2012). PDE3 and PDE5 inhibitors have shown detrimental effects in HFrEF patients (Packer et al, 1991) and a lack of efficacy in heart failure with preserved ejection fraction (HFpEF; Redfield et al, 2013). However, ongoing research suggests that specific PDEs could be potential therapeutic targets to prevent cardiac remodeling and HF (Preedy, 2020; Chen and Yan, 2021).

**Fig. 11:**
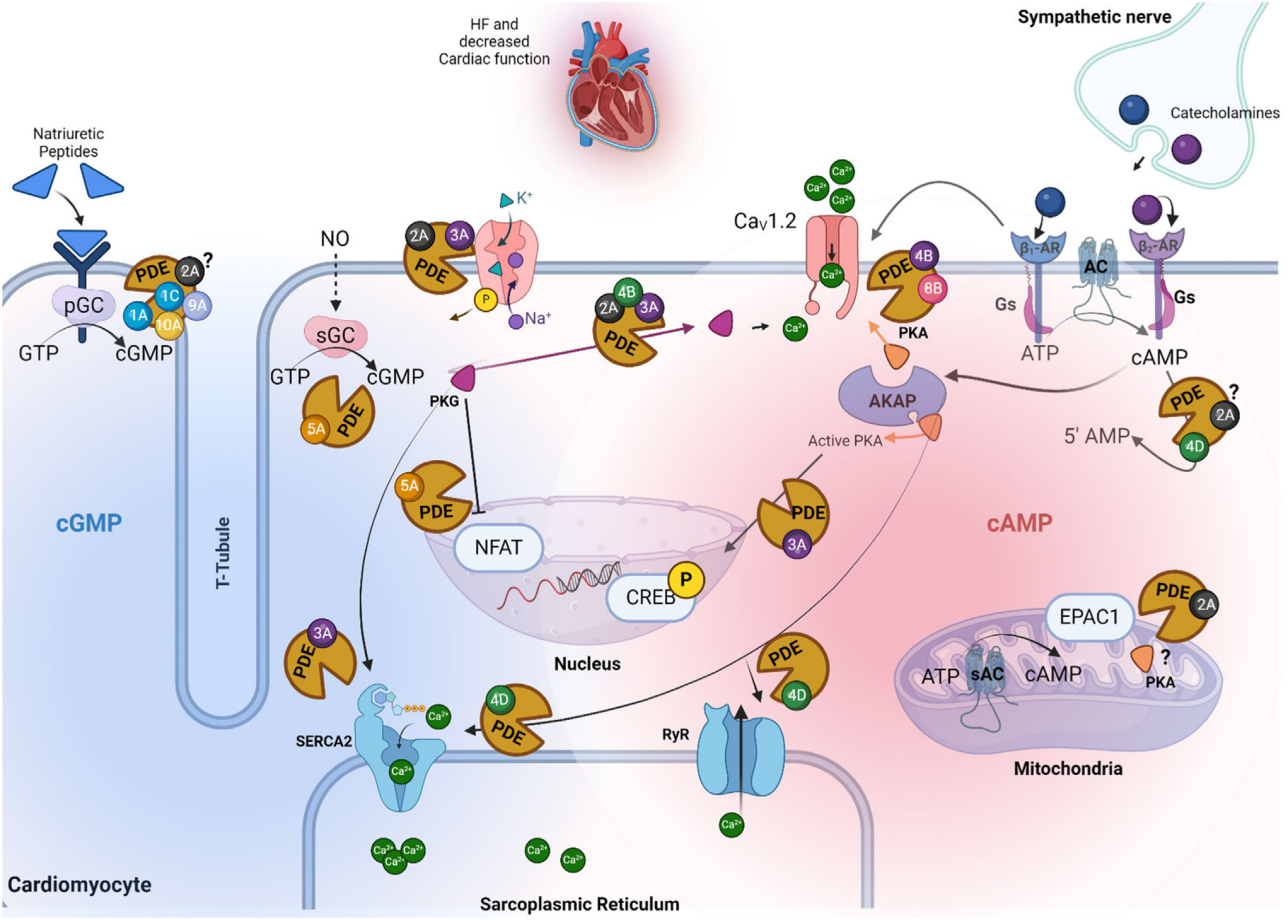
Modulatory role of PDEs on cAMP/cGMP signaling in cardiomyocytes. In this figure, the red area denotes the presence of cAMP, while the blue area signifies the contribution and subcellular localization of cGMP PDEs. Each PDE sub-family is represented as small colored circles on the PDE enzyme. Under normal physiological conditions, cardiac function is finely tuned by 2 intracellular cyclic nucleotides with opposing effects: cAMP, influenced by beta-adrenergic stimulation and protein kinase A signaling, and cGMP, stimulated by nitric oxide (NO) and natriuretic peptides (NPs) production. However, in pathological conditions such as heart failure, chronic elevation of catecholamines due to sympathetic over activation further stimulates beta-adrenergic receptors, leading to maladaptive remodeling, including hypertrophy and cardiac fibrosis. Conversely, elevated levels of NO and NPs exhibit anti-hypertrophic and anti-fibrotic effects. The question marks denote uncertainty regarding whether specific PDEs play preventive roles in these processes. Created with BioRender.com.

PDE1, PDE2, PDE3, PDE4, PDE5, PDE8, PDE9, and PDE10 are expressed in the heart, and all have a functional role. Much of the work and details of how these PDEs influence the normal and diseased heart can be found in several recent reviews (Preedy, 2020; Kamel et al, 2023). Here, we focus on recent advances (Fig. 11).

#### 2 PDE regulation of cardiac function through cAMP

##### a PDE1

Of the PDE1 isoforms, only PDE1A and PDE1C are expressed in the heart. PDE1A dominates in the myocardium of rat and mouse and preferentially hydrolyzes cGMP (Miller et al, 2009; Miller et al, 2011). PDE1C has balanced selectivity for cGMP and cAMP in cell-free conditions, but in myocytes and intact hearts, it primarily impacts cAMP (Knight et al, 2016; Hashimoto et al, 2018). In large mammal and human hearts, PDE1C is more prominent and constitutively expressed, providing most of the basal cGMP and cAMP hydrolysis activity measured in soluble cardiomyocyte fractions (Vandeput et al, 2007; Muller et al, 2021). PDE1C was further shown to reside in a complex with the adenosine A_2A_ receptor (coupled to cAMP formation) and the transient receptor potential channel 3 (TRPC3) that mediates Ca^2+^/CaM-dependent activation of PDE1C (Zhang et al, 2018b).

Both PDE1A and PDE1C expressions rise in human and animal models of HF (Miller et al, 2009; Knight et al, 2016; Wu et al, 2017). Genetic deletion of PDE1C in mice generates no basal phenotype but is protective against pressure overload hypertrophy and fibrosis via cAMP-PKA and phosphatidylinositol 3-kinase/Akt-dependent pathways (Knight et al, 2016). Fibrosis was also reduced. Fibroblasts do not express PDE1C, suggesting a paracrine mechanism. One to 2 weeks of treatment with IC 86340 or vinpocetin attenuated heart disease caused by angiotensin II stimulation (Wu et al, 2017), CryAB^R12G^ induced proteinopathy (Zhang et al, 2019) and doxorubicin toxicity (Zhang et al, 2018b). This benefit, coupled with cAMP-PKA signaling, implicates the activation of Akt, the proteosome, and antiapoptotic pathways, respectively.

In both conscious dogs and intact rabbits, selective PDE1 inhibition (with lenrispodun [ITI-214]) results in enhanced contractility and relaxation, reduced arterial resistance, and an elevated heart rate (Hashimoto et al, 2018). The latter involves *β*-adrenoceptors, whereas enhanced contraction-relaxation implicated PDE1-adenosine A_2_ receptor coupling (Hashimoto et al, 2018). In isolated myocytes, PDE1 inhibition only augmented voltage-gated calcium conductance through Ca_V1.2_ channels but did not increase the phosphorylation of phospholamban, troponin I, or myosin binding protein C or raise sarcoplasmic reticular Ca^2+^ load (Muller et al, 2021). In contrast, PDE3 inhibition augments Ca_V1.2_, phospholamban, and MyBPC phosphorylation induced by *β*-adrenoceptor stimulation (Mika et al, 2013; Muller et al, 2021). Compared with PDE3 inhibition, suppressing PDE1 led to a smaller increase in intracellular calcium transients and correspondingly less arrhythmia. These findings spawned the only clinical test of a PDE1 inhibitor in human HF (Gilotra et al, 2021), a single-dose placebo randomized protocol in which lenrispodum increased ventricular power index, cardiac output, and heart rate and lowered vascular resistance.

##### b PDE2

PDE2 activity and expression are increased in human myocardium from patients with end-stage HF (Mehel et al, 2013). This is also seen in isoprenaline-induced HF in rats (Kaumann et al, 2009; Mehel et al, 2013). PDE2 hydrolyzes cAMP in the vicinity of SERCA2a in hypertrophied cardiomyocytes (Sprenger et al, 2015). PDE2 increase results in reduced *β*-adrenoceptor-mediated hypertrophic remodeling induced by noradrenaline and phenylephrine (Mehel et al, 2013). Thus, PDE2 activation might be cardioprotective, as was confirmed in cardiac-specific PDE2-overexpressing mice for catecholamine-induced ventricular tachycardia and for cardiac dysfunction after myocardial infarction (Vettel et al, 2017). Conversely, PDE2 inhibition with BAY 60-7550 increased susceptibility to arrhythmias after reperfusion injury of isolated mouse hearts (Wagner et al, 2021). In this same study, the increase in PDE2 expression specifically in the heart prevented the incidence of depolarizations induced by isoprenaline, a pathogenic cause of arrhythmia (Wagner et al, 2021). Accordingly, gene therapy with PDE2A overexpression was shown to limit cardiac adverse left ventricle remodeling, dysfunction, and arrhythmias induced by catecholamines. Hence, strategies that increase PDE2A activity could prevent progression toward HF (Kamel et al, 2023).

PDE2A is a cGMP-activated PDE (Martins et al, 1982), which might account for its protection against HF (Numata and Takimoto, 2022). Activation of the particulate guanylyl cyclase localized near PDE2A (Castro et al, 2006) results in a NP/BNP/cGMP-triggered defense mechanism during cardiac stress particularly during the excessive *β*-adrenoceptor-mediated drive. Interestingly, sacubitril, a neutral endopeptidase inhibitor that elevates NPs, improves classical treatments for HF (McMurray et al, 2014), and NPs exert antiarrhythmic effects via PDE2 (Cachorro et al, 2023).

In paradox, some studies have shown the detrimental effects of PDE2 activation. PDE2 upregulation was found to be prohypertrophic as the PDE2 inhibitor BAY 60-7550 antagonized cardiomyocyte growth via PKA phosphorylation of nuclear factor of activated T cells (NFAT), preventing the activation of the hypertrophic gene program (Zoccarato et al, 2015), and protected against apoptosis by promoting mitochondrial elongation (Monterisi et al, 2017). PDE2 activity and expression were upregulated in hypertrophied mouse myocytes after pressure overload and isoprenaline, and BAY 60-7550 decreased left ventricular (LV) hypertrophy, LV dilation, contractility, and fibrosis (Baliga et al, 2018). In addition, BAY 60-7550 or overexpression of catalytically inactive forms of PDE2 led to a restoration in the modulation of noradrenaline release in the stellate ganglion by BNP, which might further protect against HF (Liu et al, 2018).

The described paradox may be attributed to differences in the PDE2 isoforms expressed, the constructs used, or the cellular localization of the heterologous PDE2. More generally, the discrepancies may depend on the etiology of the HF. Further studies are needed to clarify whether inhibition or activation of the enzyme is preferred, implicating this difference in etiology.

##### c PDE3

PDE3 is expressed in the myocardium of many species and is particularly abundant in large mammals including humans. Thus, PDE3 inhibitors have been developed for the treatment of HF. In congestive HF, PDE3 inhibitors in the short term induce a positive inotropic response and vasodilation (Movsesian et al, 2011). However, PDE3 inhibitors increase the mortality incidence of patients as a result of arrhythmias and sudden death (Packer et al, 1991). Although not fully elucidated, the mechanisms involved herein might implicate increased intracellular calcium concentrations due to chronically increased cAMP (Packer et al, 1991). In rodent models, pimobendan induced significant diastolic dysfunction with an increase in type I collagen deposition (Nakata et al, 2019). PDE3 inhibition might also be detrimental in HF through its effect on apoptosis. In vitro, PDE3A has been implicated in the activation of ICER (inducible cAMP early repressor), which is a transcriptional repressor of the antiapoptotic molecule Bcl-2. The inhibition of PDE3A leads to an increase in cAMP levels and consequent activation of PKA, which increases the ICER protein, resulting in cardiomyocyte apoptosis (Yan et al, 2007).

Conversely, transgenic mice overexpressing the Pde3a1 isoform specifically in the myocardium showed a cardioprotective effect in an ischemia and reperfusion model, with a reduction in the number of apoptotic cells and the size of the infarcted area in these transgenic animals. These beneficial effects were associated with a reduction in the cAMP/PKA/ICER signaling pathway and an increase in the Bcl-2 protein. These data suggest a therapeutic potential of PDE3A1 activation to prevent the deleterious effects of HF (Oikawa et al, 2013). Interestingly, activating mutations in PDE3, responsible for a rare disease characterized by the combination of brachydactyly and hypertension, confers cardioprotection despite the increase in afterload (Ercu et al, 2022). This latter observation supports the postulate reported earlier (Karam et al, 2020) that increasing rather than decreasing the activity of PDEs in cardiac tissue is beneficial. Despite the clinical limitations and unfavorable findings in rodent models, PDE3 inhibitors had positive effects on isoprenaline-induced myocardial injury in rats (Nakata et al, 2019) and showed favorable results on LV remodeling in mouse thoracic aortic constriction (TAC; Polidovitch et al, 2019). With *Pde3a* and *Pde3b* gene KO mice, PDE3A was found to be responsible for the beneficial milrinone effects in TAC (Polidovitch et al, 2019). Perhaps the adverse effects of PDE3 inhibition do not involve PDE3A, although this remains controversial (Movsesian, 2003).

In models of myocardial ischemia and reperfusion in dogs, a bolus injection of PDE3 inhibitor 30 minutes before coronary occlusion decreased the infarcted zone through the PKA/MAPK p38 pathway (Sanada et al, 2001; Sanada et al, 2004). This was likely due to PDE3B inhibition, as in vivo and Langendorff-perfused heart models of acute ischemia and reperfusion using *Pde3a*- and *Pde3b*-deficient mice, the size of the infarcted area was reduced only in the latter (Chung et al, 2015). Thus, despite the adverse effects of chronic PDE3 inhibitors in HF, their preischemia, single bolus use protects the myocardium against ischemic injuries (Sanada et al, 2001).

##### d PDE4

Numerous studies have shown the involvement of PDE4 in cardiac pathophysiology in animal models, suggesting the negative impact of its absence. Accordingly, the involvement of PDE4B has been shown in tachycardia, involving Ca_V_1.2 channel current amplitude elevation, and in HF of various etiologies, where PDE4B is decreased, and its overexpression attenuates HF (Abi-Gerges et al, 2009; Leroy et al, 2011; Mika et al, 2019; Karam et al, 2020). In addition to PDE4B, *Pde4d*-deficient mice showed an increase in ventricular tachycardia when subjected to physical training and developed cardiomyopathy with age (Lehnart et al, 2005). PDE4D resides in a close spatial relationship to the type 2 ryanodine receptor (RyR2), and its disappearance increases RyR2 phosphorylation by PKA, resulting in increased Ca^2+^ leakage from the SR prompting arrhythmias (Lehnart et al, 2005). PDE4D was shown to be decreased in the myocardium in humans with idiopathic cardiomyopathy (Richter et al, 2011a). PDE4D5 was shown to counteract hypertrophic events in neonatal cardiomyocytes (Berthouze-Duquesnes et al, 2013). In this study, PDE4D5 forms a complex with *β*-arrestin 2, and its disruption leads to a change from non-hypertrophic *β*_2_-adrenoceptor signaling to hypertrophic *β*_1_-adrenoceptor signaling involving Epac1.

PDE4 is also expressed in human atrial myocytes, where it contributes to cAMP hydrolysis by controlling Ca^2+^ influx through Ca_V_1.2 channels under basal conditions and under *β*-adrenoceptor stimulation (Molina et al, 2012). Inhibition of PDE4 in these atrial cells leads to an increase in the frequency of Ca^2+^ sparks and waves, leading to arrhythmias. In alignment with these findings, PDE4 activity has been shown to be reduced in patients with atrial fibrillation, a condition in which there is an alteration in the cAMP signaling pathway and Ca^2+^ dynamics (Molina et al, 2012). This highlights the involvement of this family of PDEs, not only at ventricular level but also in the human atrial myocardium, in the control of cAMP levels, both under basal conditions and under adrenergic stimulation, thus protecting against the development of arrhythmias.

##### e PDE8

The importance of PDE8 in cardiac pathophysiology has yet to be fully explored. In *Pde8a* isoform KO mouse *β*-adrenoceptor-stimulated Ca^2+^ transients, *I*_Ca,L_ and Ca^2+^ sparks in ventricular cardiomyocytes are increased (Patrucco et al, 2010). More recent work has demonstrated PDE8A and PDE8B in the human atrium, and the role of PDE8B in persistent atrial fibrillation. PDE8B is located in the plasma membrane in the atrial myocytes of humans with paroxystic atrial fibrillation, where it controls cAMP levels. Its expression is increased in these patients and reduces cAMP levels in the vicinity of the Ca_V_1.2 channel, leading to a reduction in *I*_Ca,L_ current (Grammatika Pavlidou et al, 2023). This is the first evidence of a role for PDE8 as a regulator of L-type voltage-gated Ca^2+^ channels in human atrial myocytes.

#### 3 PDE regulation of cardiac function through cGMP

##### a PDE5A and PDE9A

The cGMP-PDE PDE5A and PDE9A are expressed in the cardiomyocytes at low levels and localized, respectively, at Z-discs, and at T-tubular structures and mitochondria (Nagayama et al, 2008; Lee et al, 2015). PDE5 modulates NO-stimulated guanylyl cyclase-1 (GC-1)-derived cGMP, and PDE9 shapes NP-guanylyl cyclase A-derived cGMP signaling (Takimoto et al, 2005; Lee et al, 2015). The result of cGMP signaling in the heart as shaped by PDE5 is well studied and involves a number of downstream targets that affect hypertrophy, antioxidant defense, mitochondrial respiration, angiogenesis, and proteostasis, leading to protection against ischemic damage and hypertrophy, as carefully reviewed elsewhere (Samidurai et al, 2023). In intact hearts subjected to pathological pressure overload, both PDE5A and PDE9A inhibitions similarly reduced hypertrophy, and fibrosis and improved cardiac function. However, if animals were concomitantly administered with L-NAME to inhibit NO synthase, then only the PDE9A inhibitor conferred these effects. Removal of ovaries in females reduces estrogen-coupled NOS activation. When this was done, PDE5A blockade also became ineffective (Sasaki et al, 2014; Fukuma et al, 2020), whereas PDE9A inhibition still protected the myocardium (Mishra et al, 2021).

Oxidative stress differentially modifies the regulatory function of PDE5 versus PDE9. Oxidation of cGMP-dependent kinase-1*α* (cGK-1*α*, also known as PKG1*α*) at 2 cysteine residues in the homodimer *N*-terminus (C^42^-C^42^) alters the localization of cGK-1*α* to assume a more diffuse cytosolic pattern (Nakamura et al, 2015; Nakamura et al, 2018). Preventing this oxidation by expressing a C42S mutation in cGK-1*α* itself reduced the maladaptive response to pressure overload associated with the maintenance of the kinase to the sarcolemmal membrane ((Nakamura et al, 2015). However, the outer membrane is not where PDE5A colocalizes, and likely as a result, PDE5A inhibition was ineffective in mice harboring this same cGK1*α* C42S mutation (Nakamura et al, 2018), as was also the case for sGC activator (Nakamura et al, 2018). By contrast, cell studies indicate that this does not apply to PDE9A inhibition that remains effective in this setting (Dunkerly-Eyring et al, 2022). The therapeutic implication is that inhibiting PDE9A is more likely to be impactful over PDE5A to activate cGK1*α* under conditions of oxidative stress, a common feature in many heart diseases.

Both PDE5A and PDE9A protein expressions are increased in human HF (Pokreisz et al, 2009; Shan et al, 2012b; Lee et al, 2015; Besler et al, 2021), and their inhibition has been found protective in heart disease of various etiologies (Kamel et al, 2023). The common denominator herein is the activation of cGK-1. Inhibition of PDE5A suppresses NFAT signaling that, in turn, is coupled to phosphorylation of the transient receptor canonical channel types 3 and 6 (Koitabashi et al, 2010; Kiso et al, 2013), interaction with the regulator of G-coupled signaling protein types 2 and 4 to counter Gq-coupled agonist stimulation (eg, by angiotensin II; Takimoto et al, 2009; Nishida et al, 2010), phosphorylation of tuberin (TSC2) at S^1365^ (S^1364^ in humans) to suppress activation of mTORC1 complex signaling (Ranek et al, 2019), and of carboxy terminus heart shock cognate interacting protein (CHIP, also *Stub1*) at S^20^ (S^19^ in humans) to enhance protein quality control (Ranek et al, 2020). PDE9A inhibition achieves a number of similar downstream effects, although not as many have been tested to date.

PDE9A, but not PDE5A, localizes to mitochondria and modulates fatty acid oxidation (Mishra et al, 2021). PDE9A inhibition induced a thermogenic program in brown and white adipocytes (fat browning) both in vitro and in a model of severe, diet-induced obesity with and without pressure-load stress on the heart. Chronic PDE9A inhibition reduced fat mass with no change in lean mass, improved heart function, and reduced liver steatosis. These changes required activation of the transcriptional regulator PPAR*α*, a master controller of fat metabolism genes. Interestingly, PDE9A inhibition only caused these changes in males and ovariectomized females but did not affect intact females. The likely cause is that estrogen suppresses PPAR*α* transcriptional control over fat catabolism genes. The exact link between mitochondrial PDE9A, PPAR*α*, and fat metabolism remains to be determined.

Although many animal studies using PDE5A inhibition have shown benefits for various myocardial diseases, this has not been translated into the clinic (Borlaug et al, 2015; Cooper et al, 2022). Both positive and negative placebo-controlled trials are available, and recent meta-analyses further emphasize the contradicting findings regarding a potential application in coronary disease or heart failure (Cao et al, 2018; Samidurai et al, 2023; Soulaidopoulos et al, 2024). Reasons are uncertain, but PDE5A inhibitors are widely used to treat pulmonary hypertension (PH) and erectile dysfunction, but HF indications remain elusive. PDE9A inhibition has been studied in a sheep model of acute heart failure, both alone or in combination with a neprilysin inhibitor (to further enhance cGMP levels that PDE9A would in turn regulate), improving heart and renal function (Scott et al, 2019; Scott et al, 2023). Clinical trials of PDE9A inhibition in HF are ongoing, and whether the promising animal data ultimately translate to humans for this PDE remains to be seen.

##### b PDE10A

Expression of the dual substrate PDE, PDE10A (Chen et al, 2020), increases in mouse and human HF myocardium. PDE10A inhibitor TP-10 pathological hypertrophy, fibrosis, and chamber dysfunction in various pressure overload models. Mice with genetic deletion of *Pde10a* displayed reduced pathological responses and less mortality after pressure overload stress. Although cAMP and cGMP are both increased, their relative importance for PDE10 inhibition effects is unknown. Interestingly, inhibition of PDE10A ameliorated cardiac damage resulting from doxorubicin toxicity while simultaneously suppressing tumor growth in a breast cancer model (Chen et al, 2023). Thus, PDE10A is interesting in the new field of cardio-oncology.

### B Smooth muscle cells, endothelial cells, and PDEs

#### 1 Clinical and pathophysiological background of vascular disease

Cardiovascular diseases account for around 30% of disability and mortality in economically developed countries, representing the largest healthcare problem according to the World Health Organization (WHO; Pencina et al, 2019). Vascular aging and disease are one of the main drivers (Abdellatif et al, 2023). Vascular disease can be roughly divided into 3 categories of obstructive arterial disease, ie, atherosclerosis leading to infarction, nonobstructive vascular aging, and aneurysms. Due to improved prevention and treatment of infarctions, vascular aging has increased (Jouabadi et al, 2023), which has led to an increase in diastolic compared with systolic heart failure and dementia.

Vascular smooth muscle cells (VSMC) and endothelial cells (EC) are the main cell types involved in vascular homeostasis and disease. This involves the following functions: vascular tone and compliance regulation, angiogenesis, regulation of endothelial permeability, and blood coagulation. Pericytes, fibroblasts, adipocytes, and resident macrophages play an auxiliary role in these functions. VSMC are the engine of the artery wall as they contract and relax. Contraction of VSMC depends on cytoplasmic Ca^2+^ increase, followed by CaM-mediated myosin light chain kinase phosphorylation, and subsequent phosphorylation-induced actin-myosin fiber shortening. Dephosphorylation of actin-myosin is executed by myosin light chain phosphatase (MLCP) at low Ca^2+^ causing relaxation. Rho kinase activation supports contraction by Ser-phosphorylation-induced inhibition of MLCP, thereby increasing Ca^2+^ sensitivity of the VSMC. Herein, cAMP-PKA and cGMP-PKG signaling inhibit phosphorylation-induced MLCP deactivation and Rho kinase, respectively (see [Ito et al, 2022] for a detailed review). Furthermore, cAMP lowers Ca^2+^ through modulation of L-type calcium channel opening (Lincoln and Cornwell, 1991; Majed and Khalil, 2012). VSMC can also migrate, redifferentiate into myofibroblasts, or both, which leads to vessel hypertrophy and fibrosis. In the microvasculature, the VSMC are present as pericytes, which are in contact with the EC, regulating, for example, the blood-brain barrier.

EC are the regulators of the vessel wall (Deanfield et al, 2007). They form a smooth layer covering the luminal side of the blood vessel. They have antithrombotic and anti-inflammatory activities, suppress VMSC proliferation, and provide an important barrier function. EC also releases signaling factors to VSMC that change vascular tone and mediate angiogenesis through the process of sprouting, with tip and stalk cells guiding neovascularization. Tonus regulation and vascular remodeling are regulated by VSMC guided by stimuli derived from the circulation or neurotransmitters released by sympathetic neurons. VSMC are also regulated indirectly via the release of vasoactive signaling factors from EC that respond to circulating substances, flow changes, or hypoxia. Hypoxia is also the main stimulus for angiogenesis.

**Fig. 12:**
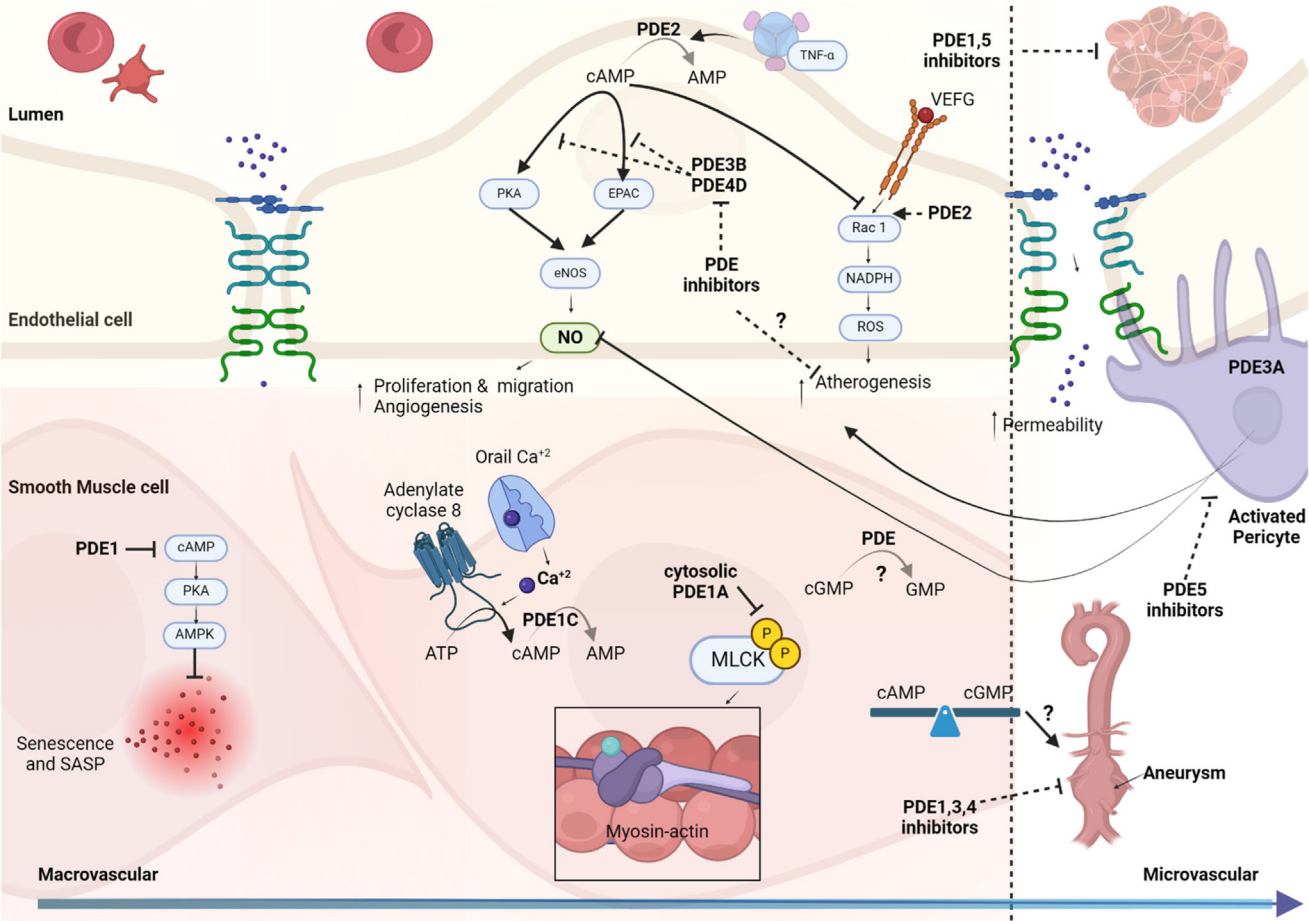
The importance of PDEs regulation in vasculature. VSMCs and ECs constitute the primary vascular cell types, with pericytes taking over the role of VSMCs in the microvasculature, alongside macrophages and other supporting cells. The regulatory influence of PDEs on cAMP has been implicated in endothelial permeability. Additionally, PDEs play a role in proliferation and cell migration, processes typically mediated by VEGF signaling and Rac1, leading to an increase in ROS produced by NADPH oxidase. PDE inhibitors have demonstrated promising effects in preventing aneurysm formation, a critical aspect of vascular remodeling disorders. Nevertheless, the precise mechanisms through which cyclic nucleotides balance each other to regulate arterial wall thickening/thinning remain unclear. Question marks indicate that the precise role of PDE in that specific pathway remains unknown. Created with BioRender.com.

Vascular disease is characterized by the disturbed communication between EC and VSMC, low-grade inflammation, endothelial permeability, and oxidative stress. Cellular senescence and its associated secretory phenotype (SASP) are believed to play an important role herein (Garrido et al, 2022; Clayton et al, 2023; Jouabadi et al, 2023). In summary, inflammation, senescence, apoptosis, increased EC permeability, decreased EC antithrombotic and angiogenic function, VSMC proliferation, migration, and fibrosis jointly contribute to vascular disease and related morbidities, such as HF and dementia. We discuss here the roles of cyclic nucleotide PDEs in these processes, focusing on VSMC and EC (Fig. 12).

#### 2 Role of PDEs in endothelial permeability and angiogenesis

##### a EC effects through cAMP

The elevation of cytosolic and membrane cAMP, respectively, decreases (cytosolic cAMP) and increases endothelial (membrane cAMP) permeability (Sayner et al, 2006). The required local gradient is created through the intervention of cAMP-PDEs (Feinstein et al, 2012). Various complementary studies (Seybold et al, 2005; Diebold et al, 2009; Surapisitchat et al, 2007) have shown that cGMP levels steer endothelial PDE2 and PDE3 to alternatively regulate permeability by cAMP. Low levels of cGMP or PDE3A inhibition, which maintains high cAMP levels, decrease thrombin-induced permeability. As cGMP increases, PDE2A activation bypasses PDE3A inhibition, thus switching from higher to lower cAMP levels, decreasing EC barrier function. Presumably this involves membrane cAMP levels. Endothelial PDE4 regulates microvascular permeability. Rolipram and roflumilast have been shown to attenuate vascular leakage in models of inflammation and sepsis (Schick and Schlegel, 2022). Further details describing downstream signaling and microdomains in endothelial permeability have been recently reviewed (Vina et al, 2021).

Important for angiogenesis, cAMP inhibits EC proliferation and migration (Netherton and Maurice, 2005). cAMP blocks proliferation through vascular endothelial growth factor (VEGF) signaling and a Rac1-mediated increase of NADPH-oxidase-produced reactive oxygen species (ROS; Sadek et al, 2020). Accordingly, PDE2, 3, and 4 inhibitions inhibit VEGF-induced proliferation and migration of cultured human EC, and angiogenesis in the chick chorioallantoic membrane model (Netherton and Maurice, 2005). Inhibition of these PDEs might also account for the antiangiogenic effect of curcumin (Abusnina et al, 2015). Downstream of VEGF, PDE2 inhibition involves the cyclin A—p27^kip1^, whereas combined PDE2 and 4 inhibition targets the cyclin D1—p21^waf1/cip1^ cell cycle pathway (Favot et al, 2004). In contrast, microvascular EC adhesion depends on cAMP-EPAC, and is regulated by PDE3B or PDE4D (Netherton et al, 2007). Furthermore, a role for pericyte PDE3A in cAMP regulation relevant to angiogenesis and permeability was proposed (Spiranec et al, 2018). Interestingly, PDE3 or 4 inhibition improved eNOS activity and NO-mediated tube formation in cultured human aortic EC through cAMP/PKA and phosphatidylinositol 3-kinase/Akt (Hashimoto et al, 2006). The result of the conflict with the antiangiogenic effects of cAMP and of PDE2, 3, and 4 inhibition is unclear. Unfortunately, in vivo experiments in mammal angiogenesis models with PDE2, 3, and 4 inhibitors are lacking. In addition, the role of other cAMP-selective PDE subtypes in the endothelium is unknown.

##### b EC effects through cGMP

cGMP is proangiogenic, and PDE1 and PDE5 emerge when cultured bovine EC passes from a quiescent to a proliferative phenotype (Keravis et al, 2000). Sildenafil increases in vitro tube formation and in vivo angiogenesis in a rat embolic stroke model, improving neurological recovery in the latter (Zhang et al, 2003; Zhang et al, 2005b). Sildenafil protects against renal microvascular degeneration in the mouse streptozotocin diabetes model (Pofi et al, 2017). Sildenafil and tadalafil were also reported to increase endothelial progenitor cell number in healthy humans and patients with erectile dysfunction (Bocchio et al, 2008; Foresta et al, 2009). The ongoing discussion about the identity and origin of endothelial progenitor cells and the general lack of clinical trials showing a positive effect of cell therapy make predictions of the therapeutic relevance of such observations challenging (Chambers et al, 2021).

The role of cGMP in endothelial permeability is unclear; an increase, decrease, or no effect has all been reported (Lakshminarayanan et al, 2000; Rentsendorj et al, 2008; Bohara et al, 2014; Yin et al, 2014; Bodiga et al, 2020). Differences in EC origin, permeability stimulus and measurement methods, and data interpretation may underlie the confounding results. With respect to interpretation, it is sometimes overlooked that NO increases permeability through catenins and not PKG (Marin et al, 2012). The role of cGMP-PDEs in regulating permeability has not been reported. Activation of pericytes leads to endothelial cell leakage, worsens NO-mediated vasodilation, and is antiangiogenic. This is prevented by PDE5 inhibitor sildenafil in a pericyte-EC coculture model (Majumder et al, 2007).

In summary, inhibition of PDE2, 3, and 4 might mitigate pathogenesis that involves increased endothelial permeability and angiogenesis. PDE5 inhibition seems applicable in conditions where angiogenesis is favorable. The specific role of PDE1 or other cGMP-PDE has not been evaluated.

#### 3 Role of PDEs in arterial wall remodeling and vascular tone

##### a Remodeling effects through cAMP

Antiproliferative effects in VSMC have been described for cAMP. Genetic knockout of *PDE1C* attenuates injury-induced neointima formation, involving cAMP/PKA and platelet-derived growth factor receptor *β* (Cai et al, 2015). Furthermore, PDE1C was shown to be involved in VSMC migration through a concerted interplay between adenylate cyclase 8 and orai1 Ca^2+^ channels (Brzezinska and Maurice, 2019; Brzezinska et al, 2021). PDE3 inhibition decreased neointima formation after arterial injury in rats (Indolfi et al, 1997; Ishizaka et al, 1999; Inoue et al, 2000) but had no effect on atherosclerosis in ApoE-KO mouse (Umebayashi et al, 2018). Further development as an antirestenosis therapy has not been pursued, possibly due to competition of cytostatin-eluting stents. Nevertheless, vascular aging-related intimal thickening and arterial stiffening might be considered as a future application.

Aneurysm formation is a disease with often dramatic consequences. Early investigations demonstrated that theophylline, a nonselective PDE inhibitor, induces aneurysms in chick embryos (Gilbert et al, 1977). In contrast, deletion of *Pde1c* and inhibition of PDE1, PDE3, or PDE4 with IC86340, cilostazol, or rolipram, respectively, were shown to be protective in abdominal aneurysm models (Zhang et al, 2011b; Umebayashi et al, 2018; Varona et al, 2021; Zhang et al, 2021; Gao et al, 2022). Cilostazol effects were associated with reduced inflammation, ROS levels, and matrix metalloproteinases 2 and 9 levels (Zhang et al, 2011b; Umebayashi et al, 2018). Of note, furthermore, it remains unclear if rolipram mediates its effects by inhibiting PDE4B in infiltrating inflammatory cells or PDE4D in VSMC (Varona et al, 2021; Gao et al, 2022). In summary, inhibition of cAMP-PDEs is a viable concept for the prevention of aneurysms.

##### b Remodeling effects through cGMP

Dual PDE1/PDE5 inhibition with SCH51866 had an antiplatelet and antihypertrophic effect in a hypertensive rat angioplasty model, whereas the selective PDE5 inhibitor, E4021, only decreased platelet adhesion (Vemulapalli et al, 1996). Zaprinast, which also targets PDE9 and 11, was reported to reduce neointima formation (Keswani et al, 2009). Vardenafil inhibited fibroblasts-to-myofibroblast transdifferentiation in a mouse model of focal segmental glomerulosclerosis (Hu et al, 2022), highlighting possibilities for antifibrotic treatment. Selective PDE1 inhibition has emerged as a target for diseases related to VSMC and adventitial fibroblasts (Zhou et al, 2010; Yan, 2015; Roks, 2022). Genetic knockout of *PDE1C* or inhibition of PDE1 with IC86340 attenuated injury-induced neointima formation. However, the mechanism of action of cGMP is unclear given that unlike cAMP (see above), PKG does not regulate platelet-derived growth factor receptor *β* (Cai et al, 2015). Similar to PDE3 inhibition, the development of PDE1 and PDE5 inhibitors as clinical drugs against in-stent restenosis night prove complicated, whereas attenuation of vascular aging might be a possible indication. Indeed, in a mouse model of accelerated aging, chronic sildenafil treatment significantly improved vasomotor function (Golshiri et al, 2020).

With respect to aneurysms, several cases of aortic dissection or subarachnoid hemorrhage have been reported in patients with erectile function taking sildenafil. Although this was attributable to an effect on VSMC, mechanistic evidence has not been reported (Edwin et al, 2009; Tiryakioglu et al, 2009; De-Giorgio et al, 2011). It is also unclear if the protective effect of PDE1 inhibition against arterial wall thinning described above for cAMP also involves cGMP (Zhang et al, 2021). Currently, it is not understood how both cyclic nucleotides interact to regulate arterial wall thickening (neointima formation) and thinning (aneurysms).

##### c Regulation of vascular tone by cAMP

PDE2 inhibition was shown to unmask or improve cAMP-mediated relaxation of the pulmonary artery and aorta of rats exposed to hypoxia to induce pulmonary hypertension (Bubb et al, 2014). PDE3A is known to be involved in brachydactyly short stature-hypertension, a rare inherited disease also known as Bilginturan syndrome (Bilginturan et al, 1973). Due to a diverse pallet of gain-of-function mutations in these patients, PDE3A activity in VSMC increases, causing hypertension (Ercu et al, 2023). Patients respond to most standard antihypertensive treatments, except for inhibitors of the renin-angiotensin system because this system is not activated. Left untreated, patients die from stroke at around the age of 50 years. Due to the detrimental cardiac effects, chronic PDE3 inhibition is not considered to be a logical alternative when standard antihypertensive treatment is effective.

Together with PDE10A, PDE3A is a target for papaverine (Li et al, 2023), a drug used to stop vasospasms in claudication. Based on their vasodilator and antithrombotic actions, the PDE3 inhibitors, cilostazol, and milrinone, are prescribed for intermittent claudication; their potential utility in vasospasm after cerebral hemorrhage is under clinical evaluation (Saber et al, 2018; Lakhal et al, 2021).

##### d Regulation of vascular tone by cGMP

PDE5 inhibition has been considered to improve clinical outcomes after stroke. A meta-analysis of oral sildenafil effects after subarachnoid hemorrhage suggested improved long-term anatomical and functional outcomes (Faropoulos et al, 2023). This is in contradiction with the alleged increased risk for stroke observed in patients with erectile dysfunction mentioned above. Explorative pharmacoepidemiological research might be necessary before initiating an intervention study.

In rats, under normoxic conditions, relaxations of the pulmonary artery induced by atrial natriuretic peptide (ANP) and NO are modified by the cAMP-PDE PDE2, whereas this was restricted to ANP in vessels harvested from hypoxic animals (Bubb et al, 2014). In the aorta, PDE2 inhibition increased NO-mediated relaxation, and this was also lost after exposure to hypoxia. Thus, the regulation of ANP/pGC/cGMP signaling by PDE2 present under healthy conditions appears to depend on the type of blood vessels or, perhaps, the hemodynamic conditions to which it is exposed. Hypoxia is known to promote vasoconstriction (Haynes et al, 1996), and apparently, this centers around the regulation of particulate guanylyl cyclase-generated cGMP. The NO/sGC/cGMP regulation by PDE2 is abolished by hypoxia. Almost complete loss of PDE2 transcripts and protein was observed after hypoxia, whereas paradoxically cytosolic PDE2 activity was still measurable (Bubb et al, 2014). Perhaps, a differential involvement of membrane-versus cytosolic-located PDE2 might explain this discrepancy. Microdomain regulation of PDE2 has been demonstrated in myocardial cells (Castro et al, 2006) and in stellate neurons of spontaneously hypertensive rats (Li et al, 2022) but remains to be elucidated in vascular cells.

The role of PDE1 and PDE5 in blood pressure and vasomotor function has been well investigated, has been comprehensively reviewed recently (Roks, 2022), and is summarized here. Although PDE1 is Ca^2+^/CaM-dependent, PDE5 is activated by PKA- and PKG-mediated phosphorylation. Activation of PDE5 is cGMP-dependent and suppressed by NO inhibition (Wyatt et al, 1998; Teixeira et al, 2006). In contrast to PDE5, PDE1 has cAMP-metabolizing activity (Francis et al, 2011). Thus, PDE1 and PDE5 are, respectively, active under contractile and relaxing conditions, and their (patho)physiological roles can therefore be expected to differ significantly.

Various genetic models implicated PDE1A in VSMC myosin-actin regulation, which involves myosin light chain kinase phosphorylation, and blood pressure (Nagel et al, 2006; Wang et al, 2017b). In contrast, *Pde1c* deletion has no blood pressure effect in young adult mice (Ahmad et al, 2015; Knight et al, 2016; Zhang et al, 2018b). PDE1 or PDE5 inhibition leads to modest blood pressure lowering in healthy animals and humans (Herrmann et al, 2000; Miller et al, 2009; Laursen et al, 2017; Hashimoto et al, 2018; Dey et al, 2020; Gilotra et al, 2021; Jüttner et al, 2022). The PDE1-selective inhibitors, Lu AF41228 and Lu AF58027, produced concentration-related relaxations of isolated rat mesenteric arteries in an NO- and cAMP-dependent manner (Laursen et al, 2017). The PDE1 inhibitor, lenrispodun, improved NO-mediated vasodilation and appears to be dominant over PDE5 inhibition in mouse aorta with aged VSMC (Ataei Ataabadi et al, 2021).

In physiological versus pathological conditions, expressions of PDE1 and PDE5 are differentially affected (Yan et al, 1996; Rybalkin et al, 1997; Rybalkin et al, 2002; Chen et al, 2018; Zhang et al, 2021). Recently, it was found that in the aorta of mice with aged VSMC, PDE1, rather than PDE5, impairs NO-cGMP-mediated signaling (Ataei Ataabadi et al, 2021). When NO-cGMP-PKG signaling is optimal, PDE5 represents a negative feedback mechanism to oppose relaxation. Conversely, under disease conditions where NO-cGMP signaling is lowered and Ca^2+^-induced constriction may be increased, PDE1 may further augment Ca^2+^ sensitivity by inactivating cGMP and cAMP. Thus, PDE1 appears to be the disease associated with PDE in VSMC (Roks, 2022). Indeed, in mouse models of accelerated aging, lenrispodun countered vascular aging more effectively than sildenafil (Golshiri et al, 2020; Golshiri et al, 2021b). In addition, PDE1, but not PDE5, appears to play a role in the regulation of vascular smooth muscle cell senescence (Bautista Niño et al, 2015; Zhang et al, 2021). PDE5 expression was reduced in in vitro aged VSMC (Wyatt et al, 1998), whereas PDE1 levels were increased in senescent human VSMC, aged mouse aorta, and VSMC of mouse aortic aneurysms (Bautista Niño et al, 2015; Zhang et al, 2021). In addition, PDE1 inhibition attenuated VSMC senescence (Bautista Niño et al, 2015; Zhang et al, 2021). This effect might be explained by the cAMP-mediated activation of sirtuin-1, which is important in nutrient sensing—energy metabolism, during PDE1 inhibition (Zhang et al, 2021). The role of cGMP still needs to be further interrogated especially given that soluble guanylyl cyclase activators have antisenescent effects in the aorta of mice with accelerated aging (Ataei Ataabadi et al, 2022). These observations for PDE1 versus PDE5 lead to a paradigm shift in their role in healthy and aged vascular tissue.

Of potential clinical relevance are epidemiological studies that have found single nucleotide polymorphisms (SNPs) in the *PDE1A* gene that are associated with diastolic blood pressure, mean arterial pressure, and common carotid intimamedia thickness (Tragante et al, 2014; Bautista Niño et al, 2015). *PDE1C* polymorphisms were not associated with vascular aging variables. It is unknown if this is due to the lack of functional mutations. Further discussion can be found elsewhere (Golshiri et al, 2019; Ataei Ataabadi et al, 2020; Roks, 2022).

PDE1 has also been proposed to play a role in nitrate tolerance during treatment of recurrent angina pectoris (Kim et al, 2001). However, these experiments employed vinpocetine, which is not selective for PDE1 (Dunkern and Hatzelmann, 2007). The role of PDE1 in vasodilation or blood pressure regulation, specifically in relation to cAMP, remains unclear. The distinct impact of PDE1 inhibition on blood pressure may be attributed to the opposing effects of vasorelaxation and positive inotropy (Gilotra et al, 2021). In conclusion, the balance of evidence suggests that inhibition of PDE1 or PDE5 may not be the optimal approach to treat hypertension. Nevertheless, the role of these PDEs in aging-related loss of vasodilation capacity warrants further inspection.

### C PDE inhibitions for pulmonary diseases

PDE inhibition is a prominent target in pulmonary disease. The groups of pulmonary diseases that are targeted for PDE intervention are diverse, involving different etiologies that implicate pulmonary, vascular, and inflammatory cell types, and the cardiovascular system. It comprises pulmonary hypertension, pulmonary fibrosis, chronic obstructive pulmonary disease (COPD), and asthma. These diseases will now be reviewed in this order.

#### 1 Pulmonary hypertension

PH is a debilitating and potentially fatal cardiovascular disorder (Schermuly et al, 2011) in which PDEs play a pivotal role (Ahmad et al, 2015). This section explores the diverse roles of PDEs in the pathogenesis and treatment of PH.

##### a Pathophysiology of PH

PH is a complex cardiovascular disorder characterized by elevated pulmonary arterial pressure (Schermuly et al, 2011). It increases afterload on the cardiac right ventricle (RV). If left untreated, it can result in right ventricular dysfunction and ultimately HF (Schermuly et al, 2011). The adaptation of the RV to the increased afterload is crucial for survival (Tello et al, 2023). PH can be classified into 5 main groups, each with distinct etiologies and pathophysiological mechanisms (Humbert et al, 2022).

##### b Subtypes and classification of PH

Pulmonary arterial hypertension (PAH; group 1) is the best-known subtype and is characterized by pathological changes in the small pulmonary arteries. Changes include vasoconstriction, vascular remodeling, and endothelial dysfunction (Schermuly et al, 2011). PAH is often idiopathic but can also be caused by connective tissue diseases, congenital heart diseases, or chemical poisoning (D'Alto and Mahadevan, 2012; Montani et al, 2013; Zanatta et al, 2019). Mutations in the *BMPR2* and other genes have been implicated in the development of heritable PAH (Evans et al, 2016; Morrell et al, 2019).

PH associated with left-sided heart diseases such as HF, valvular diseases, or LV dysfunction is classed as group 2 PH (Vachiery et al, 2019). Increased left atrial pressure is transmitted backward to the pulmonary circulation and can lead to pulmonary vascular abnormalities resulting in increased pulmonary vascular resistance (PVR; Rosenkranz et al, 2016).

PH associated with lung diseases (particularly COPD and interstitial lung diseases) is classed as group 3 PH (Gredic et al, 2021; Humbert et al, 2022). Hypoxia and inflammation are key factors contributing to vascular changes in this group (Ye et al, 2023).

Chronic thromboembolic PH (Group 4) is a unique form of PH caused by chronic pulmonary thromboembolism, where blood clots obstruct the pulmonary arteries (Pepke-Zaba et al, 2017; Ghofrani et al, 2021). Although normally addressed with pulmonary thromboendarterectomy, some patients require additional therapy or lung transplantation (Ghofrani et al, 2021).

PH with unclear and/or multifactorial mechanisms (group 5) encompasses a heterogeneous collection of PH conditions that result from hematological or systemic disorders or that have no clear cause (Lahm and Chakinala, 2013; Al-Qadi et al, 2020).

##### c Key mechanisms contributing to PH

One of the hallmark features of PH is increased pulmonary vasoconstriction and endothelial dysfunction (Evans et al, 2021; Kurakula et al, 2021; Hopkins and Stickland, 2023). Endothelial dysfunction disrupts the balance between vasodilators (eg, NO and prostacyclin) and vasoconstrictors (eg, endothelin-1, 5-hydroxytryptamine, and thromboxane; Huertas et al, 2018). Endothelin-1, in particular, plays a central role in promoting vasoconstriction and vascular remodeling in PH, both of which increase PVR (Chester and Yacoub, 2014).

Vascular remodeling in PH involves thickening of the pulmonary artery walls due to cellular proliferation and hypertrophy (Shimoda and Laurie, 2013; Leopold and Maron, 2016; Shimoda, 2020), involving all layers. This structural alteration narrows the vascular lumen. Endothelial dysfunction contributes to inflammation and thrombosis within the pulmonary arteries (Evans et al, 2021; Kurakula et al, 2021). Chronic inflammation is increasingly recognized as a key player in the pathogenesis of PH, especially in groups 3 and 5 (Frid et al, 2020; Yoo and Marin, 2022). Inflammatory cells, growth factors, cytokines, and signaling pathways such as the platelet-derived growth factor-*β* pathway are implicated in the vascular remodeling that increases PVR (Leopold and Maron, 2016; Shimoda, 2020; Liu et al, 2022; Wang et al, 2022).

Understanding the pathophysiology of PH is crucial for developing effective treatments. This section will further delve into the role of PDEs in the multiple pathways and mechanisms that contribute to and serve as therapeutic targets in PH.

##### d The importance of cAMP and cGMP in pulmonary vascular physiology and PH

In the context of PH, cAMP, and cGMP play a central role in regulating pulmonary vascular tone, cellular proliferation, and inflammation and are important downstream mediators of NO, natriuretic peptides, prostacyclin, and other hormones (Chen et al, 2013; Bobin et al, 2016). The cyclic nucleotides exert vasodilatory effects in the pulmonary circulation in the same manner as described in section Smooth Muscle Cells, Endothelial Cells, and PDEs for arteries in general (Lincoln and Cornwell, 1991; Ghofrani et al, 2002; Majed and Khalil, 2012; Bobin et al, 2016; Klinger and Kadowitz, 2017). Furthermore, inhibition of VSMC proliferation by cAMP is critical in vascular remodeling seen in PH (Growcott et al, 2006). cGMP inhibits platelet activation and aggregation, further preventing thrombosis in the pulmonary arteries, a common complication of PH (Wen et al, 2018; Degjoni et al, 2022). In PH, reduced levels of cAMP and cGMP due to increased PDE activity contribute to enhanced vasoconstriction, abnormal cellular proliferation, and inflammation, all of which are hallmark features of the disease (Muraki et al, 2019; Bubb et al, 2014). Hence, targeting PDEs to restore cyclic nucleotide levels represents a promising therapeutic approach in PH (Wilkins et al, 2008). The next section reviews the role of PDE subtypes and their potential as drug targets in PH.

#### 2 Role of PDEs in the pathophysiology and as drug targets in PH

##### a cAMP-PDEs in PH

###### i PDE3

PDE3 plays a pivotal role in vascular tone regulation and cell proliferation in PH via cAMP (Tilley and Maurice, 2002; Thelitz et al, 2004; Chen et al, 2009a). PDE3 counteracts vasodilation, contributing to vasoconstriction and increased PVR (Jeffery and Wanstall, 1998; Busch et al, 2010; Dillard et al, 2020). By blocking PDE3 activity, the levels of cAMP are preserved, leading to enhanced vasodilation and inhibition of VSMC proliferation (Murray et al, 2002; Dony et al, 2008). PDE3 inhibitors such as milrinone and cilostazol have shown promise in PH in experimental models and early phase clinical trials (Chen et al, 1997; James et al, 2016). Milrinone, originally developed as an inotropic agent for heart failure, has been repurposed for PH therapy (Bassler et al, 2006; James et al, 2016). However, its use is limited by its short half-life and the need for continuous intravenous infusion. Cilostazol is used primarily for its antiplatelet and vasodilatory effects in peripheral arterial disease (Manolis et al, 2022). Some studies have explored its potential in PH (Chang et al, 2008; Ito et al, 2021), but more research on its efficacy and safety in patients with PH is required.

###### ii PDE4

**Fig. 13:**
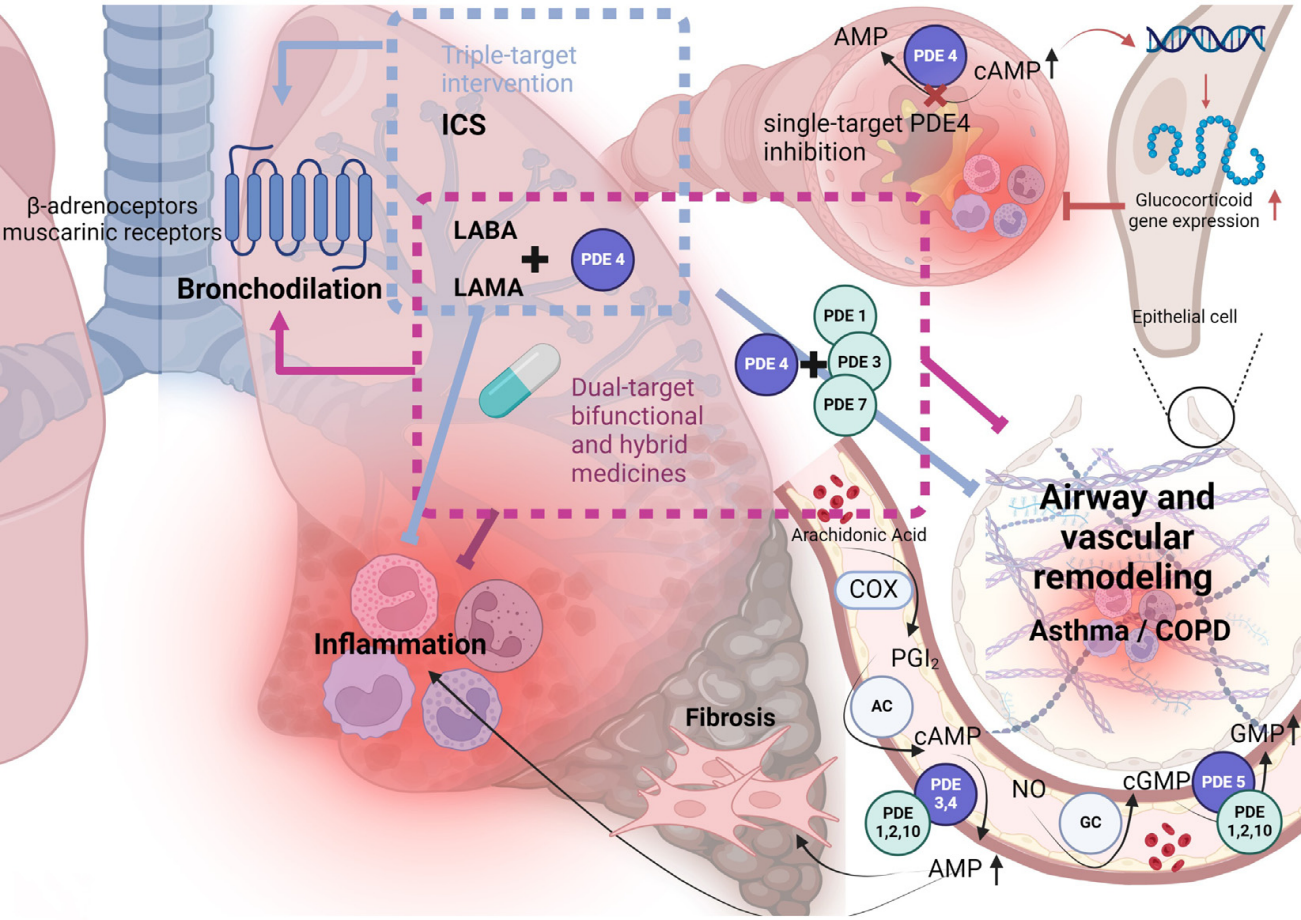
PDE-related treatment targets and modes of intervention in asthma, COPD and lung fibrosis. Interventions can be classified in single-target PDE4 inhibition (see section Asthma and COPD), and dual- and triple-target PDE4 combination therapy of PDE4 inhibition with other targets (see sections Hybrid Inhibitors for Asthma and COPD and Bifunctional Ligands for COPD). See body text for phase of pharmacotherapeutic development and potential reduction of side-effects of PDE4 inhibition exerted through the central nervous system. ICS, inhaled corticosteroids; LABA, long-acting *β*-adrenergic agonist; LAMA, long-acting muscarinic agonist. Created with BioRender.com.

PDE4 has been implicated in the pathophysiology of PH (Dony et al, 2008; Izikki et al, 2009). One of the distinguishing features of PH is chronic inflammation within the pulmonary vasculature (Pugliese et al, 2015). In this context, PDE4 plays a crucial role (Li et al, 2018). PDE4 isoforms are abundantly expressed in various immune cells, including macrophages and lymphocytes (Schick and Schlegel, 2022). Reduced cAMP levels result in heightened inflammation and immune cell activation (Raker et al, 2016), making PDE4 inhibitors as potential therapeutic agents in PH (Du et al, 2023). PDE4 inhibition increases intracellular cAMP levels in immune cells, leading to the downregulation of proinflammatory cytokines and reduced immune cell activation (Li et al, 2018). This can complement the effects of existing PH therapies that primarily focus on vasodilation and vascular remodeling (Meloche et al, 2013). PDE4 inhibitors have been explored in experimental PH (Izikki et al, 2009) which improves pulmonary hemodynamics, reduces vascular remodeling, and attenuates inflammation (De Franceschi et al, 2008; Izikki et al, 2009; Seimetz et al, 2015). PDE4 inhibitors require careful dosing to avoid gastrointestinal side effects (Kumar et al, 2013; Li et al, 2018). Therefore, challenges remain in optimizing dosing regimens, addressing potential side effects, and conducting larger-scale clinical trials to establish safety and efficacy in patients with PH (Fig. 13).

##### b cGMP-PDEs

###### i PDE5

PDE5 is particularly significant in the pathophysiology and treatment of PH (Barnes et al, 2019). PDE5 is highly expressed in the pulmonary vasculature, making it a central player in regulating cGMP levels in the lung (Wilkins et al, 2008; Tang et al, 2017). By hydrolyzing cGMP, PDE5 contributes to the vasoconstriction and vascular remodeling seen in PH (Chen et al, 2013; Tang et al, 2017). PDE5 is strongly upregulated in the medial layer of the lungs (Wharton et al, 2005) as well as in the hypertrophied RV of patients with PAH (Nagendran et al, 2007). Sildenafil (*Revatio*) and tadalafil (*Adcirca*) have proven their efficacy in placebo-controlled multicenter clinical trials (Ghofrani et al, 2004a; Galie et al, 2005; Galie et al, 2009; Montani et al, 2009; Barnes et al, 2019), improving exercise capacity, hemodynamics, and quality of life (Michelakis et al, 2003; Ghofrani et al, 2004b; Pepke-Zaba et al, 2008). Tadalafil, with its longer half-life (Forgue et al, 2006), offers the convenience of once-daily dosing. Some patients may develop resistance or suboptimal responses to PDE5 inhibitors over time (Unegbu et al, 2017; Barnes et al, 2019; Garcia et al, 2023), and research to enhance their effectiveness is ongoing. Combination therapy with other PH-targeted agents, such as prostacyclin analogs or endothelin receptor antagonists, is being investigated to address the multifactorial nature of PH and provide a more comprehensive treatment strategy (Humbert and Ghofrani, 2016). In this regard, it was shown experimentally that subthreshold doses of specific PDE inhibitors enhanced the pulmonary vasodilatory response to nebulized prostanoids (Schermuly et al, 1999; Schermuly et al, 2001). Clinically, PDE inhibition by sildenafil (Ghofrani et al, 2003) or the PDE3/4 dual-selective inhibitor tolafentrine (Ghofrani et al, 2002) amplified the pulmonary vasodilatory response to the inhaled prostacyclin analog iloprost and provided the basis for the implementation of combination therapy for PAH. This combination strategy aims to address vasoconstriction, vascular remodeling, inflammation, and maintenance of gas exchange (Ruopp and Cockrill, 2022). The combination of the PDE5 inhibitors sildenafil or tadalafil with endothelin receptor antagonists and/or prostacyclin analogs is the gold standard for the treatment of PAH and is included in the European PH treatment guidelines (Humbert et al, 2022).

Advantages of PDE5 inhibition in PH management include oral administration, relatively few side effects, and improvements in exercise capacity and quality of life (Buckley et al, 2010). Limitations include response variability (patient and PH subtype-dependent), the potential for drug interactions, and the need for careful monitoring of adverse effects, such as hypotension and visual disturbances (Rashid, 2005). Patient stratification based on underlying causes and pathophysiology is therefore very important (Humbert et al, 2022).

Ongoing research is exploring novel PDE5 inhibitors, alternative dosing regimens, and innovative drug delivery methods (Rashid et al, 2017). Furthermore, a deeper understanding of the heterogeneity of PH, clinical presentation, and the genetic factors influencing PDE5 responsiveness is guiding personalized treatment approaches (Savale et al, 2018; Wilkins, 2021). Also, challenges in combination therapy are being addressed (Lajoie et al, 2017; Dos Santos Fernandes et al, 2017; Burks et al, 2018), as well as interactions with drugs given for other indications (Dos Santos Fernandes et al, 2017; Lajoie et al, 2017).

##### c Dual-substrate PDEs in PH

###### i PDE1

PDE1 isoforms have garnered attention for their involvement in PH pathogenesis (Schermuly et al, 2007). Elevated PDE1 activity has been observed in PH in animal models and patients (Murray et al, 2007; Schermuly et al, 2007; Crosswhite and Sun, 2013). This heightened activity leads to reduced levels of both cAMP and cGMP, contributing to vasoconstriction, enhanced proliferation of VSMCs, and inflammation (Murray et al, 2007; Schermuly et al, 2007; Crosswhite and Sun, 2013). Importantly, the breakdown of the second messengers limits the efficacy of prostacyclin and NO. By blocking PDE1 activity, it is possible to increase intracellular levels of cAMP and cGMP, promoting vasodilation and inhibiting VSMC proliferation (Murray et al, 2007; Schermuly et al, 2007). In experimental models of PH PDE1 inhibitors improved pulmonary hemodynamics, reduced PVR, and attenuated vascular remodeling (Evgenov et al, 2006; Schermuly et al, 2007; Crosswhite and Sun, 2013). These findings support the idea that PDE1 inhibitors could be a valuable addition to the PH treatment arsenal (Fig. 13).

###### ii PDE2

Although less extensively studied than other PDE isoforms in PH, cellular and functional studies have shown that PDE2 inhibition elicits pulmonary vasodilation, prevents pulmonary vascular remodeling, and reduces right ventricular hypertrophy in experimental PH (Bubb et al, 2014). As PDE2 is also highly expressed in the failing heart (Bobin et al, 2016), further investigations into the mechanisms and effects of PDE2 dysregulation in the RV could offer valuable insights for the development of novel treatment strategies aimed at restoring cyclic nucleotide balance and ameliorating the vascular abnormalities associated with this debilitating disease.

###### iii PDE10

PDE10 is present in the pulmonary vasculature (Tian et al, 2011). In experimental models of PH, PDE10 inhibitors have been shown to increase cAMP levels, promote vasodilation, and reduce pulmonary vascular remodeling (Huang et al, 2019b; Tian et al, 2011). These findings suggest that PDE10 inhibition could offer a novel therapeutic approach for PH. The translation of preclinical findings into clinical applications is an ongoing process. The long-term safety and tolerability of PDE10 inhibitors need to be thoroughly evaluated in clinical trials, particularly considering the strong expression of PDE10 in the brain. Clinical trials evaluating the safety and efficacy of PDE10 inhibitors in schizophrenia are underway and may provide relevant information to guide clinical development in PH.

#### 3 Pulmonary fibrosis: epidemiology of pulmonary fibrosis

Progressive pulmonary fibrosis (PPF) and idiopathic pulmonary fibrosis (IPF) are interstitial lung diseases (ILDs) of known and unknown origin, respectively, and are characterized by progressive fibrosis of the pulmonary interstitium leading to an impairment of lung function and finally to death (Raghu et al, 2014; Cottin et al, 2019; Kolb and Vasakova, 2019; Raghu et al, 2022). Up to 40% of patients with ILDs may develop a progressing fibrotic phenotype, which is associated with high mortality, with median postdiagnosis survival in patients with IPF estimated at 2–5 years (Raghu et al, 2014). Progression of fibrosing ILD is reflected especially in a decline in pulmonary function, decrease in exercise capacity, deterioration in the quality of life, worsening of cough and dyspnea, acute exacerbations, and increase of morphologic abnormalities (Cottin et al, 2019; Kolb and Vasakova, 2019). In patients with IPF, a decline of forced vital capacity (FVC), the maximum amount of air one can forcibly exhale from the lungs after fully inhaling, is a well established predictor of mortality, and some blood biomarkers including the pneumyocyte type 2-regeneration marker KL-6, surfactant protein (SP)-D and extracellular matrix remodeling enzyme matrix metalloproteinase (MMP)-7 have been shown to be prognostic for disease progression (Karampitsakos et al, 2023). Currently, the only approved treatments to slow disease progression in IPF are nintedanib, a tyrosine kinase inhibitor, which is also indicated for PF-ILD, and pirfenidone, a pyridone with an unknown mechanism of action (Richeldi et al, 2018). However, the medical need for IPF and other progressive fibrosing ILDs remains high, with lung transplantation representing the only potentially curative treatment for IPF.

##### a Pathophysiology of pulmonary fibrosis

With respect to IPF pathophysiology, there has been a paradigm shift in recent years from a chronic inflammatory disorder to a primarily fibrotic disease. The current hypothesis of disease pathogenesis involves sustained alveolar epithelial microinjury, followed by a disordered repair, and wound healing response. This is characterized by uncontrolled activation of lung fibroblasts and differentiation to myofibroblasts, resulting in excessive extracellular matrix deposition and scarring of lung parenchyma, leading to loss of pulmonary function (Sgalla et al, 2018; Spagnolo et al, 2018). The wound-healing process includes an inflammatory phase, with the involvement of inflammatory cells and increased levels of cytokines and growth factors, creating a biochemical environment supporting chronic tissue remodeling. Nintedanib and pirfenidone have displayed antifibrotic and anti-inflammatory activity (Heukels et al, 2019) but slowed down the decline in FVC. Both compounds evoke significant side effects, leaving room for better-tolerated and efficacious medication. PDE4 inhibition offers this via anti-inflammatory and antifibrotic effects of cAMP (Richeldi et al, 2022). Increase in cGMP levels by inhibition of PDE5 is ineffective, eg, when sildenafil is given on top of nintedanib, in IPF (Kolb et al, 2018).

#### 4 Role of PDEs in pathophysiology and as drug targets in pulmonary fibrosis

##### a cAMP-PDEs: PDE4

In pulmonary fibrosis (PF), the possible application of PDE targeting is centered around PDE4. Very scarce evidence is available with respect to other PDEs, although PDE1, PDE5, and PDE9 have been mentioned as possible targets (Yildirim et al, 2010; Ren et al, 2017; Wu et al, 2020; Balaha et al, 2023). PDE4 has traditionally been implicated in the regulation of inflammation and the modulation of immune-competent cells, and data for the 3 selective pan-PDE4 inhibitors (active on PDE4A-D) currently marketed (roflumilast, apremilast, and crisaborole) support a beneficial role for PDE4 inhibition in inflammatory and/or autoimmune diseases (Hatzelmann et al, 2010; Sakkas et al, 2017; Li et al, 2018).

Important for PF, in bleomycin-induced PF in rats, rolipram initially was shown to inhibit fibrotic score, the content of fibrosis marker hydroxyproline, and of the inflammatory marker, serum TNF-*α* (Pan et al, 2009). A second early study in mice and rats showed that oral roflumilast was active both in preventive and therapeutic protocols (Cortijo et al, 2009). Cilomilast was shown to inhibit late-stage lung fibrosis and tended to reduce collagen content in the mouse bleomycin model (Udalov et al, 2010). In a murine model of lung fibrosis targeting type II alveolar epithelial cells, roflumilast lowered lung hydroxyproline content and mRNA expression of TNF-*α*, fibronectin (FN), and connecting tissue growth factor (CTGF; Sisson et al, 2018). Again, roflumilast was active both in a preventive and therapeutic regimen, and under the latter conditions, it appeared to be therapeutically equieffective with pirfenidone and nintedanib. Furthermore, in a mouse model of chronic graft-versus-host disease, lung fibrosis was attenuated by roflumilast (Kim et al, 2016).

PDE4 inhibitors might act indirectly via inhibition of proinflammatory cells (like alveolar macrophages) and their mediators and/or directly on fibrotic cell types. In human embryonal fibroblast models, PDE4 and prostaglandin E_2_ (PGE_2_), which increases cAMP, importantly interact: rolipram- and cilomilast-inhibited FN-induced chemotaxis and contraction of collagen gels, an effect that involved PGE_2_ (Kohyama et al, 2002). The inhibition of the fibroblast functions by cilomilast could be modulated by cytokines like IL-1*ß* or IL-4. In addition, TGF-*ß*1-stimulated FN release was inhibited by a PDE4 inhibitor, paralleled by stimulation of PGE_2_ release as a positive feedback mechanism (Togo et al, 2009). Roflumilast *N*-oxide, the active metabolite of roflumilast, in the presence of PGE_2_ was shown to inhibit intercellular adhesion molecule-1 and eotaxin release stimulated by TNF*α*, proliferation stimulated by basic fibroblast growth factor (bFGF) plus IL-1*ß*, as well as TGF*ß*1-induced *α*-SMA, CTGF, and FN mRNA expression in the presence of IL-1*ß* (Sabatini et al, 2010). In normal human lung fibroblasts, TGF-*β*-induced fibroblast-to-myofibroblast conversion assessed by *α*-SMA expression was shown to be inhibited by piclamilast in the presence of PGE_2_ (Dunkern et al, 2007). In subsequent papers, the same investigators showed the inhibition of IL-1*ß* plus bFGF-stimulated fibroblast proliferation by piclamilast and the importance of COX-2 and PGE_2_ (Selige et al, 2010). The importance of a cAMP trigger for the modulation of fibroblast functions by PDE4 inhibition was corroborated by the inhibition by roflumilast of TGF-*β*1-induced CTGF mRNA and *α*-SMA protein expression, and FN in the presence of the long-acting *β*_2_-adrenoceptor agonist, indacaterol (Tannheimer et al, 2012). Moreover, the inhibition by rolipram of another interesting aspect of fibrosis, epithelial-mesenchymal transition, was shown in the TGF-*ß*1-stimulated A549 human alveolar epithelial cell line (Kolosionek et al, 2009). Thus, a multitude of in vitro studies indicate that PDE4 inhibitors can directly inhibit various cAMP-dependent fibroblast functions. Upregulation of PDE4 activity by cytokines such as IL-1*β* may further enhance this role (Fig. 13).

There is also evidence for the role of isoform-selective PDE4 inhibition. By using PDE4 subtype-specific siRNA, the involvement of PDE4B and PDE4A in the attenuation of IL-1*ß* plus bFGF-stimulated fibroblast proliferation, as well as the involvement of PDE4B and PDE4D in TGF-*ß*-induced *α*-SMA expression, was shown (Selige et al, 2011). In most of the fibrosis-relevant cell types, like fibroblasts, macrophages, and epithelial cells, the PDE4B seems to have a more prominent role than other PDE4 subtypes (Hatzelmann et al, 2010), which was confirmed by the inhibition of cytokine release, proliferation, fibroblast-to-myofibroblast transition, and expression of extracellular matrix proteins by BI 1015550 (nerandomilast), a preferential PDE4B inhibitor with 9-fold selectivity for PDE4B vs PDE4D (Herrmann et al, 2022). In a phase II study in IPF patients, BI 1015550, although showing acceptable tolerability and safety, stabilized lung function in both patients with and without antifibrotic background therapy over 12 weeks and reduced disease-relevant blood biomarkers indicative of effects on the epithelium, fibrosis, and inflammation (Richeldi et al, 2022). BI 1015550 is currently in phase III clinical studies for IPF and PPF (NCT05321069 and NCT05321082).

#### 5 Asthma and COPD

Asthma is a pulmonary disease in which chronic inflammation plays a central role. Similarly, COPD is often associated with airway inflammation, although systemic manifestations are also commonplace. Although PDE inhibition is not a standard of clinical care for the treatment of either disorder, developments are ongoing that are largely centered around inhibitors of PDE4. Cigarette smoking is an important risk factor for asthma and COPD exacerbations and can increase the expression and function of certain PDE4 isoforms. For example, *PDE4D* mRNA abundance was significantly elevated in human airway smooth muscle (ASM) cells and precision-cut murine lung slices exposed acutely to cigarette smoke extract (Singh et al, 2009; Zuo et al, 2018). Moreover, an increase in *PDE4A4* mRNA and catalytic activity was detected in macrophages harvested from the bronchoalveolar lavage (BAL) fluid of smoking individuals with COPD relative to control subjects (Barber et al, 2004). The physiological consequences of enhanced PDE4 activity are ill-defined. However, in obstructive lung diseases, one might predict that a noxious insult that lowers cAMP would enhance pulmonary inflammation, which could be rectified with a PDE4 inhibitor (Milara et al, 2012; Zuo et al, 2018). These findings may have clinical relevance, given that a genome-wide association study of a cohort of Korean individuals identified a SNP in *PDE4D*, rs16878037, which was significantly associated with a susceptibility to nonemphysematous COPD (Yoon et al, 2014). Differences in the expression of transcripts that encode other PDEs including *PDE1A*, *PDE6A*, *PDE7A,* and *PDE11A* have also been detected in nasal and bronchial epithelial cells obtained from current smokers when compared with never smokers (Zuo et al, 2020). These changes have not been verified at the protein level and the (patho)physiological relevance is, therefore, unclear. Still, *Pde11a* has been linked, genetically, to inflammatory pulmonary conditions including asthma and symptomatic tuberculosis (Witwicka et al, 2007; Bazhin et al, 2010; DeWan et al, 2010; Oki et al, 2011; Zhu et al, 2019b). In the sections that follow, the potential therapeutic utility, limitations, and challenges of developing inhibitors of PDE4 and other PDE subtypes for asthma and COPD are reviewed.

##### a Asthma: epidemiology and pathophysiology

Asthma afflicts >350 million people globally and represents one of the most common, noncommunicable diseases with a prevalence that is predicted to increase to 450 million by 2025 (Vos, 2017). Mortality from asthma is low, but the burden it inflicts on society is considerable in terms of morbidity, quality of life, and associated economic costs (Dharmage et al, 2019; Reddel et al, 2019). Asthma is a heterogeneous disease with many endotypes that do not respond equally to current drug interventions (Wenzel, 2012). In ∼50% of cases, asthma has an allergic basis that is characterized by recurrent airway obstruction, airway hyper-responsiveness, airway inflammation, and airway remodeling (Woodruff et al, 2009).

Despite the heterogeneity of disease, treatment options for all patients with mild-to-moderate asthma are similar. The 2024 Global Initiative for Asthma treatment guidelines recommends that a combination of an inhaled corticosteroid (ICS) and the long-acting *β*_2_-adrenoceptor agonist (LABA), formoterol, is the “preferred” approach to provide as-needed relief of symptoms and maintenance control of the disease at all levels of severity (Reddel et al, 2019). Global Initiative for Asthma also advocates that a long-acting muscarinic receptor antagonist (LAMA) and/or biologicals be considered for the treatment of patients with severe disease in whom high-dose ICS/LABA combination therapy is suboptimal (Reddel et al, 2019). Regardless of these therapeutic approaches, many patients with severe asthma who suffer frequent exacerbations are still poorly controlled. In these difficult-to-treat cases, PDE inhibitors could prove to be beneficial as add-on therapies and remain in clinical development.

##### b COPD: epidemiology and pathophysiology

COPD is a leading cause of morbidity and mortality globally (Vogelmeier et al, 2017; Mirza et al, 2018; Reddel et al, 2019). According to the Global Initiative for Obstructive Lung Diseases (GOLD) guidelines, COPD is defined as “*a common preventable and treatable disease*” characterized by “*persistent airflow limitation that is usually progressive and associated with an enhanced chronic inflammatory response in the airways and the lung to noxious particles and gases*” (Vogelmeier et al, 2017; Mirza et al, 2018). Despite this definition, COPD is a generic term that describes a heterogeneity of endotypes (Miravitlles et al, 2013; Vestbo, 2014; Barnes, 2019) where chronic bronchitis, cough, idiopathic sputum production, airway wall thickening, mucus hypersecretion, and destruction of alveolar septa (ie, emphysema) are present to a greater or lesser extent (Barnes, 2004; Krzyzanowski et al, 2005; Barnes, 2008; McDonough et al, 2011); together, these pathologies contribute to the persistent, partially irreversible, and progressive decline in lung function that defines COPD (Postma and Timens, 2006; Hogg and Timens, 2009; van den Berge et al, 2011; Vogelmeier et al, 2017; Mirza et al, 2018). Due to high morbidity and mortality, COPD continues to impose a significant social and economic burden. Indeed, the number of deaths from COPD is projected to increase because of higher rates of cigarette smoking in the developing world and an aging global population in general (Vogelmeier et al, 2017; Mirza et al, 2018).

Treatment of COPD is, for the most part, restricted to bronchodilators. A LABA or a LAMA, each taken as a monotherapy or in combination, are recommended options to provide symptomatic relief (Vogelmeier et al, 2017; Mirza et al, 2018). In patients with more severe disease, anti-inflammatory therapy is often indicated with an ICS and/or an oral PDE4 inhibitor (Vogelmeier et al, 2017; Mirza et al, 2018). The utility of a PDE4 inhibitor is restricted to a subgroup of patients with COPD of a severe, bronchitic, frequent exacerbator phenotype who are not well controlled despite ICS and bronchodilator therapy (Briggs et al, 2010; Hurst et al, 2010; Giembycz and Maurice, 2014; Giembycz and Newton, 2014; Maurice et al, 2014; Vogelmeier et al, 2017; Mirza et al, 2018). Exacerbations of COPD are a major clinical concern because they are difficult to control, contribute significantly to the decline in lung function, and are the major cause of premature mortality (Mallia and Johnston, 2006; Kurai et al, 2013; Viniol and Vogelmeier, 2018). Hence, reducing the risk of a COPD exacerbation represents a primary therapeutic objective. Exacerbations of COPD are often precipitated by a bacterial and/or viral infection and are, thus, believed to have an inflammatory basis (Mallia and Johnston, 2006; Kurai et al, 2013; Ni et al, 2015; Viniol and Vogelmeier, 2018). This may explain why ICS and PDE4 inhibitors can be of benefit in subjects with COPD in whom airways inflammation is discernible.

##### c The utility of cAMP PDE inhibitors in asthma

PDE4 isoforms are expressed in most immune and structural cells of the airways and regulate many inflammatory processes (Torphy and Undem, 1991; Giembycz, 1992; Schudt et al, 1995; Torphy, 1998). This realization led to the proposal in the late 1980s that PDE4 might represent a novel target for treating inflammatory diseases. A primary indication was asthma, and a huge effort ensued to rigorously define PDE4 as a viable therapeutic target (Torphy and Undem, 1991; Giembycz, 1992; Schudt et al, 1995; Torphy, 1998). Despite initial optimism, the efficacy of PDE4 inhibitors as an asthma therapeutic has been uniformly disappointing, and most compounds selected for clinical development have been discontinued (Giembycz, 2008). The high rate of attrition is attributable to a low therapeutic ratio because of the inhibition of PDE4 in nontarget tissues with nausea and vomiting being the most severe, dose-limiting adverse effects. Thus, understanding the molecular basis of emesis became a priority. At that time, it was known that cAMP could enhance noradrenergic neuronal activity within the highly vascularized area postrema in the brainstem, which is linked, causally, to emesis (Carpenter et al, 1988). This was confirmed by delivering PDE4 inhibitors directly into the area postrema of ferrets by intracerebroventricular injection (Robichaud et al, 1999). Moreover, it was understood that PDE4 inhibitor-induced emesis is attenuated by the *α*_2_-adrenoceptor agonist, clonidine. Thus, emesis was assumed to be due to an increase of cAMP in noradrenergic neurons given that the *α*_2_-adrenoceptor is negatively coupled to adenylyl cyclase (Robichaud et al, 2001). Indeed, the tolerability of PDE4 inhibitors can be improved by limiting brain penetration (Aoki et al, 2001).

In the early 2000s, a popular hypothesis was that a specific PDE4 isoform regulated the emetic response. However, at that time, subtype-selective inhibitors had not been described. To overcome this limitation, a behavioral correlate of vomiting was developed in mice carrying targeted deletions of the genes encoding *Pde4b* and *Pde4d* (Robichaud et al, 2002). This approach was utilized because mice (and rodents in general) are anatomically constrained and cannot vomit; they are unable to relax their crural diaphragm or open their esophageal sphincter and appear to lack critical efferent pathways that drive the emetic reflex in higher mammals (Borison et al, 1981; Hanson, 2003; Horn et al, 2013). The murine model of emesis relies on the ability of *α*_2_-adrenoceptor agonists to promote anesthesia by reducing the cAMP content in noradrenergic fibers within the brainstem, which can be attenuated by PDE4 inhibitors (Giembycz, 2002; Robichaud et al, 2002). Robichaud et al (2002) found that the duration of *α*_2_-adrenoceptor-mediated anesthesia was significantly attenuated in mice lacking *Pde4d* but not *Pde4b* implicating a Pde4d isoform(s) in the emetic response. The presence of *PDE4D/Pde4d* within various brain regions of several species was consistent with this idea (Cherry and Davis, 1999; Takahashi et al, 1999; Pérez-Torres et al, 2000; Lamontagne et al, 2001). However, *PDE4B/Pde4b* has also been detected in many of these same brain regions (Pérez-Torres et al, 2000), and inhibitors of PDE4B, which are >80-fold selective over PDE4D, do not display a superior therapeutic index (Naganuma et al, 2009; Suzuki et al, 2013). Moreover, so-called, negative allosteric PDE4D inhibitors have been described, which *preferentially* partition into the brain and, therefore, will reach the area postrema. These compounds, of which D-159687 and zatolmilast are examples, display a unique mechanism of action in that they block cAMP hydrolysis by modifying the dimeric structure of PDE4D rather than by simply competing with the substrate at the catalytic site (Burgin et al, 2010; Houslay and Adams, 2010; Gurney et al, 2011). Based on the results obtained in *Pde4d* knockout mice, these compounds should promote emesis. However, paradoxically, they have significantly reduced emetic liability (Burgin et al, 2010; Gurney et al, 2011; Zhang et al, 2017b). Thus, the assumption that PDE4 inhibitors with weak activity against the 4D isoenzyme should be less emetic is likely misplaced. Indeed, several companies including Tetra Therapeutics (a subsidiary of Shionogi & Co) are purposefully developing selective PDE4D inhibitors (eg, zatolmilast) with Fragile X syndrome and AD being primary indications. Clinical trials of these compounds are ongoing (eg, NCT05367960 and NCT03817684), and it will be instructive, from the perspective of developing new PDE4 inhibitors for asthma and COPD, if the improved therapeutic ratios reported in animal models translate into improved safety and tolerability in humans.

##### d The utility of cAMP PDE inhibitors in COPD

The abject failure of PDE4 inhibitors as an asthma therapy prompted the pharmaceutical industry to repurpose this drug class for other airway diseases, in particular, COPD. Cilomilast (*aka Ariflo*, SB-207499), developed by GlaxoSmithKline (GSK), was the first PDE4 inhibitor to progress to phase III clinical trials (Giembycz, 2001; 2006; Schachter, 2006; Kroegel and Foerster, 2007; Rennard et al, 2008). The decision to develop cilomilast was based on a conceptually robust hypothesis, abundant preclinical data, and the encouraging results of phase II clinical studies (Torphy et al, 1999). However, the results of the phase III development program were disappointing and did not meet the expectations of the phase II studies (Giembycz, 2001, 2006; Schachter, 2006; Kroegel and Foerster, 2007; Rennard et al, 2008). Like the asthma trials, dose-limiting adverse events remained a major cause for concern due, in part, to the interaction of cilomilast with PDE4 in “off-target” tissues. Despite these unremarkable data, the FDA, in October 2003, issued an approval letter to GSK for the use of cilomilast in the “*maintenance of lung function in COPD patients poorly responsive to salbutamol*” (Clinical_Trials_Arena, 2003). However, this was conditional on the outcome of further efficacy and tolerability studies, which were to focus on gastrointestinal events of concern, the sustainability of clinical benefits, and whether the difference in lung function between the cilomilast- and placebo-treated subjects improved further in long-term dosing studies. These additional trials were, presumably, unsuccessful since the development of cilomilast was discontinued in 2007.

Other PDE4 inhibitors originally developed for asthma have also been repurposed for COPD. In particular, the results of several large, international, multicenter, randomized, placebo-controlled trials led the European Medicines Agency, in April 2010, to approve the use of roflumilast (*aka Daxas*, *Daliresp*, Byk 2869) for the “*maintenance treatment of severe COPD associated with chronic bronchitis in adult patients with a history of frequent exacerbations as add-on to bronchodilator treatment*” (Rabe et al, 2005; Calverley et al, 2007; Calverley et al, 2009; Fabbri et al, 2009; Giembycz and Field, 2010; Gross et al, 2010; Wedzicha et al, 2016). Given orally, roflumilast (500 *μ*g o.d.) significantly improved lung function and reduced the frequency of exacerbations. Notably, these beneficial effects were more pronounced in patients with severe, bronchitic disease suggesting that the primary activity of roflumilast was to suppress inflammation (Miravitlles et al, 2013; Giembycz and Newton, 2014). Nevertheless, the most common adverse effects were gastrointestinal discomfort and headache (presumably due to cerebrovascular vasodilation; Rabe et al, 2005; Calverley et al, 2007; Calverley et al, 2009; Fabbri et al, 2009), which were similar to those produced by all other PDE4 inhibitors that had been evaluated clinically. Currently, roflumilast is one of only 2 PDE4 inhibitors that has been approved for COPD and offers physicians an add-on treatment option for patients with more severe disease in whom traditional bronchodilator and glucocorticoid therapies are suboptimal.

Despite the emetic liability of PDE4 inhibitors, interest in these compounds as therapeutics for respiratory diseases continues with tanimilast (*aka* CHF-6001) being the most advanced candidate in clinical development. Tanimilast is a highly potent, subnanomolar inhibitor of PDE4 that does not discriminate between PDE4 isoforms (Armani et al, 2014). In cell-based assays and preclinical models of airway inflammation, it displays pleiotropic anti-inflammatory activity with limited emetic liability (Fioni et al, 2018; Facchinetti et al, 2021; Schioppa et al, 2022). For example, tanimilast (1 *μ*mol/kg i.t.) inhibited allergen-induced eosinophilia in rats by >90% without producing nausea-like behavior in conscious ferrets, which likely reflects low systemic exposure and limited ability to cross the blood-brain barrier (Villetti et al, 2015). In contrast, GSK 256066, another highly potent PDE4 inhibitor (Tralau-Stewart et al, 2011) that was used as a comparator, was equally effective at blunting pulmonary eosinophil recruitment yet produced clear behavioral signs of nausea (Villetti et al, 2015). Unlike roflumilast, which was formulated for oral dosing, tanimilast has been optimized for inhaled delivery as a dry powder to limit systemic side effects. Studies in subjects with COPD have shown that at steady state (after 800 *μ*g or 1600 *μ*g inhaled twice a day for 32 days), the concentration of tanimilast in sputum was approximately 2000-fold higher than in plasma indicating high pulmonary retention and low systemic exposure (Singh et al, 2019). Moreover, in the PIONEER (new **P**hosphodiesterase **I**nhibitor with **O**ptimal anti-i**N**flammatory **E**ffect dos**E R**esponse in COPD patients) phase IIb trial, tanimilast was well tolerated with a similar incidence of adverse events across 4 increasing doubling doses (Singh et al, 2020a).

On the basis of successful safety, tolerability, and preliminary efficacy studies, 2 52-week phase III clinical trials (PILASTER [a new inhaled **P**hosphodiesterase **I**nhibitor Eva**L**uated on moderate/severe ex**A**cerbation**S** on top of maintenance **T**riple th**ER**apy in COPD Patients] and PILLAR [a new inhaled **P**hosphodiesterase **I**nhibitor given on top of maintenance trip**L**e therapy in COPD Patients eva**L**uation on moderate/severe ex**A**ce**R**bations]) have been initiated to assess if tanimilast can reduce the frequency of exacerbations in a population of patients with severe, bronchitic COPD who are still symptomatic despite treatment with ICS/LABA/LAMA combination therapy (Facchinetti et al, 2021). Indeed, there remains a high unmet clinical need to identify interventions that can better control this relatively unresponsive COPD endotype.

##### e Scientific rationale for “adding-on” a PDE4 inhibitor to combination therapy

Glucocorticoid monotherapy is poorly effective in COPD and can be contraindicated due to an increased risk of pneumonia and tuberculosis (Ernst et al, 2007; Brassard et al, 2011). However, clinical trials data indicate that ICS-containing combination therapy is more effective than a bronchodilator in reducing COPD exacerbations (Ding et al, 2022). This could suggest the utility of adding-on a PDE4 inhibitor in the subpopulation of individuals with severe, bronchitic COPD in whom symptoms persist despite treatment with ICS/LABA/LAMA triple therapy. Data to support this hypothesis and rationalize the PILASTER and PILLAR trials can be derived from *post hoc* analyses of data from the earlier phase III roflumilast development program. Thus, roflumilast reduced the rate of exacerbations in individuals with severe COPD who were taking an ICS concurrently, whereas no such benefit was derived if ICS were excluded (Rennard et al, 2011). Lung function in COPD patients of the bronchitic phenotype was also improved by roflumilast, regardless of co-existing emphysema, and this was greater if they had received concomitant ICS rather than placebo (Rennard et al, 2011). Collectively, these data imply that an ICS and roflumilast in combination have superior therapeutic activity than either drug alone.

Glucocorticoids suppress inflammation by modulating the expression of hundreds of genes including those that encode cytokines, chemokines, and growth factors of which gene induction (*aka trans*activation) is a major mechanism (Newton, 2014). Moreover, there is compelling evidence that cAMP-elevating agents can interact with glucocorticoids to further modulate the genomic response (Giembycz and Maurice, 2014; Giembycz and Newton, 2011, 2014, 2015). This molecular interaction, first described in the early 1990s (Rangarajan et al, 1992), may help explain how adding-on a LABA and a PDE4 inhibitor to an ICS could reduce inflammation and improve lung function in individuals with asthma and COPD. Indeed, an increase in cAMP can augment glucocorticoid-induced gene expression changes in several cell types including the airway epithelium (Giembycz et al, 2008; Kaur et al, 2008; Wilson et al, 2009; Greer et al, 2013; Moodley et al, 2013; BinMahfouz et al, 2015; Joshi et al, 2015; Newton and Giembycz, 2016; Rider et al, 2018; Reddy et al, 2020; Turner et al, 2020; Mostafa et al, 2021). In many cases, the cAMP-elevating agent *per se* is inert but interacts with the glucocorticoid in a positive, cooperative fashion to enhance gene transcription. This implies that a LABA or PDE4 inhibitor is “steroid-sparing” because the glucocorticoid can now produce a given level of gene induction at a significantly lower concentration (Kaur et al, 2008; Joshi et al, 2015). Moreover, in airway epithelial cells, roflumilast potentiated the ability of the LABA, formoterol, to enhance the expression of a panel of glucocorticoid-inducible genes that may have anti-inflammatory activity in COPD (Moodley et al, 2013). Thus, agents that increase the cAMP content in target tissues may exert therapeutic activity in obstructive lung diseases beyond bronchodilation (Fig. 13).

A PDE4 inhibitor should also potentiate *β*_2_-adrenoceptor-mediated cAMP formation in the airways. This interaction could be particularly relevant in proinflammatory or immune cells where *β*_2_-adrenoceptors are expressed in low abundance or are poorly coupled to adenylyl cyclase. In this situation, a cell type that responds weakly to a LABA (eg, an eosinophil; Rabe et al, 1993; Muñoz et al, 1995) could be sensitized by a PDE4 inhibitor allowing a cAMP signal to be generated of sufficient magnitude to enhance glucocorticoid-induced gene expression (Fig. 13). Collectively, these results provide a mechanistic basis for the clinical efficacy of ICS/LABA/PDE4 inhibitor triple combination therapy on COPD exacerbations reported throughout the roflumilast phase III clinical development program (Rennard et al, 2011). By extension, adding-on a PDE4 inhibitor to ICS/LABA combination therapy in individuals with difficult-to-treat asthma might also afford additional benefit (Fig. 13).

It is noteworthy that *β*_2_-adrenoceptor agonists and other cAMP-elevating agents can also increase the expression of various PDE4 isoforms. Typically, these are transcriptional responses and occur in airway immune and structural cells alike including ASM (Le Jeune et al, 2002; Hu et al, 2008), monocytes (Torphy et al, 1992; Torphy et al, 1995; Manning et al, 1996), T-lymphocytes (Erdogan and Houslay, 1997; Seybold et al, 1998), neutrophils (Ortiz et al, 2000), and EC (Zhu et al, 2004). The implications of these gene expression changes may be significant because SABAs and LABAs, which are consumed by individuals with asthma and COPD on a long-term basis, could attenuate signaling mediated by all GPCRs that stimulate adenylyl cyclase (Giembycz, 1996). In this context, the concurrent use of a PDE4 inhibitor can be rationalized because heterologous GPCR desensitization could be mitigated.

#### 6 Hybrid inhibitors for asthma and COPD

Polypharmacology is a branch of pharmacology that is dedicated to understanding the mechanism of action of compounds that interact with more than 1 molecular target in a disease network (Jalencas and Mestres, 2013). This discipline addresses the likelihood that improved clinical outcomes can be realized over the traditional “1 drug, 1 target” concept of therapeutics (Morphy and Rankovic, 2005). Several polypharmacological approaches are possible including the administration of: (1) 2 or more drugs separately or together in a single formulation; (2) 2 or more prodrugs formulated as a single chemical entity that is released at the desired site of action by enzymatic cleavage; and (3) a single chemical entity that interacts with 2 or more targets, simultaneously (Morphy and Rankovic, 2005). Compounds in this latter category include bifunctional ligands (see last section The Role of PDEs in Specific Immune Cell Types and Fig. 13) and hybrid PDE inhibitors that contain a single “promiscuous” pharmacophore that blocks the catalytic sites of 2 or more PDE isoforms. Of the 11 PDE families described, the simultaneous inhibition of PDE4 and either PDE1, PDE3, or PDE7 may provide the means to further enhance clinical efficacy (Giembycz, 2005b; Giembycz and Newton, 2011; Zuo et al, 2019). Indeed, as mentioned above, the discovery of multicomponent, syncretic drugs provides a theoretical means to treat various asthma and COPD endotypes, given that additive and/or synergistic outcomes can be produced when multiple PDEs are inhibited concurrently (Keith et al, 2005).

*Inhibitors of PDE4 and PDE1*. Airway remodeling is a characteristic feature of obstructive lung diseases (Hossain and Heard, 1970; Jeffery, 2001; Lazaar and Panettieri, 2003; Wenzel, 2003; Aoshiba and Nagai, 2004; Hogg, 2004; Hogg et al, 2004). In asthma, several processes can change the architecture of the respiratory tract including mucus gland hyperplasia, supepithelial deposition of collagens, mucosal revascularization, and an increase in ASM mass (Jeffery, 2001). Similarly, airway remodeling in COPD involves connective tissue deposition in the subepithelial and adventitial compartments (Dunnill et al, 1969; Hogg et al, 2004) and an increase in the density of ASM in the bronchioles (Hossain and Heard, 1970; Jeffery, 2001; Aoshiba and Nagai, 2004; Hogg et al, 2004). In both diseases, the remodeling process thickens and increases the volume of the respiratory tract wall, which contributes to airway hyper-responsiveness (Wiggs et al, 1990; Hogg, 1996; Postma and Kerstjens, 1998; Martin et al, 2000).

PDE1 is highly expressed in vascular smooth muscle where it has been implicated in the control of proliferation (see section Smooth Muscle Cells, Endothelial Cells, and PDEs). PDE1 is also highly expressed in ASM (Giembycz and Barnes, 1991; Torphy et al, 1993) where it may regulate the same function. On this basis, 1 might speculate that a dual inhibitor of PDE1 and PDE4 could retard remodeling and, at the same time, suppress inflammation. Data to support this idea include the ability of the PDE1/PDE4 inhibitor, KF-19514, to suppress inflammation and airway remodeling in a murine model of chronic asthma (Manabe et al, 1997; Fujimura et al, 1998; Kita et al, 2009; Manabe et al, 2000). However, a review of the literature suggests that this potential therapeutic opportunity has not gained traction.

*Inhibitors of PDE4 and PDE3.* PDE3 inhibitors are effective bronchodilators in humans (Leeman et al, 1987; Brunnée et al, 1992; Fujimura et al, 1995; Fujimura et al, 1997; Bardin et al, 1998; Myou et al, 1999; Myou et al, 2003; Singh et al, 2020b). This property led to the theory that compounds that block PDE3 and PDE4 at a similar dose could have a polypharmacological advantage over a selective PDE4 inhibitor by producing both ASM relaxation (PDE3-dependent) and anti-inflammatory activity (PDE4-dependent). In addition, many proinflammatory and immune cells also express PDE3 (Torphy, 1998; Banner and Press, 2009), and in many cases, the anti-inflammatory effects of concurrent inhibition of PDE3 and PDE4 are superior to those of PDE4 alone. For example, in vitro studies have shown that although PDE3 inhibitors have little or no effect on T-cell proliferation or on IL-2 generation, they enhance the repressive effect of a PDE4 inhibitor (Robicsek et al, 1991; Giembycz et al, 1996). Similar data have been reported for the inhibition of proinflammatory responses in human alveolar macrophages (Schudt et al, 1995), monocyte-derived dendritic cells (Gantner et al, 1999), airway epithelial cells (Wright et al, 1998), human lung fibroblasts (Selige et al, 2010), and human lung microvascular EC (Blease et al, 1998). It is noteworthy that evidence garnered from mouse models of asthma indicates that inhibition of Pde3a and Pde3b can abrogate several key hallmarks of the disease. In particular, pulmonary eosinophil, neutrophil, T-lymphocyte, dendritic cell, mast cell, and macrophage recruitment were suppressed implying that PDE3 per se may regulate previously unappreciated aspects of the allergic inflammatory response (Beute et al, 2018; Beute et al, 2020).

Based upon encouraging preclinical data, several hybrid PDE3/PDE4 inhibitors were developed and evaluated in humans including zardaverine, benzafentrine, tolafentrine, and pumafentrine but all were discontinued because of lack of efficacy, a poor adverse effect profile, or limited duration of action (Banner and Press, 2009). Nevertheless, interest in the PDE3/PDE4 inhibitor concept has endured with at least 2 compounds currently in clinical development for asthma and/or COPD: ensifentrine (*aka* RPL 554, *Ohtuvayre*) and, what appears to be a structurally related compound, TQC-3721(Yang et al, 2023). Indeed, the FDA recently approved ensifentrine for the maintenance treatment of adult patients with COPD (Kariya, 2024).

Ensifentrine is a well tolerated, long-acting inhaled bronchodilator derived from the PDE3 inhibitor, trequinsin (Boswell-Smith et al, 2006; Calzetta et al, 2013; Donohue et al, 2023). In 2023, ensifentrine progressed to phase III clinical evaluation, and the results of the 2 ENHANCE (**E**nsifentrine as a **N**ovel in**HA**led **N**ebulized **C**OPD th**E**rapy) trials were recently reported (Anzueto et al, 2023). In each study, >750 participants were enrolled with moderate-to-severe COPD and randomized to receive either placebo or ensifentrine (3 mg twice a day for 24 weeks). In both trials, FEV_1_ was the primary outcome measure and significantly improved with treatment relative to placebo. Exacerbation rates were also reduced by 40% in the active treatment groups (Anzueto et al, 2023). However, it is unclear if this was due to the inhibition of PDE4 (Singh, 2023) as there is no conclusive evidence that ensifentrine has anti-inflammatory activity (Singh, 2023). In one of the initial exploratory studies, a single dose of ensifentrine (0.018 mg/kg), given by inhalation to healthy men, inhibited the accumulation of neutrophils in sputum in response to lipopolysaccharide (LPS). Although this finding may implicate PDE4 (Franciosi et al, 2013), the relationship between pulmonary neutrophilia and the development of an exacerbation is moot.

Ensifentrine is, typically, referred to as a hybrid PDE3/PDE4 inhibitor as we have done in this review. However, this is a misrepresentation. Ensifentrine is >3440× more potent against PDE3 than PDE4 (Boswell-Smith et al, 2006), which is comparable to, or even greater than, the selectivity of compounds that are classified as selective PDE3 inhibitors including cilostazol, cilostamide, and milrinone (Sudo et al, 2000). This implies that at the inhaled doses of ensifentrine used in human subjects, inhibition of PDE3 will predominate. How, then, a single dose ensifentrine (0.018 mg/kg) attenuated LPS-induced pulmonary leukocyte recruitment becomes an important question. A plausible explanation is that the local concentration of ensifentrine at target cells after inhalation exceeds that required to abolish PDE3 activity (and, therefore, is supra-maximal for bronchodilation). Using the technique of bronchosorption (Leaker et al, 2015), the epithelial surface liquid (ESL) can be sampled via a catheter inserted through the working channel of a bronchoscope. It has been estimated that the concentration of the LABA, salmeterol, in the ESL of healthy subjects 1 h after inhalation of a 50 *μ*g dose was ∼80 nM (Sadiq et al, 2021). Assuming remotely similar pulmonary pharmacokinetics, the concentration of ensifentrine in ESL after inhalation of a 3 mg dose (used in the ENHANCE trial) could be ∼5 *μ*M. Indeed, both compounds have comparable molecular weights (∼450 Da) and clog D values (∼1.9 at pH = 7; calculated using ACD/Labs software) that presumably lead to high lung retention and low systemic exposure (Bäckström et al, 2016; Zuiker, 2016; Sadiq et al, 2021). Thus, inhalation of a 3 mg dose of ensifentrine could be sufficient to inhibit PDE4 in airway epithelia and inflammatory cells in BAL fluid by >70%. Indeed, the IC_50_ of ensifentrine for suppressing cytokine release (eg, GM-CSF, MCP-1, TNF*α*) from human airway epithelial cells and monocytes is in the low micromolar range (Boswell-Smith et al, 2006; Turner et al, 2020). Likewise, the threshold concentration for ensifentrine to increase *global* cAMP in human airway epithelial cells is reported to be ∼1 *μ*M (Turner et al, 2020).

In vitro studies have found that ensifentrine relaxed ACh-contracted human ASM (EC_50_ ∼10 *μ*M) and inhibited PDE3 (IC_50_ = 0.4 nM) with potencies that differed by ∼25,000-fold. In contrast, no such discrepancy was apparent when the inhibition of cytokine production from inflammatory cells (IC_50_ ∼0.5 *μ*M) and of PDE4 activity (IC_50_ ∼1.5 *μ*M) were compared (Boswell-Smith et al, 2006; Calzetta et al, 2013; Turner et al, 2020). This paradox may be explained by functional antagonism, which describes an inverse relationship between the degree of smooth muscle tone and the potency and pharmacological efficacy of a relaxant. Functional antagonism has been documented in ASM from several species and is more pronounced with ACh (and related agonists) than with histamine, 5-hydroxytryptamine, and leukotriene D_4_ (van den Brink, 1973; Torphy et al, 1983; Russell, 1984; Torphy, 1984; Roffel et al, 1995). For example, the EC_50_ of isoprenaline for relaxing bovine tracheal smooth muscle contracted with 10 nM (∼EC_20_), 100 nM (∼EC_70_), and 10 *μ*M MCh (EC_100_) was 0.65 nM, 81 nM, and 3.16 *μ*M, respectively (>4800-fold difference; Roffel et al, 1995). Similar data have been reported for the selective PDE3 inhibitor, siguazodan (SK&F 94836), on MCh-contracted canine ASM (Torphy et al, 1988). Logic dictates that ensifentrine, which like isoprenaline and siguazodan relaxes ASM by a cAMP-dependent mechanism, would be affected similarly both in vitro and in vivo. Indeed, the potency of ensifentrine for inhibiting electrical field stimulation-induced twitch responses of human bronchi, progressively decreased with increasing frequency of nerve stimulation (Calzetta et al, 2015). Thus, functional antagonism may help explain the erroneous description of ensifentrine as a hybrid PDE3/PDE4 inhibitor because the biochemical and functional outcomes of a bronchodilator cannot easily be compared.

TQC-3721 is being developed by Chia Tai Tianqing Pharmaceutical Group as a suspension for inhalation in subjects with moderate-to-severe COPD and is currently in phase II safety and efficacy clinical trials (NCT05987371). There are no preclinical data in the public domain about TQC-3721. Its structure has not been disclosed.

From a safety perspective, inhibition of PDE3 is of concern, given the well documented cardiovascular toxicity of this class of drugs and that subjects with COPD will require long-term therapy over many months or years. Although PDE3 inhibitors were developed to treat dilated cardiomyopathy, chronic dosing increased mortality (Movsesian, 2003; Amsallem et al, 2005). This could be problematic because ∼20% of people with COPD have right-side heart failure that is often secondary to PH (Naeije, 2003; de Miguel Díez et al, 2013). Chronic PDE3 inhibition could, therefore, be contraindicated even for a compound given by inhalation, and hence, pulmonary retention will be critical. In this respect, the peak plasma concentration of ensifentrine in 13 subjects with allergic asthma after inhalation of 0.018 mg/kg (o.d. for 6 days) was ∼2 ng/mL (4.2 nM; Zuiker, 2016). This dose equates to 1.26 mg/70 kg per individual, which is 42% of the 3 mg dose assessed in the 2 ENHANCE clinical trials (Anzueto et al, 2023). Adverse events were reported to be mild, although a reduction in blood pressure and a [compensatory] increase in heart rate were noted. The study investigators attributed these cardiovascular events to PDE3 inhibition in the vasculature, which was consistent with an increased incidence of headache and dizziness in 4 and 3 of the 13 subjects, respectively (Zuiker, 2016).

*Inhibitors of PDE4 and PDE7*. PDE7A is ubiquitously expressed in the lungs (Smith et al, 2003) and could represent a novel target for anti-inflammatory drugs (Giembycz and Smith, 2006a,b; Giembycz and Maurice, 2014; Jankowska et al, 2017). PDE7 was discovered in 1993 (Michaeli et al, 1993) yet 20 years elapsed before selective inhibitors became available and could be studied in biological systems (Nakata et al, 2002; Yang et al, 2003; Smith et al, 2004; Jones et al, 2007; Goto et al, 2009; Kadoshima-Yamaoka et al, 2009a,b,d). What emerged from those early investigations was unremarkable. However, there was some interest in the finding that the PDE7A inhibitor, BRL 50481, significantly enhanced the antimitogenic activity of the PDE4 inhibitor, rolipram despite being inactive alone (Smith et al, 2004). LPS-induced TNF*α* generation from human monocytes and lung macrophages was regulated similarly (Smith et al, 2004). This profile of activity was replicated with the dual PDE4/PDE7 inhibitor BC54, which inhibited TNF-*α* and IL-12 production from U-937 monocytic cells and Jurkat T-cells, respectively, and was more effective than rolipram, alone (de Medeiros et al, 2017). Collectively, these data are reminiscent of the behavior of PDE3 inhibitors and imply that additive or synergistic anti-inflammatory effects could be realized with a hybrid PDE4/PDE7 inhibitor (Giembycz, 2005a; Vijayakrishnan et al, 2007). To date, few in vivo studies have been reported implying that this hypothesis may not represent a viable approach. However, YM-393059, which is 45-fold more selective for PDE7 over PDE4, demonstrated efficacy in preclinical models of inflammation with a reduced emetic liability (Yamamoto et al, 2006a,b). Likewise, mice subjected to cigarette smoke-induced pulmonary inflammation were protected by prior endotracheal administration of antisense oligonucleotides directed against *Pde4b*, *Pde4d*, and *Pde7a* and that this intervention was superior to classical pharmacotherapy with roflumilast (Fortin et al, 2009).

*PDE3/PDE4 inhibitors and a LAMA.* Several patents have been filed describing the utility of combining ensifentrine with a LAMA (Walker et al, 2017, 2019). The inventions claim that a low concentration of a muscarinic receptor antagonist (eg, glycopyrronium) interacts synergistically with ensifentrine to relax medium and small human bronchi in vitro (Calzetta et al, 2013; Calzetta et al, 2015). This unexpected effect led to the proposal that in subjects with COPD, antagonizing the effects of endogenously released ACh from parasympathetic nerve fibers in the lung with a LAMA and raising the cAMP content in ASM with ensifentrine could produce added clinical benefit by reducing gas trapping in the lungs (ie, dynamic hyper-inflation), which is a common feature of COPD (Calzetta et al, 2015).

#### 7 Bifunctional ligands for COPD

An alternative to a hybrid inhibitor is a compound that contains 2 pharmacophores joined covalently by a rationally designed and inert “spacer” (Shonberg et al, 2011; Phillips and Salmon, 2012). In the context of respiratory diseases, these, so-called, bifunctional ligands have several advantages over their monofunctional parent compounds because of their relatively high molecular weights (often >1000 Da). This physical property often translates into enhanced pulmonary retention, low oral bioavailability, and reduced systemic exposure (Phillips and Salmon, 2012). The development of bifunctional ligands is also simplified because 2 pharmacophores in the same compound will have matched pharmacokinetics and identical deposition characteristics (Phillips and Salmon, 2012). Several bifunctional ligands containing a PDE4 inhibitor have been synthesized (Fig. 13). The most attractive “partners” for a PDE4 inhibitor have included a LAMA and a LABA in an attempt to harness both anti-inflammatory and bronchodilator activity at a similar dose (Giembycz and Maurice, 2014). The first example of a “**M**uscarinic receptor **A**gonist-**P**DE4 **I**nhibitor (ie, a MAPI) was the 4,6-diaminopyrimidine derivative, UCB-101333-3 (Provins et al, 2006). Given by inhalation to mice, this compound attenuated cigarette smoke-induced pulmonary neutrophilia and keratinocyte chemoattractant levels in BAL fluid and protected against the development of heavy metal-induced emphysema (Provins et al, 2007). Since that original report, the interest in developing MAPIs for COPD has continued including compound **10f** from Cheisi (Rizzi et al, 2023). This ligand is a fusion of tanimilast with a muscarinic receptor antagonist based on a phenylglycine scaffold. In vitro assays indicate that **10f** is a balanced molecule with an affinity and inhibitory potency at the muscarinic M_3_ receptor and PDE4B, respectively, of ∼1 nM (Rizzi et al, 2023). Moreover, in rodents, the compound proved suitable for inhaled dosing and displayed adequate lung retention and limited systemic exposure. Significantly, **10f** inhibited CCh-induced bronchoconstriction and ovalbumin-induced pulmonary eosinophilia in sensitized and challenged rats at the same dose with an acceptable duration of action (Rizzi et al, 2023).

Another means to achieve bronchodilator and anti-inflammatory activity in a single molecule is to couple pharmacophores that display PDE4 inhibitory activity and *β*_2_-adrenoceptor agonism. Support for this approach derives from the finding that roflumilast improved FEV_1_ in a group of patients with moderate-to-severe COPD who were being treated with LABA, salmeterol (Fabbri et al, 2009). Because PDE4 inhibitors are not thought to produce direct bronchodilation in humans (Grootendorst et al, 2003), the additional improvement in lung function is assumed to be secondary to the suppression of inflammation. Several bifunctional ligands have been described in which the head group of formoterol or salmeterol was fused to roflumilast or a phthalazone-based PDE4 inhibitor by a simple butyl spacer (Shan et al, 2012a; Liu et al, 2013). However, the activities of these compounds are unbalanced being more potent (440–2500-fold) *β*_2_-adrenoceptor agonists than inhibitors of PDE4. Improvements were achieved by modifying the spacer (to hexyloxyphenyl propanol or hexane), but *β*_2_-adrenoceptor agonism remained the dominant activity (Liu et al, 2013; Huang et al, 2014). Gilead Sciences has also reported the discovery of bifunctional LABA/PDE4 inhibitors for COPD, which have been optimized for inhaled delivery (Baker et al, 2011). In 1 example, an analog of the PDE4 inhibitor, GSK 256066 (Tralau-Stewart et al, 2011), was conjugated to a quinolinone-based orthostere (*β*2A)-derived from the LABA, indacaterol, to form the development candidate, GS-5759. This compound has an equal affinity (∼1 nM) for PDE4B and the human *β*_2_-adrenoceptor and represented a significantly improved ligand in having a balanced pharmacology for the 2 targets. In vitro, GS-5759 was active in a panel of assays where it inhibited the release of superoxide from human neutrophils, TNF*α*, IL-6, and CCL3 from human monocytes and ET-1, CCL5, CXCL10, and GM-CSF from human lung fibroblasts (Tannheimer et al, 2014); it also upregulated the expression of a plethora of genes in the BEAS-2B human airway epithelial cell line (eg, *DUSP1*, *CD200*, *CRISPLD2*, *CDKN1C,* and *FGFR2*) that have potential anti-inflammatory activity (Joshi et al, 2017). In vivo, GS-5759 was active in several preclinical models of COPD; it displayed bronchodilator activity in guinea pigs and dogs and inhibited LPS-induced pulmonary neutrophilia in rats, which was replicated in Cynomolgus monkeys (Salmon et al, 2014). Significantly, no emesis was produced in ferrets at doses of GS-5759 that were several orders of magnitude greater than its potency for inhibiting LPS-induced pulmonary leukocyte recruitment in the rat (Salmon et al, 2014). Despite these encouraging data, a primary therapeutic target of GS-5759 is the ASM, which likely displays a large *β*_2_-adrenoceptor reserve for *β*2A (Giembycz, 2009). This will probably render GS-5759 unbalanced because its potency as a bronchodilator will be greater, may be considerable, than its affinity for the *β*_2_-adrenoceptor and for inhibition of PDE4 (Giembycz, 2009; Joshi et al, 2017). The only way to overcome this limitation is to either increase the potency of the pharmacophore that inhibits PDE4 or reduce the affinity of *β*2A for the *β*_2_-adrenoceptor. Spare receptors represent a problem for the development of bifunctional ligands in general. This is a particular issue if 1 of the 2 pharmacophores is an agonist that is required to interact with different tissues for therapeutic benefit to be optimized.

An unexpected finding of these investigations was that GS-5759 had a 35-fold higher affinity for the *β*_2_-adrenoceptor than did *β*2A (Joshi et al, 2017). This pharmacological behavior has been reported previously for the bifunctional LABA/LAMA, THRX 198321 (Steinfeld et al, 2011), and may also apply to the MAPI, **10f** (Rizzi et al, 2023). Mechanistically, the enhanced affinity of these compounds for the *β*_2_-adrenoceptor may be due to positive allosterism (Steinfeld et al, 2011; Rizzi et al, 2023) or “forced proximity” binding (Hughes et al, 2011; Valant et al, 2012; Vauquelin and Charlton, 2013; Joshi et al, 2017). Regardless, the fact remains that the properties of bifunctional ligands are often distinct from their monofunctional parent compounds, which could reveal new opportunities for drug discovery (Fig. 13).

### D Liver disorders and PDEs

#### 1 The role of cyclic nucleotides in liver fibrosis and cirrhosis pathophysiology

Chronic liver disease results from ongoing hepatocyte damage caused by factors such as viruses, alcohol, and poor nutrition. It can lead to fibrosis, cirrhosis, and hepatocellular cancer, a major cause of morbidity and mortality (Rich, 2024). Affecting over a billion people globally, chronic liver disease causes more than a million deaths from cirrhosis each year (Disease et al, 2018). The most common causes are viral hepatitis, alcohol use, and metabolic dysfunction (Leszczynska et al, 2023). Following injury, hepatocytes release various chemokines and damage-associated molecular patterns, which attract and activate immune cells and hepatic stellate cells in the liver. Hepatic stellate cells (HSC) have very important functions in the liver including the storage of vitamin A, antigen presentation, and wound healing. However, during persistent liver injury, HSC undergo activation and become proliferative, migratory myofibroblasts (Wang and Friedman, 2023). HSC myofibroblasts are the main source of extracellular matrix proteins (ECM) in fibrotic liver. Excessive accumulation of ECM in the liver tissue results in deterioration of liver function, leading to cirrhosis and liver failure.

cAMP and cGMP signaling has been studied in various liver cell functions, including hepatocytes, macrophages, T cells, and hepatic stellate cells (Wahlang et al, 2018; Elnagdy et al, 2020; Elnagdy et al, 2023). The first studies related to alcohol-associated liver disease (ALD) reported a lower level of cAMP in peripheral blood mononuclear cells of alcoholic hepatitis (AH) patients with immune dysfunction (Barlas et al, 1983) and alcohol use disorders (Diamond et al, 1987). Effects of chronic alcohol exposure on cAMP levels were later shown in human and murine monocytes and macrophages, including liver resident macrophages or Kupffer cells (Gobejishvili et al, 2006). Importantly, this decrease in cAMP and its signaling was identified as a critical mechanism of macrophage “priming” to produce increased levels of TNF*α* in response to endotoxin (Gobejishvili et al, 2006; Gobejishvili et al, 2008).

Persistent hepatocyte injury and inflammation will result in the activation of HSC in the liver and their trans-differentiation to myofibroblasts (the main producers of extracellular matrix proteins). Because transdifferentiation of HSCs plays a key role in the development of liver fibrosis, targeting HSC activation has become a focal point in treating liver fibrosis (Li et al, 2008). Upon activation, HSCs express alpha-smooth muscle actin (*α*SMA) and produce ECM proteins like collagens and fibronectin. Perhaps the most profibrogenic cytokine leading to HSC activation is transforming growth factor *β*1 (TGF*β*1). cAMP-elevating agents and agonists have been shown to inhibit TGF*β*1-induced expression of *α*SMA and collagen in different cell types (Houglum et al, 1997; Desmouliere et al, 1999; Liu et al, 2006; Cortijo et al, 2009; Insel et al, 2012; Garrison et al, 2013). Indeed, EPAC1 has been identified as a critical regulator of TGF*β*1 signaling in various tissue fibroblasts (Yokoyama et al, 2008; Insel et al, 2012), including in HSCs. Recent studies showed that TGF*β*1 decreases cAMP and EPAC1 levels in HSCs, which contributes to their activation (Schippers et al, 2017; Elnagdy et al, 2023).

#### 2 Role of cAMP-PDE

Because inflammation plays a major role in chronic liver disease, anti-inflammatory strategies using PDE inhibitors have been utilized to evaluate their beneficial effect on liver injury. Moreover, inflammation and dysregulated hepatocyte function in experimental models of liver injury and fibrosis have been shown to be accompanied by increased expression of PDE enzymes in the liver (Gobejishvili et al, 2013; Avila et al, 2016; Essam et al, 2019). A pathogenic role of PDE enzymes in the development of liver injury has been confirmed by demonstrating that PDE inhibitors alleviate liver damage and inflammation (Gobejishvili et al, 2013; Essam et al, 2019; Rodriguez et al, 2019; El-Deen et al, 2020; Ma et al, 2022; Elnagdy et al, 2023; Tao et al, 2023). More specifically, the effect of cAMP in macrophage priming was shown to be mediated by increased activity and expression of PDE4, specifically PDE4B (Gobejishvili et al, 2008). PDE4 inhibition resulted in a significant decrease in endotoxin-induced inflammatory cytokine production by human peripheral blood mononuclear cells (PBMCs) from patients with alcohol-associated hepatitis and monocytes/macrophages of human and murine origin (Gobejishvili et al, 2008; Gobejishvili et al, 2011; Gobejishvili et al, 2013; Rodriguez et al, 2019). Importantly, studies using gene knockout mice identified PDE4B as an essential player in endotoxin-mediated production of TNF*α* (Jin and Conti, 2002; Jin et al, 2005).

However, the role of PDE4-regulated cAMP signaling is not limited to immune cells and their inflammatory responses. Importantly, increased expression of PDE4 enzymes has been associated with both spontaneous and TGF*β*1-induced activation of HSCs (Gobejishvili et al, 2013; Elnagdy et al, 2023). Moreover, recent work demonstrated that PDE4A, B, and D are upregulated in the livers of patients with metabolic dysfunction-associated steatotic liver disease (MASLD), and their levels are positively correlated with TGF*β*1 (Elnagdy et al, 2023). Importantly, PDE4 enzymes are also expressed in activated HSCs/myofibroblasts in human and mouse livers (Elnagdy et al, 2023). PDE4 inhibition significantly attenuated cytoskeleton remodeling and HSC migration both in vitro and in vivo leading to reduced collagen deposition and fibrosis (Elnagdy et al, 2023).

In addition to extracellular matrix remodeling, cAMP signaling plays a significant role in glucose and lipid metabolism in hepatocytes [reviewed in Wahlang et al (2018)]. In relevance to ALD, studies have shown that alcohol attenuates cAMP/PKA signaling in hepatocytes, which results in a decrease in the gene (Cpt1a) encoding carnitine palmitoyltransferase 1A and fatty acid *β*-oxidation (Elnagdy et al, 2020). Inhibition of PDE4, and specifically PDE4B, prevents alcohol-mediated decrease in cAMP signaling and Cpt1 expression and prevents alcohol-induced lipid accumulation in the liver (Avila et al, 2016; Ma et al, 2022). The effect of PDE4 inhibition on alcohol-induced ER stress has also been shown (Rodriguez et al, 2019). Notably, PDE4 inhibition decreases an alcohol-mediated increase in JNK activation and hepatocyte death (Rodriguez et al, 2019). More recent studies showed that overexpression of PDE4D in the liver led to the development of MASLD in mice, which was attenuated by a PDE4 inhibitor (Tao et al, 2022b; Tao et al, 2023).

#### 3 cGMP and dual substrate PDEs

Besides PDE4, recent papers have demonstrated the role of PDE9 and 10 in liver and lung fibrosis as well as diet-induced obesity (Ceddia et al, 2021; Mishra et al, 2021; Wu et al, 2021; Li et al, 2023), indicating that cGMP signaling is also critical in tissue fibrogenesis. Indeed, a recent study reported increased hepatic levels of cGMP in patients and mice with alcohol-associated steatohepatitis (ASH; Montoya-Durango et al, 2023). Moreover, these changes were associated with significant alterations in various cAMP- and cGMP-selective PDEs in the liver, highlighting the potential role of PDE enzymes in the pathogenesis of ASH. Given the crucial role of NO-cGMP signaling in the regulation of hepatic sinusoids and portal pressure, perhaps the most significant findings presented in this recent study were increased levels of PDE1A, PDE4A, PDE4D, and PDE5A (Montoya-Durango et al, 2023). Indeed, these enzymes have been shown to modulate vascular tone, remodeling, and exchange via both cAMP and cGMP (Houslay et al, 2007; Netherton et al, 2007). Increased levels of soluble guanylyl cyclase and PDE5 in cirrhotic livers have been reported in another human study (Kreisel et al, 2021). In a normal liver, PDE5 protein is highly expressed in perisinusoidal cells with a very weak expression in hepatocytes. In cirrhotic livers, PDE5 expression increases in fibrous septa, and perisinusoidal cells throughout the parenchyma (Kreisel et al, 2021). PDE5 inhibitors have shown anti-inflammatory and antifibrotic properties in several studies, as well as improvement in portal hypertension (Knorr et al, 2008; Choi et al, 2009; Deibert et al, 2018; Schaffner et al, 2018; Brusilovskaya et al, 2020; Kreisel et al, 2020). Notably, a recent study reported the age-dependent sexual dimorphism in the vascular PDE expression patterns (Wang et al, 2021). This is the first study to highlight sexual dimorphism in PDE expression. Future studies are needed to examine whether there are sex-dependent differences in PDE expression in the liver and whether it contributes to the susceptibility of females to certain types of liver injury.

Interestingly, differential beneficial effects of various PDE inhibitors on a high fat-induced MASLD model in rats have been reported (El-Deen et al, 2020). Specifically, when administered in a treatment paradigm, the authors observed that pentoxifylline (PTX), a broad-spectrum PDE inhibitor, had the strongest effect on oxidative stress markers, steatosis, inflammation, and liver injury when compared with cilostazol and sildenafil (El-Deen et al, 2020). Notably, PTX (alone and in combination with other drugs) has been widely used to treat MASLD and ALD in humans (Zein et al, 2011; Zein et al, 2012; Smart et al, 2013; Alam et al, 2017; Cioboata et al, 2017; Louvet et al, 2018; Teschke, 2018; Culafic et al, 2020; Marot et al, 2020; Szabo et al, 2022). However, although several studies have reported significant beneficial effects, including anti-fibrotic and antioxidative stress (Zein et al, 2011; Zein et al, 2012; Sridharan et al, 2018; Fouda et al, 2021; Kedarisetty et al, 2021), the use of PTX has been debated (Van Wagner et al, 2011). PTX therapy has shown mixed results in trials in patients with AH (Akriviadis et al, 2000; Thursz et al, 2015; Louvet et al, 2018; Kedarisetty et al, 2021; Philips et al, 2022; Duan et al, 2023), with the large STOPAH trial reporting lack of efficacy in reducing mortality (Thursz et al, 2015). Subjects with alcohol use disorder (AUD) are reported to be less compliant with many medical regimens. PTX is a nonselective and weak PDE inhibitor and requires 3 times a day (t.i.d.) dosing. Patients report significant gastrointestinal upset which causes noncompliance. Compliance with PTX in the most recent large AH trial was 49.4% ± 40.2% versus placebo 65.5% ± 35.3% (*P* = .06) with nausea being the primary reason for noncompliance (Szabo et al, 2022). The American College of Gastroenterology (ACG) most recent clinical guidelines for the treatment of ALD do not support the use of PTX for severe AH due to a moderate level of evidence that PTX provides survival benefits (Jophlin et al, 2024). More clinical trials are ongoing to test PTX for the treatment of metabolic dysfunction-associated steatohepatitis (MASH; NCT05284448) and to prevent decompensation in stable cirrhotic patients with prior decompensation (NCT06041932).

Based on strong preclinical evidence that PDE4 inhibition has anti-inflammatory and antifibrotic properties, the efficacy of the PDE4 inhibitor, ASP9831, was tested in a proof-of-concept phase 2 clinical trial in biopsy-confirmed patients with MASH and liver fibrosis (Ratziu et al, 2014). The trial did not find any effects of the PDE4 inhibitor on biochemical end points including liver injury markers (ALT, AST, and cytokeratin 18), adiponectin, and TNF*α* (Ratziu et al, 2014). The authors questioned the benefit of PDE4 inhibition as a therapy for MASH due to the failure to attenuate inflammation and liver injury markers. However, although a liver biopsy was performed prior to patient enrollment, no follow-up biopsies were documented. Hence, it is unknown if ASP9831 had any effect in improving the fibrosis stage. Moreover, this study was only 12 weeks in duration; thus, there were multiple study limitations. Notably, authors pointed out that detailed studies examining the role of each of PDE4 enzymes in the pathogenesis of MASH are lacking. In this regard, a recent study found that PDE4A, B, and D enzymes are expressed in myofibroblasts in the livers of MASH cirrhosis patients (Elnagdy et al, 2023), implicating the role of these enzymes in MASH cirrhosis.

**Fig. 14:**
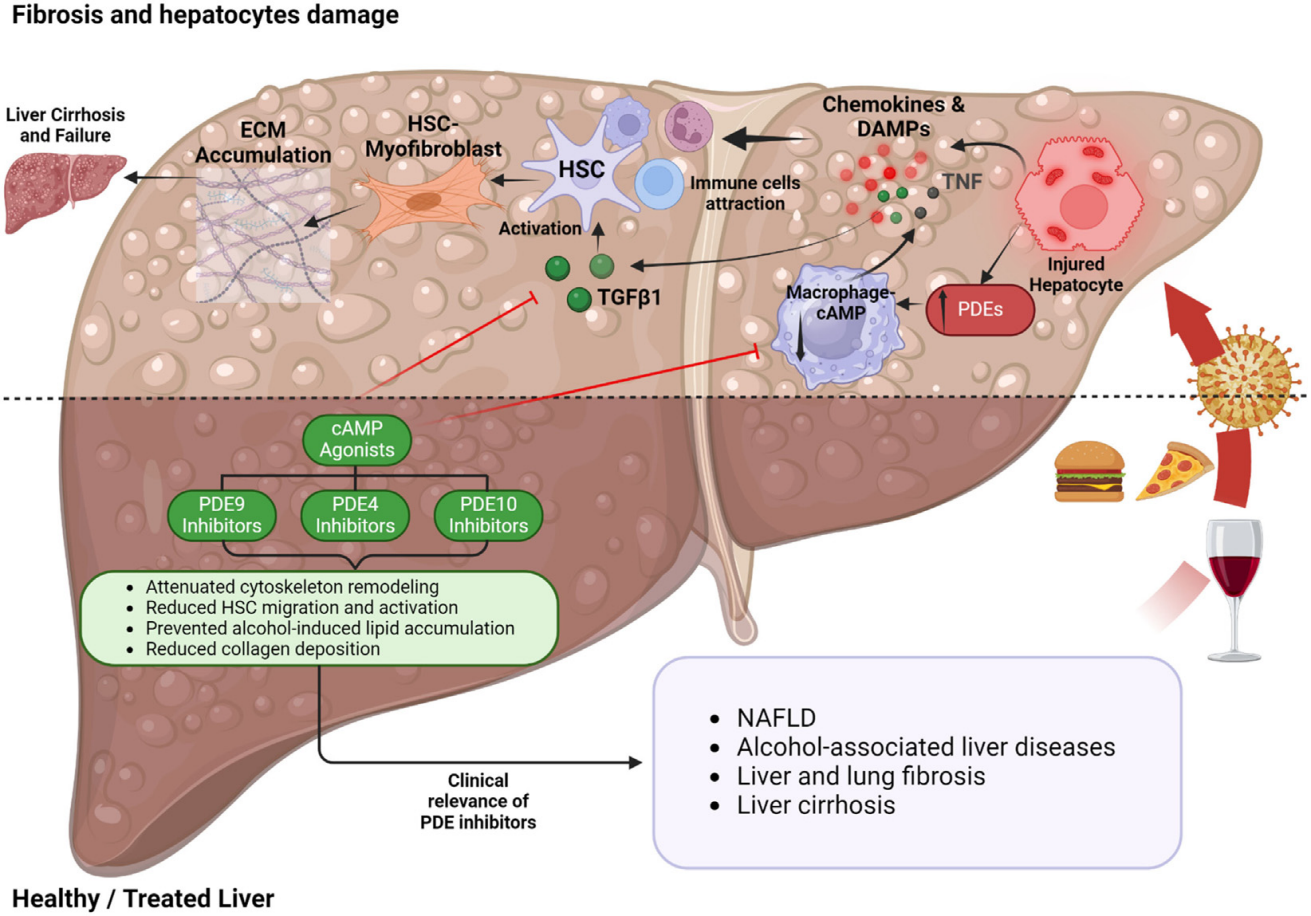
Involvement of PDEs in liver fibrosis and hepatocyte damage. Several factors, including alcohol, viruses, and high-fat diets, can instigate damage to hepatocytes and fibrosis. Injured hepatocytes release inflammatory mediators leading to the recruitment of peripheral immune cells and activation of resident macrophages consequently, triggering the activation of HSCs. Activated HSCs transdifferentiate into pro inflammatory and profibrogenic myofibroblasts and release excessive amount of ECM, which in long-term leads to liver fibrosis. Elevated PDE activity mediates some of these pathological changes, thus presenting as potential pharmacological targets for treating liver disorders. Created with BioRender.com.

In summary, there is ample preclinical and clinical evidence that PDE enzymes are involved in various liver cell (dys)functions associated with liver pathology. However, more work needs to be done to characterize the expression patterns and levels of PDEs in a cell-specific manner in the liver (Fig. 14). This will allow us to better understand their role in liver cell pathophysiology for therapeutic targeting. Moreover, liver diseases are multifactorial and progressive with various stages of pathology ranging from simple steatosis to inflammation and fibrosis. Animal models do not recapitulate the clinical progression of human liver disease, and hence, they have limited utility in testing PDE inhibitors for efficacy. Because various selective PDE4 and PDE5 inhibitors have been approved by the FDA for clinical use, perhaps hepatologists will consider them as therapeutic options for liver diseases, especially for ALD and MASLD.

### E Neurodevelopmental disorders

#### 1 PDE isoforms, cognitive impairment, and Alzheimer

##### a Neuronal physiology and pathophysiology

Our memories are what define who we are as a person. Healthy aging, alongside age-related diseases including cognitive decline (ACRD), mild cognitive impairment (MCI), AD, AD-related dementias (ADRD), and Huntington’s disease (HD), is characterized by memory deficits and other cognitive impairments (Apple et al, 2017; Dean et al, 2017; Ffytche et al, 2017; Kane et al, 2017). These cognitive domains are known to be regulated by PDEs and aging and age-related diseases of the brain have been associated with significant disturbances in cyclic nucleotide signaling (cf, Kelly, 2018a). Here, we suggest that dysfunction in more than 1 PDE contributes to age-related pathology and identify those PDE families and isoforms with the greatest therapeutic potential.

**Fig. 15:**
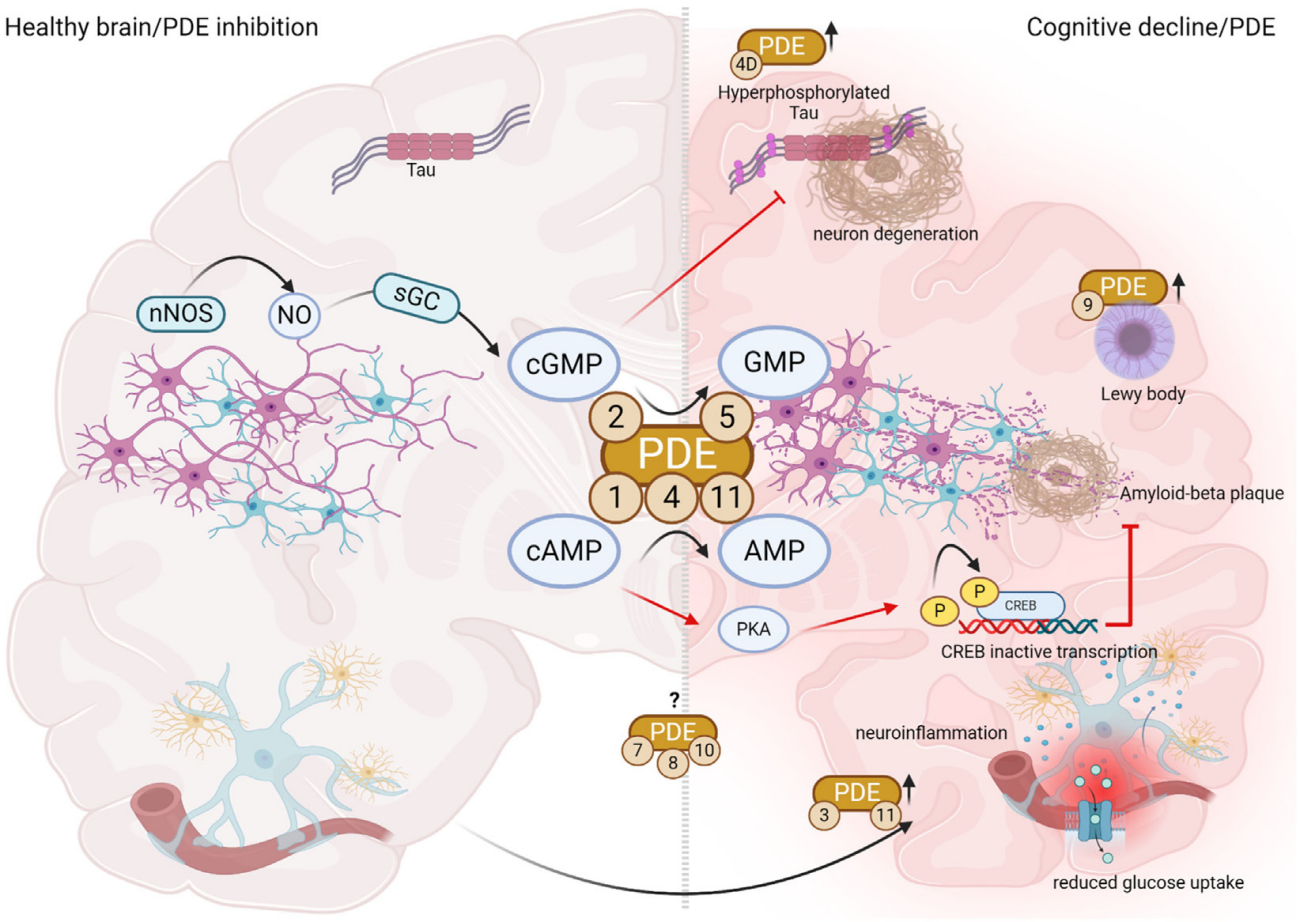
Role of PDEs in pathophysiology of neurodevelopmental disorders. PDEs inhibitors increase activation of protein kinase A (PKA), leading to increased activation of CREB transcription. This process contributes to the prevention of formation and toxicity of amyloid beta plaques in neurodegenerative disorders like Alzheimer disease. Additionally, changes in PDE expression can affect neuroinflammation and reduces cerebral glucose uptake which as a primary energy source for brain could lead to development of neurodegenerative disorders. Red arrows indicate the effect of PDE inhibitors. Question marks imply the unknown role of these PDE subtypes in neurodevelopmental disorders. Created with BioRender.com.

As described in detail below, several PDEs demonstrate alterations in expression, localization, and/or activity in the aged brain that can be subverted/exacerbated in the context of ACRD, MCI, AD, ADRD, and HD. A particularly challenging aspect of this area of research is that these functional changes are often isoform-specific and vary across brain regions. For example, in the hippocampus and cortex, aged rodents demonstrate increased high *K*_m_ cAMP-PDE and cGMP-PDE hydrolytic activity relative to young rodents (Stancheva and Alova, 1991; Chalimoniuk and Strosznajder, 1998). As such, isoform-selective therapeutics have been much pursued for the treatment of ARCD, MCI, AD, ADRDs, and HD (Fig. 15).

##### b PDE1

During the transition from early to late adulthood, rodent studies show that PDE1B mRNA expression remains unchanged in the hippocampus but decreases in the cerebellum and striatum (Kelly et al, 2014). In contrast, PDE1C mRNA expression increases in the striatum and PDE1C1 protein—but not PDE1C3—increases in the hippocampus (Kelly et al, 2014). Expression of PDE1A mRNA in these brain regions remains unchanged with age, and no isoform showed age-related changes in cortical expression (Kelly et al, 2014). Functionally, PDE1B is the most explored PDE1 isoform in the context of learning and memory. PDE1B knockout mice performed equivalently to wild-type mice in both the passive avoidance and conditioned avoidance tests (Siuciak et al, 2007b). Adolescent PDE1B knockout and heterozygous mice showed impaired spatial learning and memory in a hidden-platform water maze task relative to wild-type mice (Reed et al, 2002); however, adult PDE1B knockout mice showed intact spatial learning and memory but impaired reversal learning on the task (Ehrman et al, 2006). In stark contrast, viral knockdown of only hippocampal PDE1B expression in young adult mice enhanced contextual fear conditioning memory and spatial memory in the Barnes maze (McQuown et al, 2019). Thus, although the general knockdown of PDE1B across brain regions impaired memory processes, local deletion in the hippocampus improved memory function. PDE1B is highly expressed in many brain regions other than the hippocampus, including the cortex, striatum, thalamus, and brain stem (Kelly, 2014; Kelly et al, 2014). Thus, an effect of PDE1B deletion in one of these other brain regions may mask any nootropic effect related to deletion within the hippocampus.

That said, the nonselective PDE1 inhibitor, vinpocetine, is sold in over-the-counter supplements (eg, Cavinton or Intelectol, Richter Gedeon; Cognitex, Life Extension) that claim to improve memory (Baillie et al, 2019). Indeed, several clinical trials have examined the cognition-enhancing effects of vinpocetine—either alone or in combination with other compounds (eg, caffeine or Ginko Biloba)—and have generally found improvement in healthy volunteers, individuals with cerebral hypofusion, and possibly aged individuals, but no improvement in AD patients (Subhan and Hindmarch, 1985; Balestreri et al, 1987; Thal et al, 1989; Hindmarch et al, 1991; Polich and Gloria, 2001; Szatmari and Whitehouse, 2003; Richter et al, 2011b; Valikovics et al, 2012; Caldenhove et al, 2017). This is consistent with preclinical rodent models demonstrating therapeutic effects of PDE1 inhibitors in vascular dementia via the PKA-CREB pathway (Zhou et al, 2023), but not with studies showing the efficacy of PDE1 inhibitors in rodent models of AD (Shekarian et al, 2020; Shekarian et al, 2023). Reports of side effects associated with vinpocetine have been minimal, and include flushing, rashes, and minor gastrointestinal disturbances (Smith and Doe, 2002).

Intracellular therapies developed the broad PDE1 inhibitor, lenrispodun, which shows picomolar IC_50_s for PDE1A, PDE1B, and PDE1C in enzymatic assays and >1000-fold selectivity versus its nearest neighbor PDE4 (Li et al, 2016b; Snyder et al, 2016). Lenrispodun demonstrates cognition-enhancing effects in rodent models of long-term memory and working memory deficits (Snyder et al, 2016; Li et al, 2016b; Pekcec et al, 2018). It remains to be determined whether the cognition-enhancing effects of lenrispodun are due to inhibition of PDE1A, PDE1B, and/or PDE1C; however, PDE1B may be the most likely candidate given its expression in dopamine D_1_-expressing neurons (Pekcec et al, 2018) along with the fact that a PDE1B-selective inhibitor developed by Dart Neuroscience showed similar cognition-enhancing effects (Dyck et al, 2017).

##### c PDE2

Studies in humans report that PDE2A mRNA levels increase between the prenatal period and childhood and then stabilize into young-middle adulthood in cortical regions, amygdala, and striatum, whereas hippocampal expression of PDE2A mRNA does not increase beyond prenatal levels until adulthood (Farmer et al, 2020). In stark contrast, studies in rodents report decreased expression of PDE2A in the striatum during the transition from early to late adulthood, but no change in the hippocampus, cortex, or cerebellum (Kelly et al, 2014). PDE2A expression did not change as a function of AD in the hippocampus, cortex, cerebellum, or striatum (Reyes-Irisarri et al, 2007).

As extensively reviewed elsewhere (Gomez and Breitenbucher, 2013; Zhang et al, 2017a; Kelly, 2018a; Nakashima et al, 2019; Ruan et al, 2019; Zhou et al, 2021; Shi et al, 2021a; Yan et al, 2022), PDE2 inhibitors improve many types of memory in young and old rodents as well as in AD rodent models, preventing A*β*-induced cytotoxicity. The ability of PDE2 inhibitors to improve cognition in these models appears to be mediated via its regulation of cGMP signaling because the nootropic effects require signaling via nNOS (Domek-Lopacinska and Strosznajder, 2008) and PKG (Wang et al, 2017a). Takeda Pharmaceuticals initiated Phase I trials with the PDE2 inhibitor, TAK-915, to correlate plasma exposures with central target engagement to inform dose selection for future trials targeting cognitive impairments (Mikami et al, 2017a,b,c); however, clinicaltrials.gov does not show any trials registered beyond Phase I (accessed November 28, 2023).

##### d PDE3

AD upregulates PDE3 expression in cerebral vessels (Maki et al, 2014). In preclinical models, PDE3 inhibitors have prevented or reversed A*β*-induced cytotoxicity, both in vitro and in AD mouse models (cf, Yanai et al, 2017; Kelly, 2018a; Yanai et al, 2022). Several prospective and retrospective studies have examined cilostazol as a primary or adjunctive treatment for cognitive deficits associated with AD and schizophrenia (Arai and Takahashi, 2009; Shirayama et al, 2011; Sakurai et al, 2013; Taguchi et al, 2013; Ihara et al, 2014; Tai et al, 2017a,b). As reviewed elsewhere (Heckman et al, 2018a), most of these studies demonstrated positive effects of cilostazol on cognition. The mechanism by which cilostazol elicits improved cognition has yet to be determined empirically. Given there is very little expression of PDE3A or PDE3B in the brain (Lakics et al, 2010; Kelly et al, 2014), it may be more likely that cognition-enhancing effects of cilostazol are driven by increased cerebral blood flow that comes with chronic—but not acute—dosing (Mochizuki et al, 2001; Birk et al, 2004; Kai et al, 2011). Indeed, recent studies in mice suggest the ability of cilostazol to reverse age-related impairments in hippocampus-dependent memory is related to effects on the blood-brain barrier (Yanai et al, 2017) along with increased cerebral glucose uptake and reduced neuroinflammation (Yanai et al, 2022). Despite its existing FDA approval, the efficacy and safety of cilostazol (*Pletal*) is still very much a topic of investigation (cf, Baillie et al, 2019).

##### e PDE4

The PDE4 family is arguably the most studied of all the PDE families (cf, Baillie et al, 2019; Kelly et al, 2020), with PDE4A, PDE4B, and PDE4D, but not PDE4C, being expressed in the rodent (Kelly, 2014; Kelly et al, 2014) and human brain (Lakics et al, 2010). Interestingly, hippocampal PDE4 protein expression appears to decrease from early to late adulthood (Tohda et al, 1996; Kato et al, 1998; Harada et al, 2002); however, genetic deletion or broad inhibition of PDE4 rescued many types of A*β*-induced cytotoxicity and age-related decline, including reduced CREB phosphorylation, long-term potentiation deficits, and memory impairments [(Bach et al, 1999; de Lima et al, 2008; Drott et al, 2010; Devan et al, 2014; Kumar and Singh, 2017); cf, (Kelly, 2018a; Baillie et al, 2019b); see more below]. Different studies suggest that individual PDE4 isoforms are differentially affected by the disease in a brain region-specific manner (Perez-Torres and Mengod, 2003; Sebastiani et al, 2006; McLachlan et al, 2007; Paes et al, 2021a).

Despite the fact that the broad spectrum PDE4 inhibitor rolipram triggered aging-like impairments in working memory in young adult monkeys (Ramos et al, 2003), more recent studies report nootropic effects of various PDE4 inhibitors in the elderly, patients with schizophrenia, and other human populations (cf, Baillie et al (2019b); see more below). Zembrin is a nonselective PDE4 inhibitor (it also acts as a 5-HT uptake inhibitor) that is not FDA-approved but is a component of a number of herbal supplements claiming calming or mood-stabilizing properties (eg, Calm, Doctor’s Best; Mood, Procera; Nutri-calm, and Nature’s Sunshine; Terburg et al, 2013). Roflumilast has also been tested for its ability to improve cognition and information processing in healthy humans (Heckman et al, 2018b; Van Duinen et al, 2018).

The cognition-enhancing effects of roflumilast described above are consistent with a press release from Dart Neuroscience claiming that 45mg of their PDE4 inhibitor, HT-0712, the lowest dose tested, improved long-term memory for word lists in elderly subjects experiencing a cognitive decline (Baillie et al, 2019). Like Dart Neuroscience, Tetra Therapeutics appears to be pursuing an indication related to cognitive functioning for their PDE4D-negative allosteric modulator zatolmilast. These effects in humans are again consistent with preclinical studies showing zatolmilast improved a number of behaviors in a mouse model of Fragile-X Syndrome and antagonized the amnestic effects of scopolamine in mice (Gurney et al, 2017; Zhang et al, 2018a). Preclinical cognition-enhancing effects of GSK’s PDE4 inhibitor, GSK 356278 were similarly reported (Rutter et al, 2014) as were the ability of several PDE4 inhibitors to ameliorate memory deficits and pathology in dementia-related rodent models (Feng et al, 2019; Liang et al, 2020; Wang et al, 2020; Nazir et al, 2021; Virk et al, 2021; Xia et al, 2022; Hasan et al, 2022; Cong et al, 2023; Gomaa et al, 2023).

From a therapeutic perspective, then, it would be preferable to only target these isoforms in relevant brain regions. As described below, select PDE4A and PDE4D splice variants are the most likely to play a role in molecular mechanisms of memory because numerous studies have reported that genetically manipulating all/select PDE4B isoforms alter synaptic plasticity but largely have no effect on learning and memory (Siuciak et al, 2008a; Zhang et al, 2008a; Rutten et al, 2011; Campbell et al, 2017).

*PDE4A*. Many studies have analyzed the effects of genetically manipulating PDE4A on memory. *PDE4A* knockout mice exhibit normal object recognition memory and spatial water maze memory yet improved passive avoidance memory relative to wild-type mice (Hansen et al, 2014). The selective effect on passive avoidance memory may be related to the aversive nature of the stimuli employed in passive avoidance, given the fact that PDE4A deletion produces anxiogenic-like phenotypes on the elevated-plus maze, light-dark transition, and novelty-suppressed feeding tests (Hansen et al, 2014). This is highly interesting, given that negatively valanced memories are thought to trigger a stronger encoding, storing, and reactivation of sensory detail in controls (Hansen et al, 2014) and patients with AD (Maria and Juan, 2017). As extensively reviewed elsewhere (Baillie et al, 2019), each PDE(4) isoform is uniquely anchored by protein-binding partners through its unique *N*-terminal domain, which leads to the regulation of different nanodomains of cAMP. When full-length PDE4A5 that includes its unique *N*-terminal targeting domain is virally overexpressed in hippocampal excitatory neurons, forskolin-induced hippocampal late long-term potentiation (LTP) is impaired and hippocampus-dependent long-term, but not short-term, memory for object location and contextual fear conditioning is attenuated (Havekes et al, 2016a). In contrast, hippocampal delivery of a catalytically dead PDE4A5 that displaces endogenous PDE4A5 (ie, a dominant negative approach) rescued localized cAMP signaling deficits and hippocampus-dependent memory impairments that were caused by sleep deprivation (Vecsey et al, 2009; Havekes et al, 2016a,b). Interestingly, overexpression of PDE4A5 in the hippocampus did not alter anxiety-related behaviors in this study, suggesting either that the PDE4A isoforms regulating anxiety may differ from those regulating memory function or that PDE4A5 expression outside of the hippocampus (eg, in the amygdala or prefrontal cortex) regulates anxiety-related behaviors (Kelly et al, 2020). Importantly, overexpression of PDE4A1, which targets different hippocampal nanodomains, leaves memory undisturbed (Havekes et al, 2016a). As such, compounds or biologicals that target the unique *N*-terminal domain of each individual PDE4A isoform may be necessary for a beneficial effect in the context of cognitive deficits [as described in Baillie et al (2019)].

*PDE4D*. Expression of PDE4D has been genetically and pharmacologically manipulated to examine its role in hippocampus-dependent memory and plasticity. Genetic deletion of PDE4D strengthened recent long-term memory in the radial arm maze, hidden platform water maze, and object recognition tests while increasing levels of cell proliferation and phosphorylation of CREB in the mouse hippocampus (Li et al, 2011). That said, weaker recent long-term memory for contextual fear conditioning was also observed in the global PDE4D knockout mouse (Rutten et al, 2008). It is quite possible that this particular memory impairment may reflect the loss of PDE4D outside the hippocampus, particularly from the amygdala, because selective knockdown of *PDE4D* in the hippocampus alone improved recent long-term memory for contextual fear conditioning while increasing the number of training-induced stubby spines in CA1 (Baumgartel et al, 2018). Furthermore, *PDE4D* knockout mice require less tetanic or theta burst stimulation to induce long-term potentiation relative to wild-type mice, although the maximum strength of LTP obtained in *PDE4D* knockout mice matches that of wild-type mice (Rutten et al, 2008). Furthermore, PDE4D expression is downregulated via gene methylation in aging rats undergoing a moderate-intensity intermittent training program that attenuated ARCD of spatial learning and memory while improving the synaptic structure of the hippocampus (Zhang et al, 2023b). Similarly, PDE4D miRNA infused selectively into the prefrontal cortex reversed A*β*1-42-induced cognitive impairment (Shi et al, 2021b). That said, PDE4D was reported to be upregulated in the prefrontal cortex of aged rats in a manner that positively correlated with working memory and inversely correlated with Tau phosphorylation (Leslie et al, 2020). Thus, the role of PDE4D in regulating memory appears to be brain region and memory type specific.

It is likely the long forms of PDE4D, specifically, are negative regulators of hippocampus-dependent memories. Infusion of miRNAs within the dentate gyrus of the hippocampus that targets PDE4D4 and PDE4D5 strengthened recent long-term memory in the radial arm maze, hidden water maze, and object recognition tests (Li et al, 2011); however, infusion of miRNAs targeting PDE4D1/2 or PDE4D3 did not. PDE4D4 and PDE4D5 miRNAs also rescued A*β*-42-induced memory deficits in the hidden platform water maze and object recognition tasks (Zhang et al, 2014). Knockdown of *PDE4D* long forms also increased phosphorylation of CREB in the hippocampus (Li et al, 2011), a reduction of which is associated with aging (Kelly, 2018a). When long forms of PDE4D were knocked down in the prefrontal cortex of mice, novel object recognition and spatial memory were similarly improved, as was phosphorylation of CREB and pyramidal neuron dendritic branching/length (Wang et al, 2013). Furthermore, knockdown of PDE4D long forms in the prefrontal cortex rescued memory impairments in mice undergoing chronic unpredictable stress (Wang et al, 2015b). Thus, PDE4D4 and PDE4D5, both within and outside of the hippocampus, play critical roles in constraining neuroplasticity and memory formation. Together, these data suggest that PDE4A5, PDE4D4, and PDE4D5 may be the key PDE4 splice variants to target in the treatment of memory deficits.

##### f PDE5

PDE5 is probably best known as a drug target for erectile dysfunction (cf, Baillie et al, 2019); however, several studies have pointed to a potential role in regulating brain function. In rodent cerebellum, PDE5A mRNA expression increases between early to late adulthood (Kelly et al, 2014). In animal models, inhibitors of PDE5A have provided protection against age-related decline (Domek-Lopacinska and Strosznajder, 2008; Orejana et al, 2012; Palmeri et al, 2013; Devan et al, 2014). Still, a number of clinical trials have tested the effects of the PDE5 inhibitors tadalafil, sildenafil, and vardenafil on various measures of cognition in healthy volunteers, patients with schizophrenia, or elderly patients with cerebral small vessel disease and have largely found no effects (Grass et al, 2001; Schultheiss et al, 2001; Goff et al, 2009; Reneerkens et al, 2013a,b; Pauls et al, 2023). In contrast, the temporal cortex of patients with AD shows a 5× increase in PDE5A expression relative to controls (Ugarte et al, 2015). Furthermore, PDE5A inhibitors rescue memory deficits, synaptic dysfunction, Tau hyperphosphorylation, A*β* burden, and cytotoxicity in AD mouse models (Puzzo et al, 2009; Garcia-Barroso et al, 2013; Cuadrado-Tejedor et al, 2011; Fiorito et al, 2013; Zhang et al, 2013; Puzzo et al, 2014; Acquarone et al, 2019; Zhu et al, 2019a; Huang et al, 2020b; Tabrizian et al, 2021; Kang et al, 2022; Justo et al, 2023) in a PKG-dependent manner (Zhang et al, 2013).

##### g PDE7

Expression of PDE7A mRNA decreases in rodent cortex from early to late adulthood (Kelly et al, 2014). This age-related reduction in PDE7A mRNA is particularly interesting given that a SNP in PDE7A has been genetically associated in humans with age-related cognitive decline (De Jager et al, 2012; Andrews et al, 2016) and cognitive dysfunction related to brain tumors (Correa et al, 2019). In contrast, PDE7A mRNA is decreased in CA2 of the hippocampus in patients with AD relative to controls (Perez-Torres et al, 2003). Thus, it may be surprising that PDE7 inhibitors have positive effects in models of diseases where cognition, neuroprotection, neuroinflammation, and/or motor function are impaired (Banerjee et al, 2012; Redondo et al, 2012; Perez-Gonzalez et al, 2013; Lipina et al, 2013; Garcia et al, 2014; Morales-Garcia et al, 2014; Morales-Garcia et al, 2015a,b; Mestre et al, 2015; Jankowska et al, 2017; Morales-Garcia et al, 2017), including models of AD (Perez-Gonzalez et al, 2013; Bartolome et al, 2018).

##### h PDE8

PDE8A3 and PDE8A4/5 protein expressions increase in the rodent hippocampus from early to late adulthood (Kelly et al, 2014; Hegde et al, 2016), whereas PDE8A1 protein levels do not change in this brain region (Kelly et al, 2014). PDE8A mRNA levels also increase across the lifespan in the rodent striatum (Kelly et al, 2014). In contrast, PDE8B mRNA is increased in hippocampal CA2 of patients with AD (Perez-Torres et al, 2003) and in vitro in response to an accumulation of carboxy-terminal amyloid precursor protein fragments (Kametani and Haga, 2015). This AD-related increase in PDE8B expression may contribute to cognitive deficits associated with the disease because PDE8B inactivation in rodents strengthens recent long-term memory for hippocampus-dependent memories (Tsai et al, 2012). Furthermore, recently characterized PDE8 inhibitors demonstrated therapeutic effects in mouse models of vascular dementia (Huang et al, 2020c; Wu et al, 2022). Together, these results suggest PDE8B inhibitors may be a therapeutic approach for cognitive decline; however, this potential may be limited by anxiogenic side effects (Tsai et al, 2012).

##### i PDE9

Multiple PDE9A isoforms also exhibit age-related changes in expression in a brain region-specific manner (Patel et al, 2018). In particular, PDE9A isoforms decreased during early postnatal development in the cerebellum and hippocampus of rodents (Patel et al, 2018). Importantly, PDE9A mRNA also decreases during early life in the human hippocampus (Patel et al, 2018). This age-related decrease in hippocampal PDE9A mRNA may reflect a healthy adaptive process because hippocampal PDE9A mRNA is synergistically elevated in the hippocampus of individuals with a history of traumatic brain injury plus dementia relative to controls, although patients with only traumatic brain injury or dementia showed no change relative to controls (Patel et al, 2018). The lack of change in PDE9A expression in dementia-only patients may help explain why the PDE9 inhibitors PF-04447943 and BI-409306 failed to improve either cognition or dementia-related behavioral disturbances in patients with AD in phase II clinical trials (Schwam et al, 2014; Frolich et al, 2019). These clinical failures stood in the face of preclinical studies showing that PDE9A inhibitors rescue cytotoxicity, plasticity impairments, and memory deficits in AD rodent models (Kroker et al, 2014; Li et al, 2016a; Rosenbrock et al, 2019). Furthermore, the development of novel PDE9 inhibitors continues to be pursued for neurodegeneration (Zhang et al, 2020b; Ribaudo et al, 2021; Swetha et al, 2022).

Not only do expression levels of PDE9A isoforms change with age in a brain region-specific manner but so does their subcellular localization (Patel et al, 2018). For example, across early development PDE9A6/13 and PDE9X-120 shift from the membrane to the nucleus in the prefrontal cortex and cerebellum but not the striatum or hippocampus (Patel et al, 2018). As discussed elsewhere (Salpietro et al, 2018), the issue of subcellular localization may also have contributed to the aforementioned clinical failures of PF-04447943 and BI-409306 for AD. That is, PDE9A is enriched in the nucleus and membrane (Patel et al, 2018) and, thus, is not in a position to directly regulate the cytosolic pools of cGMP that appear to be dysregulated in AD (Bonkale et al, 1995; Baltrons et al, 2002; Baltrons et al, 2004).

##### j PDE10

Prenatally, PDE10A mRNA is widely expressed throughout human cortical regions, hippocampus, amygdala, and striatum; however, PDE10A levels dramatically drop by childhood in all regions except in the striatum (Farmer et al, 2020). In the adult human brain, then, PDE10A is predominantly expressed in striatal medium spiny neurons (Geerts et al, 2017; Farmer et al, 2020) and has been largely studied in the context of corticostriatal disorders such as HD and schizophrenia (cf, Baillie et al, 2019). In adult rodents, PDE10A mRNA is also predominantly expressed in the striatum; however, it is also found at very low levels in the cortex, cerebellum, and hippocampus (Kelly et al, 2014; Farmer et al, 2020). Even so, PDE10A deletion or inhibition in rodents has largely proven ineffective, if not harmful, for learning and memory (Siuciak et al, 2006a,b; Sano et al, 2008; Schmidt et al, 2008; Siuciak et al, 2008b). PDE10A is widely reported to be downregulated in the striatum of patients with HD, with the extent of PDE10A loss corresponding to the number of CAG repeats within the Huntington gene (Hebb et al, 2004; Ahmad et al, 2014; Russell et al, 2014; Russell et al, 2016; Wilson et al, 2016; Fazio et al, 2020). PDE10A mutations linked to hyperkinetic movement disorders that phenocopy many features of HD reduce PDE10A expression due to irregular subcellular trafficking that leads to increased PDE10A degradation in the cytosol (Tejeda et al, 2020). Experimentation using highly specific, PDE10A positron emission tomography tracers shows PDE10A expression continues to decline over the years, suggesting the enzyme could be a useful biomarker for assessing the initial diagnosis and subsequent progression of HD (Russell et al, 2016). This downregulation of PDE10A in the HD brain may reflect a compensatory mechanism aimed at increasing cAMP/cGMP signaling, which is known to be reduced in patients’ samples (Gines et al, 2003). Indeed, HD mouse models also show reduced striatal PDE10A expression (Hebb et al, 2004; Hu et al, 2004; Leuti et al, 2013; Miller et al, 2014; Beaumont et al, 2016); however, PDE10 inhibitors rescue behavioral, neurodegenerative, and electrophysiological deficits (Giampa et al, 2009; Giampa et al, 2010; Giralt et al, 2013; Beaumont et al, 2016; Harada et al, 2017). That said, Pfizer’s PF-02545920 failed to improve symptoms in patients with HD and, therefore, further development was terminated (cf, Baillie et al, 2019). Omeros and Palobiofarma similarly explored HD as an indication for their PDE10 inhibitors; however, those efforts were suspended or have been terminated (cf, Baillie et al, 2019).

##### k PDE11A

In the rodent brain, PDE11A is quite unique in that it is the only PDE whose mRNA expression emanates predominantly (if not solely) from the hippocampus (Kelly et al, 2014). Age-related increases in PDE11A mRNA and PDE11A4 protein expressions have been reported in the mouse, rat, and human hippocampus (Kelly et al, 2014; Pilarzyk et al, 2022). Interestingly, these age-related increases in protein expression are driven, at least in part, by phosphorylation of Ser^117^ and Ser^124^ in the PDE11A4 *N*-terminal regulatory domain, which also triggers the protein to ectopically accumulate within filamentous structures termed ghost axons (Pilarzyk et al, 2022; Pilarzyk et al, 2023). These age-related increases in PDE11A4 expression are likely a direct contributor to the age-related increases in hippocampal PDE hydrolytic activity and decreases in CREB function described above (Kelly et al, 2010; Smith et al, 2021; Pilarzyk et al, 2022) as well as age-related decreases in expression of the NR1 subunit of the N-methyl-D-aspartate (NMDA) receptor that occur post-synaptically in the prefrontal cortex (Pilarzyk et al, 2019; McQuail et al, 2021). These age-related increases in hippocampal PDE11A4 protein expression may also contribute to age-related changes in microglia activation and cytokine expression in the hippocampus (Pathak et al, 2017; Pilarzyk et al, 2021; Porcher et al, 2021).

Genetic deletion of PDE11A in mice leads to a transient amnesia for social memories that ultimately produces stronger, remote long-term social memories in young adult mice and prevents the age-related cognitive decline of remote long-term social memories in old mice (Pilarzyk et al, 2019; Pilarzyk et al, 2022). This transient amnesia correlates with changes in the overall activation levels and functional connectivity of frontal cortical regions and hippocampal/parahippocampal regions and reduced expression of the glutamate receptor NR1 subunit in the prefrontal cortex (Pilarzyk et al, 2019). Furthermore, viral restoration of PDE11A4 selectively to ventral CA1 of adult *Pde11a* KO adult mice was sufficient to reverse the memory phenotypes caused by the deletion, suggesting that the nootropic effect of the deletion was due to the acute loss of PDE11A4 signaling in the adult brain as opposed to an effect on development (Pilarzyk et al, 2019; Pilarzyk et al, 2022). Based on these findings, potent and selective PDE11 inhibitors are currently being developed for treating age-related cognitive decline (Mahmood et al, 2023).

Disrupting PDE homodimerization may also prove to be an effective way to target PDE11A4 function in a subcellular domain-specific manner (GAF-B domain) to rescue cognitive deficits (Pathak et al, 2017). Interestingly, age-related increases in ventral hippocampal PDE11A4 protein are localized to the membrane (Pilarzyk et al, 2022), which suggests the isolated GAF-B domain might prove quite beneficial in the context of age-related cognitive decline (Pilarzyk et al, 2022). Indeed, viral expression of the isolated GAF-B domain in CA1 of mouse hippocampus was able to reduce PDE11A4 protein expression in a compartment-specific manner, reverse the age-related cognitive decline of remote long-term social associative memory, and improve social recognition memory in old mice, albeit at the expense of being unable to access recent long-term social memories (Pilarzyk et al, 2023).

##### l Challenges and outlooks

As discussed elsewhere (Baillie et al, 2019), this is clearly an exciting time in the PDE field, but there is much work that remains to be done. For therapeutics to be efficiently developed, we need to have a more thorough understanding of exactly where cyclic nucleotide signaling is disrupted in a given disease, and in which tissue, cell types, and subcellular compartments. We then need to target a PDE in a defined locale, with the understanding that subcellular compartmentalization of a given PDE may vary depending on species, age, tissue type, or disease status (Houslay and Baillie, 2005; Huston et al, 2006; Nagel et al, 2006; Richter et al, 2008; Ahmad et al, 2009; Houslay, 2010; Penmatsa et al, 2010; Al-Tawashi and Gehring, 2013; Perera et al, 2015; Patel et al, 2018). This consideration is equally important in the evaluation of potential efficacy and potential side effects. To maximize potential efficacy while minimizing potential side effects, 1 would target a PDE that is enriched, if not exclusively expressed, in the tissue of interest and that controls the same pool of cyclic nucleotide that is altered by the disease. At the same time, efforts to unravel the intramolecular signals responsible for trafficking each PDE also need to continue to inform more sophisticated therapeutic approaches that can preferentially target a given PDE in a given subcellular compartment. Along these same lines, we need to grow our understanding of how to stimulate PDE activity and how to target the PDE catalytic activity of dual-specificity PDEs in a functionally selective manner (ie, target only its cAMP- or cGMP-hydrolytic activity, [see Kelly, 2015] for further discussion). Perhaps by increasing the specificity of our approach, we can retain efficacy while mitigating the numerous side effects described above that have plagued PDE inhibitors to date.

##### m PDE isoforms, dopamine signaling, and disease: implications for treatment

There is a high degree of overlap between the expression of PDE isoforms and dopamine signaling pathways that mediate motor movement and motivated behaviors. Moreover, the use of new genetic models and novel pharmacological inhibitors has shown that many of the prominent brain PDEs directly impact cyclic nucleotide-dependent dopamine signaling pathways in a region-specific and cell type-specific manner. Certain PDE isoforms, then, represent targets for novel pharmaceutical approaches to a wide array of neuropsychiatric and neurodegenerative diseases that affect dopamine neurons and their target cells, including schizophrenia, Parkinson’s disease (PD), HD, and depression. Although the role of PDE isoforms in brain and behavioral disease is the subject of other contributions to this review, we review briefly, here, the potential utility of PDE inhibitors for the treatment of dopamine-related neurodegenerative disease, PD.

*Parkinson’s disease and symptomatic treatment*. PD is characterized as a progressive degeneration of dopamine (DA)-containing neurons in the brain. It is most often characterized by motor deficits, notably bradykinesia, limb rigidity, and resting tremor. Dopamine depletion—most notable as the degeneration of nigrostriatal dopamine neurons—is considered the primary cause of the loss of volitional movement in PD. This effect may contribute to the associated nonmotor symptoms of the disease, including cognitive dysfunction, loss of effect, and depression (Hornykiewicz, 1966; Bernheimer et al, 1973), although nondopaminergic systems are also clearly involved (Jellinger, 1991; Braak et al, 2003). Mutations in specific genes, including *LRRK2*, synuclein, and *PINK1*, are linked to familial forms of PD (Biskup et al, 2008) but may also play important roles in many cases (ie, >95 % of patients) of the disease for which the biological cause is unknown. There is no cure for PD. Rather, the disease is currently addressed with symptomatic treatments that temporarily restore motor function, including replacement therapies like L-dihydroxyphenylalanine (L-DOPA or levodopa), the immediate precursor for DA synthesis (Jankovic and Aguilar, 2008). Unfortunately, long-term L-DOPA therapy becomes ineffective in treating motor disabilities with chronic use.

The use of adjunctive or alternate first-line therapies that delay the introduction of L-DOPA therapy or reduce the required dose of L-DOPA can positively affect the course of the disease and the appearance of motor side effects (Schrag and Quinn, 2000). Dopamine receptor agonists (eg, pramipexole, ropinirole) may slow disease progression (Olanow, 2009) and have become part of the arsenal for the treatment of early stage disease, perhaps with fewer drug-induced dyskinesias (Rascol et al, 2000). Interestingly, in vitro preclinical studies show that dopamine receptor agonists may exert antiapoptotic effects directly on dopamine neurons (eg, via autoreceptors; Olanow, 2009); furthermore, dopamine receptor agonists may act at post-synaptic sites (downstream of actions on dopamine neurons) to normalize dopamine activity within the basal ganglia motor system and slow disease progression. The strategy of normalizing motor symptoms to slow disease progression by intervening at points distant from the affected dopamine neurons is the basis for several novel therapeutic approaches.

At present, the only medications recognized by the FDA for efficacy in the treatment of LIDs are Istradefylline, an A2A adenosine receptor antagonist (Cummins and Cates, 2022) and amantadine (Metman et al, 1999), a mixed-action drug that produces a modest attenuation of LIDs in some patients via molecular mechanisms that are unclear, but which likely involve NMDA receptor blockade and dopamine agonist activities (Jankovic and Aguilar, 2008). Based on these data, a major avenue for the development of new PD therapies is the discovery of stand-alone or adjunctive therapies that will increase “on” time, enable replacement of L-DOPA or lower maintenance doses of L-DOPA, and prolong the useful lifetime of PD therapy, delaying or preventing the appearance of motor fluctuations, including LIDs.

*PDEs as Novel Targets for PD Therapy:* PDEs are exciting and novel targets for the development of new therapies to treat the symptoms (motor and non-motor symptoms), motor side effects, and possibly to modify disease progression in PD. The interest in these enzymes stems, in part, from their abundant expression in brain regions that sustain a loss of motor function in PD (eg, basal ganglia) or regions that subsume important roles in cognition (eg, the prefrontal and dorsolateral cortex and hippocampus)—a prominent non-motor symptomatic deficit in PD (Lakics et al, 2010). In addition, the ability of PDEs to control levels of the second messengers, cAMP and cGMP, which are the key signaling molecules in the actions of dopamine on motor and cognitive function (Greengard et al, 1999), is another appealing functional property. Although it is unclear whether modulation of cAMP or cGMP might be differentially beneficial in addressing symptoms and progression in PD, we will here focus on 4 PDE families with possible benefit for PD)—2 which are cAMP-preferring in their actions (PDE4 and PDE7A/B), and 2 which hydrolyze both cAMP and cGMP (PDE10A and PDE1).

*Inhibitors of cAMP-Preferring PDE4 Enzymes in PD.* PDE4 enzymes have been proposed as drug targets of interest for PD as family members, including PDE4B, are abundantly expressed in nigrostriatal dopamine terminals and in striatal medium spiny neurons (MSNs), in proximity to presynaptic and postsynaptic dopamine signaling machinery (Yamashita et al, 1997). Pharmacological inhibition of PDE4 with rolipram increases dopamine synthesis in cultured mesencephalic dopamine neurons (Yamashita et al, 1997). This effect is consistent with the ability of PDE4 inhibition to increase cAMP levels in these neurons, leading to phosphorylation of the dopamine synthetic enzymes, tyrosine hydroxylase (TH) at a site (Ser^40^) that catalyzes dopamine synthesis. These data are supported by in vivo studies demonstrating increases in TH phosphorylation and dopamine turnover in the striatum in response to rolipram (Nishi et al, 2008). PDE4 inhibitors also exhibit antidepressant and procognitive effects in a variety of animal models (Bolger et al, 1994; Barad et al, 1998; Bourtchouladze et al, 1998; D'Sa et al, 2005; Takahashi et al, 1999; Zhang et al, 2009) that would address nonmotor symptoms of PD that are poorly responsive to L-DOPA pharmacotherapy (Chaudhuri and Schapira, 2009). The ability of PDE4 inhibitors to drive dopamine synthesis and release and provide nonmotor support, suggesting that they might be useful in addressing early stage PD.

The positive pharmacological effects of PDE4 inhibitors, however, are complicated by postsynaptic effects that mimic the actions of dopamine D_2_-receptor antagonists. D_2_-receptor blocking drugs, such as the antipsychotic medication, haloperidol, produce motor disturbances in animals that resemble extrapyramidal motor symptoms and tardive dyskinesia (Klawans and Weiner, 1974). Dopamine receptor antagonists can interfere with the restoration of motor activity by L-DOPA in animal models of PD (Boyce et al, 1990; Grondin et al, 1999). The PDE4 inhibitor, rolipram, preferentially increases DARPP-32 phosphorylation at Thr^34^ in striatopallidal neurons (Nishi et al, 2008), which are predominantly controlled by dopamine D_2_ receptors. Thus, PDE4 inhibitors produce an effect in this subset of striatal neurons characteristic of dopamine D_2_-receptor antagonists such as haloperidol, which is shared with PDE10A inhibitors (see below), such as papaverine (Siuciak et al, 2006a). Overall, inhibition of PDE4 enzymes modestly reduces motor activity in normal mice and rats and potentiates the catalepsy produced by neuroleptic drugs (Kanes et al, 2007; Siuciak et al, 2007a). Thus, despite the favorable potential effects of PDE4 inhibitors for non-motor symptoms of PD, including treatment of cognitive deficits and depression (Zhang, 2009), it is unlikely that the motor effects of pan-PDE4 inhibitors would be tolerated in PD patients.

Perhaps the greatest limitation to the development of PDE4 inhibitors for PD, and for CNS-based disorders in general, are the associated gastrointestinal and emetic side effects. The emetic response to PDE4 inhibitors has been attributed to the inhibition of the PDE4D isoform in the brain (Robichaud et al, 2002; Mori et al, 2010); indeed, PDE4D expression is enriched in the area postrema, a region controlling the emetic response. Emesis limits the tolerability of PDE4 inhibitors, thus stalling their development for brain disorders. Peripherally restricted inhibitors of PDE4 isoforms, including roflumilast and apremilast, have been successfully developed and approved by the FDA for the treatment of peripheral inflammatory disorders such as COPD; roflumilast has also been investigated for CNS disorders (Prickaerts et al, 2017), albeit with a narrow therapeutic window. Efforts continue toward the design of PDE4 inhibitors that minimize PDE4D-related safety concerns. These efforts have focused on the design of compounds (eg, zatolmilast) that work as negative allosteric inhibitors of the PDE4D enzyme and, thereby, lack full emetic potential. To this end, Tetra Therapeutics has advanced a PDE4D inhibitor, zatolmilast, into phase III clinical development for the treatment of Fragile X syndrome (NCT05163808).

*Anti-inflammatory and neuroprotective potential of cAMP-preferring PDE4 and PDE7 inhibitors*. Another area of drug development focus has been the design of PDE4 inhibitors that target, selectively, the PDE4B isoform, which is proposed not to regulate the emetic response (Fox 3rd et al, 2014). PDE4B-preferring inhibitors have been discovered and tested preclinically for CNS activity (Pearse and Hughes, 2016) and for safety in assays thought to predict emetic potential in humans. Inhibitors (eg, ABI-4) of the brain-enriched PDE4B isoform exert strong anti-inflammatory and neuroprotective actions in cell-based assays. For example, the release of TNF*α* from LPS-stimulated human PBMCs and murine primary microglia was suppressed by ABI-4 in a concentration-dependent manner (Hedde et al, 2017). Similarly, ABI-4 suppressed the brain and plasma levels of proinflammatory cytokines, including IL1*β* and IL-6 (although not TNF*α*) in mice in vivo (Hedde et al, 2017). Aging has been associated with enhanced systemic inflammation (Franceschi et al, 2007); subchronic administration of AB4-1 in aged mice significantly reduced brain levels of TNF*α* and IL1*β*. Furthermore, lower levels of plasma TNF*α* were observed in mice genetically lacking the PDE4B isoform (Hedde et al, 2017). Despite the promising preclinical effects of more selective PDE4B inhibitors, like ABI-4 in inflammation and aging models, a suitable PDE4 inhibitor is yet to be evaluated clinically for the treatment of motor and/or nonmotor symptoms of PD.

The anti-inflammatory effects of PDE4B-preferring compounds are particularly interesting with regard to PD as it has become increasingly evident that neuroinflammation and immune system dysfunction are likely to be causative or exacerbating factors in the symptomatology and progression of PD (Tansey et al, 2022). As discussed above, PDE4 inhibitors, including PDE4B-preferring molecules, are reported to have potent anti-inflammatory actions that might protect neurons in models of PD and other neurodegenerative diseases. This property of PDE enzymes will be discussed below in reference to other PDE families, including those for PDE7, PDE10A, and PDE1.

*Inhibitors of cAMP-Preferring PDE7A/B Isoforms in PD*. The PDE7 family has also been proposed as a potential target for PD therapy due, in part, to high expression in striatal neurons and the potent anti-inflammatory/neuroprotective effects of inhibitors. To date, only a few studies have been published on this cAMP-preferring PDE. PDE7 enzymes (predominantly the PDE7B isoform) are abundantly expressed in the brain. PDE7B mRNA levels are high in rat dentate gyrus, striatum, and olfactory tubercle (Reyes-Irisarri et al, 2005). PDE7B mRNA is localized to striatal MSNs and its translational regulation under the control of the dopamine D_1_-receptor is confirmed (Sasaki et al, 2004). More recently, double *in situ* hybridization analysis has shown that the PDE7B signal also localizes to dopamine D_2_-receptor-containing striatal neurons (De Gortari and Mengod, 2010), further supporting the potential significance of this PDE as a target for the development of therapies for PD. Expression of PDE7B in the hippocampus further connects this isoform with brain circuitry underlying cognitive dysfunction in PD. A link between familial PD genes and PDE7B is supported by a recent study in mice overexpressing mutant A53T-alpha (*α*) synuclein (Kurz et al, 2010). Mutant *α*-synuclein, which was associated with reduced levels of several indices of striatal dopamine signaling, was found to negatively regulate striatal gene expression, including the gene encoding PDE7B. High-affinity inhibitors of this PDE (eg, OMS182401) are being investigated for motor benefit in animal models of PD (see http://www.michaeljfox.org/).

PDE7 inhibitors, like PDE4B inhibitors, elicit strong anti-inflammatory effects and may be responsible for protective effects on dopamine neurons (Garcia et al, 2014; Chen and Yan, 2021; Zorn and Baillie, 2023). Inhibition of PDE7B or silencing of the gene encoding PDE7B dampens expression of inflammatory cytokines (like TNF*α*) in rodent neurons treated with 6-OHDA (a dopamine-depleting neurotoxin) or the inflammogen, LPS (Chen et al, 2021). The neuroprotective effects of PDE7 inhibitors are accompanied by increases in tissue levels of cAMP, suggesting that they protect dopamine neurons via pathways involving cAMP (Sasaki et al, 2004). The PDE7A isoform, furthermore, is highly expressed in human proinflammatory and immune cells (Smith et al, 2004), providing a broader role for this PDE in the regulation of brain and systemic inflammation.

*Inhibitors of the Dual cAMP/cGMP PDE10A Enzyme in PD*. A high level of basic research and drug development interest has focused on PDE10A, one of the dual cAMP/cGMP hydrolyzing PDE families. Initial interest in PDE10A inhibitors was as a novel target for the treatment of schizophrenia (Schmidt et al, 2009). PDE10A inhibitors, including the tool compound, papaverine, mimic the effects of established antipsychotic medications possessing dopamine D_2_-receptor antagonist activity in a variety of behavioral models (Siuciak et al, 2006a, 2007a). Deletion of the gene encoding *PDE10A* mimicked the behavioral actions of antipsychotic medications in mice (Siuciak et al, 2006a). These studies, however, noted that pharmacological inhibition of PDE10A or deletion of the *PDE10A* gene consistently elicited disruptions in motor function, including reduced spontaneous locomotor activity and increases in response latency in sensorimotor tests (Siuciak et al, 2006a). Clinical investigations of PDE10A inhibitors established a lack of efficacy in the treatment of psychosis (Walling et al, 2019; Menniti et al, 2021).

Despite concerns that dopamine D_2_-receptor antagonist-like properties of PDE10A inhibitors could further compromise motor activity, mild dopamine D_2_ antagonist activity provided by these agents might be of value in the management of LIDs. Dopamine receptor sensitization that results from the loss of striatal dopamine innervation and the effects of dopamine replacement therapy likely contribute significantly to the development of LIDs (Nutt, 1990). Thus, mild dopamine D_2_-receptor antagonist-like activity that would normalize dopamine receptor responses to L-DOPA might delay the onset or lessen the severity of motor responses to replacement therapy. Dopamine D_2_-receptor antagonists effectively suppress the appearance of specific behaviors in animals that are analogous to human dyskinesias, including axial, limb, and orolingual movements, without significantly comprising L-DOPA effects on spontaneous motor activity (Monville et al, 2005; Taylor et al, 2005). Antagonists of specific receptors within the D_2_ family, like the D_3_-type dopamine receptor, have recently been shown to suppress LIDs, supporting the idea that molecules with D_2_-receptor antagonist-like activity may be useful anti-dyskinetic agents (Visanji et al, 2009). Clinically, several studies support the efficacy of atypical antipsychotic drugs, such as clozapine and aripiprazole, for the control of LIDs. The positive effects of these drugs may be due to their complex pharmacology, which includes activity at several different receptors. Thus, the interesting dopamine signaling effects of both PDE4 and PDE10A inhibitors may warrant their consideration for neurological indications such as LIDs. For example, abnormal orolingual movements induced by chronic neuroleptic drug treatment, a model for dyskinetic behaviors seen after L-DOPA, is attenuated by rolipram treatment (Sasaki et al, 1995).

*Inhibitors of the Dual cAMP/cGMP PDE1 Enzyme in PD*. PDE1 enzyme also represents an interesting target for addressing the motor symptoms of PD. The concept is supported by the enrichment of the PDE1B isoform in basal ganglia (Polli and Kincaid, 1994), and the demonstrated actions of pan-PDE1 inhibitors in enhancing cAMP-dependent actions of dopamine at the biochemical and behavioral level (Snyder et al, 2016; Pekcec et al, 2018). PDE1B was originally recognized as an attractive candidate for dopamine-related indications such as PD based on its striatal enrichment and close association with brain regions receiving heaving dopaminergic innervation (Polli and Kincaid, 1994; Yan et al, 1994). Furthermore, *PDE1B* gene knockout amplifies dopamine signaling via D_1_-receptor pathways and motor activity stimulated by low-level dopamine agonist administration (Reed et al, 2002; Ehrman et al, 2006; Siuciak et al, 2007a). The data support the idea that PDE1B inhibition enhances dopamine signaling in a stimulus-bound manner, as deletion of the *PDE1B* gene in mice resulted in no significant change in basal protein phosphorylation and negligible changes in basal locomotor activity. This quality is consistent with the unique regulatory properties of the PDE1 family of enzymes. As all 3 PDE1 family members are stimulated by Ca^2+^/CaM (the only 1 of 11 PDE families with this property), their activity is likely controlled by neuronal activity. This property confers an “on-demand” quality to PDE1 activity that would be anticipated to provide phasic amplification of dopamine signaling contingent upon stimulation of MSNs by endogenous factors. The “on-demand” activity of PDE1B may be a superior attribute as a drug target compared with dopamine agonists which tonically activate dopamine receptors (Wennogle et al, 2017). The tonic activation of receptors by agonists is 1 factor, which results in dopamine receptor changes that contribute to the development of motor fluctuations (Olanow, 2009). Establishing PDE1B as a therapeutic target for PD will need to be evaluated with potent and selective inhibitors.

Over the past decade, several potent and selective PDE1 inhibitors have been discovered and reported to have activity on motor features of PD and/or on behavioral dimensions such as cognition, which are prominent non-motor features compromised in PD and poorly treated by current PD medications. The first fully characterized, orally active, brain permeant, and selective inhibitor of PDE1 was Lenrispodun. Lenrispodun has been reported, primarily, to enhance memory performance in rodents using the novel object recognition test (Snyder et al, 2016). Pekcec and colleagues further demonstrated the ability of the compound to elevate brain levels of cAMP and cGMP and facilitate dopamine D_1_-receptor and PKA-dependent neural transmission in prefrontal cortical brain slices (Pekcec et al, 2018). Behaviorally, these investigators showed that the compound acted like a dopamine D_1_-receptor agonist to reverse MK-801-induced cognitive deficits in a continuous alternation task. Interestingly, the compound preferentially improved the cognitive performance of “low performing” rats in the 5-CSRTT attentional assay, although having little effect on “high performing” rats. These results fit nicely with, what is referred to, as an “inverted U” curve noted with cognitive responses of animals treated with dopamine D_1_-receptor agonists. Animals typically show improved cognitive performance in response to dopamine D_1_-receptor agonists only at optimal levels of dopamine D_1_-receptor agonism; lower and higher levels of activity are associated with poorer cognitive performance (Goldman-Rakic et al, 2000).

Most recently, a novel inhibitor of PDE1 has been disclosed by Sumitomo Dianippon Pharma Co., Ltd, which has efficacy in reducing the expression of dyskinetic behavior in MPTP-lesioned primates receiving long-term treatment with L-DOPA (Enomoto et al, 2021). To date, it is unclear whether this PDE1 inhibitor is being advanced into clinical testing for PD or any other indications.

It is noteworthy that like inhibitors of PDE4, PDE7, and PDE10A, PDE1 inhibitors also possess potent anti-inflammatory activity in cell-based and in vivo inflammation models. Early on, the PDE1B isoform was found to be expressed in immune cells. Bender and Beavo (2006) demonstrated enhanced expression of PDE1B levels in monocytes when stimulated to differentiate into macrophages. Another study found that the pan PDE1 inhibitor, Lenrispodun, suppressed the release of the proinflammatory cytokine, TNF*α*, from microglia-like BV2 cells in response to LPS treatment (O'Brien et al, 2020). Lenrispodun also inhibited the expression of proinflammatory genes in LPS-stimulated BV2 cells, including IL1*β* and CCL2. Functionally, these gene expression changes were correlated with the inhibition of BV2 migration toward the chemoattractant, ADP, in a Boyden chamber assay, implying that the compound was capable of dampening the recruitment of microglia to sites of inflammation (O'Brien et al, 2020). Transcriptome analysis using RNAseq showed that most genes regulated by Lenrispodun in LPS-treated cells were distinct from those regulated by rolipram, a paninhibitor of PDE4; PDE4 is the nearest cross-reactive PDE family to Lenrispodun (Li et al, 2016b; Snyder et al, 2016). These data support the idea that inhibitors of the PDE1 family of enzymes appear able to distinctly regulate unique gene networks separate from those controlled by a well characterized pan-PDE4 inhibitor (O'Brien et al, 2020). Whether PDE7 and PDE10A inhibitors also control networks of genes distinct from PDE1 and other PDE families has yet to be clarified. Taken together, the prominent anti-inflammatory effects of PDE1 inhibitors argue for possible neuroprotective effects in PD in addition to possible motor-based symptomatic effects.

*cGMP and corticostriatal correlates of dyskinesia: a possible role of PDE inhibitors.* The therapeutic potential of PDE inhibitors and, in particular, inhibitors of cGMP hydrolysis catalyzed by dual-specificity PDEs, in PD is highlighted by recent research on the molecular basis of dyskinesia. These studies have identified a dysfunction in striatal cGMP signaling as an electrophysiological correlate of LIDs in animals. Work by Calabresi and colleagues has identified a deficit in long-term depression (LTD) of striatal responses to high-frequency stimulation (HFS) of corticostriatal slices in dopamine-depleted animals displaying dyskinesia after chronic L-DOPA treatment (Picconi et al, 2003). Rats depleted of striatal dopamine with the neurotoxin 6-OHDA received repeated daily doses of L-DOPA, which resulted in a subset of animals developing LIDs and another subset of rats that remained nondyskinetic under similar treatment conditions. Corticostriatal slices from dyskinetic rats were distinguishable from those from non-dyskinetic animals based on the loss of LTD responses. Thus, the absence of LTD provides an electrophysiological correlate for dyskinesia (Picconi et al, 2003). Rats with established LIDs expressed lower striatal levels of cAMP and cGMP compared with normal animals. Treatment of these animals with agents such as zaprinast, an inhibitor of cGMP preferring PDEs, partially restored striatal cyclic nucleotides and attenuated dyskinesias (Giorgi et al, 2008). Zaprinast, and other cGMP-elevating agents, further induced LTD responses in corticostriatal slices (Calabresi et al, 1999; Picconi et al, 2011). Together, these data indicate that LIDs may be associated with abnormal corticostriatal plasticity that results from deficits in cGMP levels. Thus, PDE inhibitors that elevate striatal cGMP levels and signaling are potentially beneficial for attenuating LIDs. These data support a possible therapeutic role for inhibitors of several striatal-enriched PDEs in this indication including PDE1B.

### F Cancer and inflammation

#### 1 General framework of cyclic nucleotide signaling in cancer

Several generations of research in the biology of cancer have led to the definition of a number of properties that collectively define the neoplastic state, commonly referred to as the Hallmarks of Cancer (Hanahan and Weinberg, 2000, 2011). The original definition of these hallmarks included 6 biological capabilities acquired during the multistep development of human cancer. These included: (1) sustaining proliferative signaling, (2) evading growth suppressors, (3) resisting cell death, (4) enabling replicative immortality, (5) inducing angiogenesis, and (6) activating invasion and metastasis. Essential for the acquisition of these hallmarks is the concept of genome instability, which contributes to the mutations required for the development of neoplasia, and inflammation, which promotes several hallmark functions. To these 6 hallmarks, 2 more were subsequently added: (7) reprogramming of energy metabolism and (8) evading immune destruction. Study of cyclic nucleotide signaling in cancer provides an opportunity to observe these hallmarks in action. Alterations in cAMP and cGMP signaling can profoundly contribute to the neoplastic state. Modulation of cAMP and, especially, cGMP signaling can profoundly attenuate and modulate the cancer phenotype. Collectively, these advances in cyclic nucleotide signaling in neoplasia have the potential to lead to important advances in the prevention, diagnosis, and treatment of several important human cancers.

#### 2 Effects in neoplastic cells

##### a cAMP PDE: a role for driver mutations

The concept of driver mutations is essential to understanding the functional, pathogenic, and clinical role of cAMP and cGMP signaling in cancer. Driver mutations are defined as germline or somatic mutations in DNA that play an essential role in generating the transformed phenotype (Greenman et al, 2007; Bailey et al, 2018; Sack et al, 2018; Sanchez-Vega et al, 2018; Gorelick et al, 2020). Driver mutations can therefore be defined as follows: (1) they materially affect the expression or structure of the RNA/protein encoded by the mutated gene(s), leading to alterations in its physiological function(s), producing a growth advantage; (2) they localize to specific “hot spots” within the gene product essential to its cellular function, such as its enzymatic activity or regulation; and (3) they are present in a substantial proportion of clinical specimens obtained from a specific cancer type. “Passenger” mutations, in contrast, differ from driver mutations in that they do not play a clear role in cancer formation. Passenger mutations typically (1) do not change the physiological or biochemical functions of the gene product; (2) do not concentrate in “hot spots”; and (3) are found in only a small proportion of clinical specimens obtained from a specific cancer type.

Using the strict criteria for cancer driver mutations, as defined above, we can identify cancer-associated driver mutations in 11 different genes encoding members of cAMP-signaling pathways, which are involved in at least 9 different cancers (Table 1) [see (Ahmed et al, 2022; Bolger, 2022) for recent reviews]. In the PDE space, germline and tumor-associated driver mutations in *PDE8B* and *PDE11A* are the best-characterized driver mutations, as described in detail previously (Bolger, 2022). The *PDE8B* mutations have been identified in patients with adrenal hyperplasia, adenomas, and carcinomas and have been shown to attenuate PDE8 enzymatic activity. Other PDE germline mutations may also predispose to adrenal tumors (Bolger, 2022).

**Table 1:** Human cancers with driver mutations in genes that encode elements of cAMP-signaling pathways

Tissue	Cancer Type	Pathway Element	Gene Name
Adrenal cortex	Adenoma	G protein alpha subunit	*GNAS*
		Phosphodiesterase	*PDE8B*
		Phosphodiesterase	*PDE11A*
		PKA regulatory subunit	*PRKARA1*
		PKA catalytic subunit	*PRKACA*
		PKA catalytic subunit	*PRKACB*
Thyroid	Adenoma	GPCR	*TSHR*
Parathyroid	Adenoma	G protein alpha subunit	*GNAS*
Pituitary	Somatotropinoma	GPCR	*GPR101*
		PKA regulatory subunit	*PRKARA1*
Testis Sertoli cell	LCCSCT	PKA regulatory subunit	*PRKARA1*
Testis Leydig/Sertoli cell	Germ cell tumors	Phosphodiesterase	*PDE11A*
Testis germ cell	Germ cell tumors	Phosphodiesterase	*PDE11A*
Liver	Fibrolamellar HCC	PKA catalytic subunit	*PRKACA*
Abdominal soft tissue and CNS	FET-CREB fusion tumors	Transcription factor	*CREB*

HCC, hepatocellular cancer; LCCSCT, large-cell calcifying Sertoli cell tumors.

As thoroughly reviewed elsewhere (Kelly, 2015, 2018b), a large number of PDE11A intronic, synonymous (ie, noncoding), missense (ie, nonsynonymous coding), and nonsense mutations (ie, truncating) have been associated with a variety of endocrine-related tumors. The majority of these variants are common, but rare mutations have also been reported in a small number of patients with various tumor types (Kelly, 2018b). The tumor-associated PDE11A mutations largely produce a loss-of-function phenotype either by reducing expression, catalytic activity, or proper localization of the enzyme; however, it is important to note that the effects of the mutations are often cell-type specific (Horvath et al, 2006; Libe et al, 2008; Peverelli et al, 2009; Horvath et al, 2009; Libe et al, 2011; Faucz et al, 2011; Kelly, 2015; 2018b; Pathak et al, 2015; Vezzosi et al, 2012). Tumors of endocrine tissues have also been found to be associated with reduced PDE11A expression of non-mutational genetic causes, such as transcriptional and epigenetic regulation (Boikos et al, 2008; Mirabello et al, 2012). Together, these studies suggest that a loss of PDE11A function is more likely a risk modifier than an inducer of tumors (Kelly, 2015), which is supported by PDE11A KO mouse studies that show no increased presence of tumors (Kelly et al, 2010).

##### b cGMP PDE: a role of PDE5 and PDE10 in cell cycle regulation and apoptosis

**Fig. 16:**
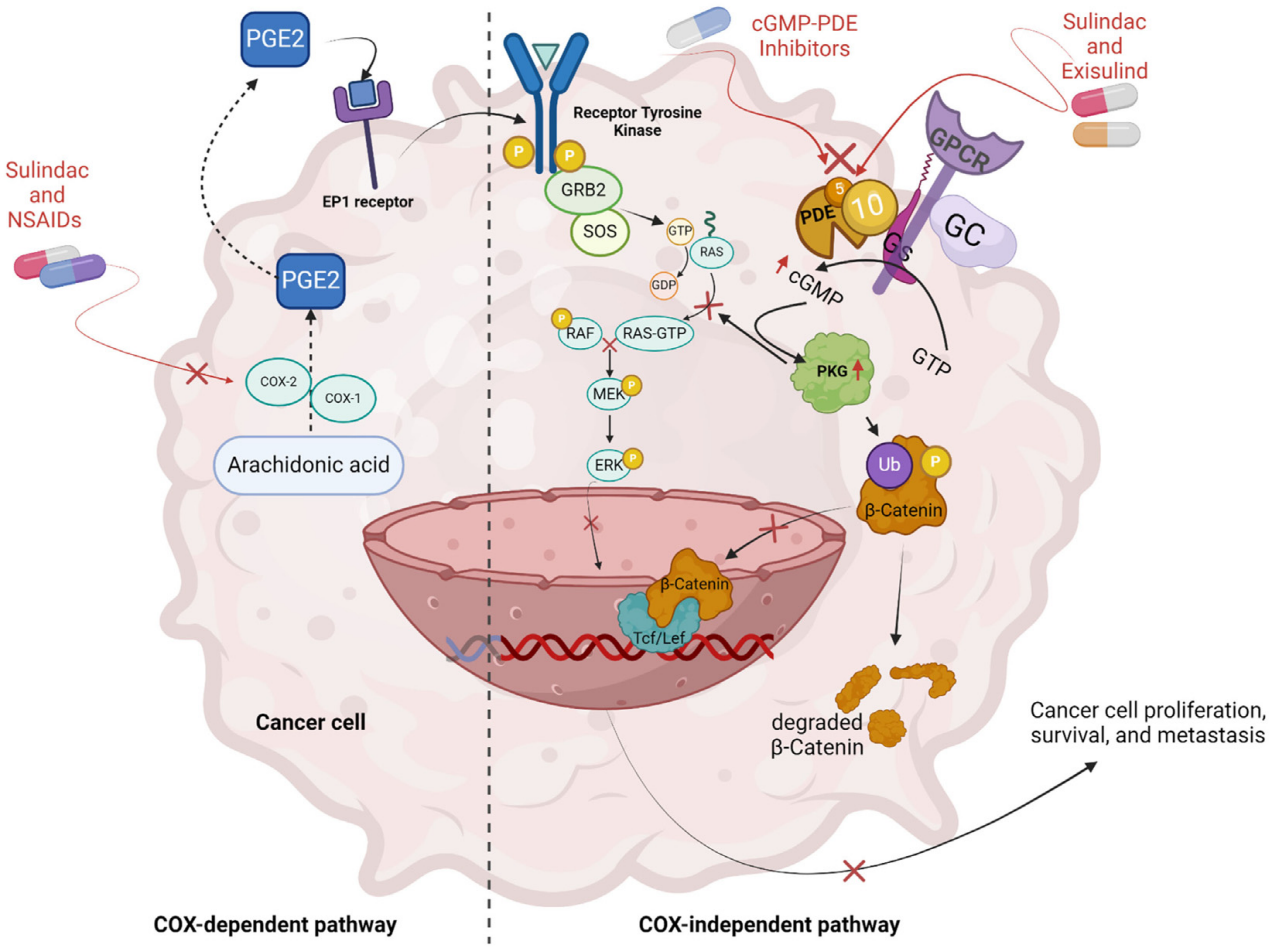
PDE5 and PDE10 regulation of cancer cell proliferation and survival. PDE5 and/or PDE10 regulate cGMP/PKG signaling to allow for *β*-catenin-dependent Tcf/Lef transcription and RAS/MAPK signaling resulting in the synthesis proteins essential for cancer cell proliferation and survival. Solid circles represent PDE5 and PDE10 that may be co-expressed whereby higher levels of PDE10 may compensate for the effects of a PDE5 isozyme specific inhibitor. Both COX-dependent and independent mechanisms are involved in anti-cancer activity of COX inhibitors. Red crosses symbolizes the impact of cGMP PDE and COX inhibitors. “Created with BioRender.com.”

Highly potent and selective inhibitors of PDE5 (eg, sildenafil) and PDE10 (eg, Pf2545920) have been reported to induce cell cycle arrest and apoptosis of cancer cell lines grown in vitro at concentrations that activate cGMP/PKG signaling (Li et al, 2015b; Mei et al, 2015; Lee et al, 2016). As depicted in Fig. 16, PKG activation results in the suppression of both *β*-catenin transcriptional activity and RAS/MAPK signaling. The mechanism for suppressing *β*-catenin transcriptional activity appears to result from PKG-mediated phosphorylation of *β*-catenin on residues known to induce ubiquitination and proteasomal degradation, resulting in reduced nuclear levels needed to activate Tcf/Lef transcription (Lee et al, 2016; Lee et al, 2021). The disruption of MAPK signaling by PKG activation may be attributed to disrupting RAS membrane localization and activation (Cho et al, 2016) or by interfering with receptor tyrosine kinase activity (Tao et al, 2012). The ability of activated PKG to block oncogenic *β*-catenin and RAS signaling simultaneously is consistent with reports that PDE10 inhibitors can suppress both signaling pathways in lung and ovarian cancer cell lines (Zhu et al, 2017; Borneman et al, 2022). This possibility is supported by the broad anticancer activity of PDE10 inhibitors and is significant, given that mutations in APC/*β*-catenin and RAS/MAPK pathway components drive most human cancers.

#### 3 Preclinical and clinical studies: cGMP PDE inhibition and anticancer activity

Based on the effect of PDE subtypes on tumor biology, (pre)clinical research has been conducted, which was hitherto limited to cGMP PDEs. We will now discuss the history and the gathered evidence for the application of cGMP PDEs in cancer treatment and pinpoint cross-talk with other pivotal signaling pathways.

##### a Early evidence for cGMP-PDE involvement: studies of exisulind and NSAIDs

The association between cGMP-degrading PDEs and the inhibition of cancer cell proliferation and survival was first reported from studies of sulindac sulfone, a metabolite of the nonsteroidal anti-inflammatory drug (NSAID), sulindac (Thompson et al, 2000). Sulindac sulfone (exisulind) was in clinical trials for the treatment and prevention of precancerous colon adenomas, but its mechanism of action was unknown. Exisulind and several analogs were found to inhibit the proliferation and induce apoptosis of colorectal cancer (CRC) and bladder cancer cell lines at concentrations that caused a sustained elevation of cGMP and activation of PKG that was not found with PDE5-selective inhibitors or other PDE inhibitors (Thompson et al, 2000; Piazza et al, 2001). Nonetheless, PDE5 was initially suspected as a likely target because it was the predominant cGMP-PDE degrading isoenzyme expressed in CRC and bladder cancer cell lines, and exisulind did not affect cAMP levels. Despite modest potency and no apparent PDE isoenzyme selectivity, exisulind significantly suppressed tumor formation in multiple, chemical-induced rodent models of colon, bladder, breast, lung, and prostate tumorigenesis, suggesting that the well established cancer chemopreventive activity of NSAIDs may involve a COX-independent mechanism of action (Piazza et al, 1997; Thompson et al, 1997; Malkinson et al, 1998; Reddy et al, 1999; Piazza et al, 2001; Narayanan et al, 2007). Exisulind showed promising efficacy in clinical trials of patients with familial adenomatous polyposis (FAP) or sporadic adenomas and selectively induced apoptosis of colonocytes in precancerous lesions without affecting colonocytes in the adjacent normal mucosa (Stoner et al, 1999; Arber et al, 2006). However, exisulind was not approved by the FDA because of liver toxicity that was likely attributed to low potency and lack of PDE isoenzyme selectivity.

The NSAID sulindac (*Clinoril*) also inhibits adenoma formation in FAP patients (Giardiello et al, 1993) and has broad cancer chemopreventive activity in experimental models of tumorigenesis. However, the long-term use of sulindac and other NSAIDs is not FDA-approved for long-term use because of potentially fatal toxicities resulting from COX-1 and COX-2 inhibition. Although the antineoplastic activity of sulindac and other NSAIDs is commonly attributed to COX-2 inhibition and suppression of prostaglandin synthesis (Wang and Dubois, 2010), numerous investigators have concluded that both COX-dependent and COX-independent mechanisms are involved (Fig. 16; Gurpinar et al, 2014).

Initial evidence suggesting that the anticancer activity of NSAIDs is mediated by an off-target mechanism involving cGMP PDE inhibition is based on experiments showing that the rank-order potency of a chemically diverse group of NSAIDs, including the COX-2 selective inhibitor, celecoxib, to inhibit in vitro growth of HT-29 CRC cells correlated with the inhibition of cGMP PDE but not of COX-2 (Tinsley et al, 2010). In addition, concentrations of NSAIDs required to inhibit cell growth far exceeded those required to block COX-1 or COX-2. These data, along with results of other experiments showing that cancer cell lines, which do not express COX-2, are sensitive to NSAIDs, and the inability of prostaglandins to rescue cell growth inhibition by NSAIDs, support a COX-independent mechanism for the cancer chemopreventive activity of sulindac and possibly other NSAIDs and COX-2 inhibitors. However, the contribution of COX-2-derived prostaglandins should not be excluded, given their broad biological activity that can have an impact on multiple oncogenic pathways (Wang and DuBois, 2006; Gurpinar et al, 2014).

##### b Preclinical research on PDE5 and PDE10 inhibition as possible anticancer therapies

Publications reporting that inhibitors of cGMP/PKG signaling, including NO donors, guanylyl cyclase activators, cell-permeable cGMP analogs, and certain cGMP PDE inhibitors, inhibit cancer cell proliferation and induce apoptosis suggest that PDE5 is essential for cancer cell proliferation and survival (Tinsley et al, 2009; Tinsley et al, 2010). Multiple investigations have supported this possibility by reporting that PDE5 is overexpressed in colon, bladder, breast, and lung cancers compared with noninvolved adjacent tissue (Chan et al, 2002; Piazza et al, 2001; Whitehead et al, 2003; Pusztai et al, 2003; Tinsley et al, 2009; Tinsley et al, 2010; Mei et al, 2015; Bisegna et al, 2020; Iwasaki et al, 2021; Tinsley et al, 2023). Consistent with the role of PDE5 in regulating cancer cell growth, PDE5 knockdown by siRNA selectively inhibited the growth of colon and breast cancer cell lines expressing high PDE5 levels compared with normal colonocytes or mammary epithelial cells with low PDE5 expression (Tinsley et al, 2009; Tinsley et al, 2011). In addition, gene silencing of *PDE5* in the highly aggressive human breast cancer cell line, MDA-MB-231, resulted in decreased cell motility and formation of lung metastases (Marino et al, 2014). Conversely, PDE5 overexpression in MCF-7 breast cancer cells led to increased motility and invasion (Catalano et al, 2016). Sildenafil and vardenafil were also reported to inhibit proliferation and induce caspase-dependent apoptosis in B-cell chronic lymphatic leukemia, which suggested the role of cGMP in regulating hematological malignancies (Sarfati et al, 2003). Finally, a sulindac derivative, sulindac benzylamide, with PDE5 selectivity that did not inhibit COX-1 or COX-2, potently inhibited CRC cell growth (Whitt et al, 2012).

The finding that sulindac sulfide, a metabolite of the NSAID, sulindac, attenuated CRC cell growth at concentrations that blocked PDE5 and PDE10 activity, inspired a drug discovery campaign to identify cGMP PDE inhibitors based on the same chemotype that did not target COX-1 and COX-2. This resulted in the synthesis of a large library of compounds sharing the indene scaffold of sulindac. Screening this library against recombinant COX, to confirm the lack of effect on prostaglandin synthesis, and PDE5 and PDE10, led to the discovery of several lead compounds, which displayed appreciably greater potency as inhibitors of cancer cell growth at concentrations that blocked PDE5 and/or PDE10. Chemical optimization of these indene-based inhibitors to improve their drug-like properties holds promise for further development of this novel anticancer therapy (Piazza et al, 2009; Li et al, 2013; Li et al, 2015a; Whitt et al, 2012; Lee et al, 2021; Borneman et al, 2022; Tinsley et al, 2023).

Given that known PDE5 inhibitors lacked sufficient binding affinity to arrest the proliferation of cancer cell lines in vitro, attention turned toward PDE10 because selective inhibitors (eg, PF2545920) were found to attenuate cancer cell growth with low micromolar potency. PDE10 is overexpressed in colon and lung adenocarcinomas relative to noninvolved adjacent tissue (Li et al, 2015b; Zhu et al, 2017) and interventions that reduced PDE10 activity (selective inhibitors, siRNA-mediated knockdown) arrested the proliferative response. Conversely, transfection of plasmid DNA encoding PDE10 to normal or precancerous colonocytes stimulated proliferation (Li et al, 2015b). Conversely, PDE10 inhibitors had a negligible impact on the proliferation of cells (colonocytes, airway epithelia) in which PDE10 levels were low or undetectable.

Further experiments showed that PDE10 is essential for CRC cell proliferation and survival by suppressing *β*-catenin transcriptional activity (Li et al, 2015a). The concentration range of a potent and selective PDE10 inhibitor (PF2545920) needed to inhibit CRC cell growth matched the concentration required for activation of cGMP/PKG signaling and suppression of *β*-catenin transcriptional activity and Wnt-induced translocation of *β*-catenin to the nucleus. Interestingly, PDE10 was found to colocalize with the oncogenic form of *β*-catenin in dysplastic colon regions from APC^min^ mice [ie, mice containing a multiple intestinal neoplasia (min) allele of the adenomatous polyposis coli (APC) loci, which encodes a nonsense mutation at codon 850 (Lee et al, 2021)]. These observations were significant because CRC is mostly driven by mutations in the APC/*β*-catenin axis. Oral administration of PF2545920 did not have antitumor activity in subcutaneous CRC mouse tumor models, but peritumoral injection to bypass liver metabolism suppressed growth in subcutaneous mouse tumor models (unpublished). Following these observations, a novel orally bioavailable PDE10 inhibitor derived from sulindac (ADT-061) was reported to potently and selectively inhibit CRC cell growth in vitro and suppress colon tumorigenesis in the APC^min^ mouse model (Lee et al, 2021).

Two other research groups independently found a link between poor prognosis in patients with non-small cell lung cancer (NSCLC) and PDE10A expression. In one of those studies, an activating mutation in the *PDE10A* gene as a driver of NSCLC was identified by using a combination of structural biology and genomic data (Shen et al, 2017). Fusco and colleagues observed a negative correlation between *PDE10A* mRNA and protein levels and overall and recurrence-free survival in NSCLC patients by comparing genomic data from high-risk and low-risk individuals (Fusco et al, 2018). The role of PDE10 in lung cancer was supported by studies showing that *PDE10* mRNA and protein were overexpressed in lung cancer cell lines compared with normal airway epithelial cells and lung tumors relative to normal lung tissue. Furthermore, PDE10 inhibitors and gene knockdown of *PDE10* selectively inhibited lung cancer cell growth by blocking both *β*-catenin transcriptional activity and MAPK signaling (Zhu et al, 2017). Another study extended those observations by reporting that PDE10 inhibitors, including ADT-061 (*aka* MCI-030), inhibited ovarian cancer cell growth by suppressing both *β*-catenin transcriptional activity and MAPK signaling (Borneman et al, 2022).

##### c Translational and clinical studies with PDE5 inhibitors

The role of PDE5 during the early stages of colon tumorigenesis was shown by Browning and colleagues who reported that sildenafil could suppress adenoma formation in mouse models of inflammation-induced CRC (Islam et al, 2017; Sharman et al, 2018). Similar cancer chemopreventive activity was reported with the guanylyl cyclase C agonist, Plecanatide (Chang et al, 2017). These findings supported the testing of PDE5 inhibitors and guanylyl cyclase C activators for CRC cancer chemoprevention in clinical trials, which was feasible, given that such drugs are FDA-approved for chronic indications (eg, erectile dysfunction and constipation) and generally well tolerated.

Case studies have reported that the long-term use of PDE5 inhibitors can reduce the risk of death or developing metastasis in male patients diagnosed with CRC, as well as lowering the risk of developing CRC in men with benign colon neoplasia (Huang et al, 2019a, 2020a). Although PDE5 inhibitors are FDA-approved for erectile dysfunction and pulmonary hypertension, their use for cancer prevention remains experimental and could have undesirable side effects (eg, thrombocytopenia) from long-term use. Recent studies by Browning and colleagues, describing a novel PDE5 inhibitor (malonyl-sildenafil) that inhibits the proliferation of colon epithelium in mice following oral administration without systemic absorption, hold promise for CRC chemoprevention (Lee et al, 2023). In addition, existing drugs that activate guanylyl cyclase C and are approved for constipation are being studied for CRC chemoprevention, which also acts locally in the colon (Rappaport and Waldman, 2020). The potential benefits of combining exisulind or sildenafil with chemotherapeutic drugs have also been studied in experimental models and in clinical trials as summarized in Table 2 but have not resulted in significant benefits for patients with advanced-stage malignancies.

**Table 2:** The clinical potential of combining exisulind or sildenafil with chemotherapeutic drugs

Inhibitor	Combined With; Outcome	Cancer Type	References
Exisulind	Cisplatin and paclitaxel; synergistic inhibition of cancer cell growth in culture	Lung cancer	Soriano et al 1999
Exisulind	Docetaxel; induced apoptosis, reduced tumor growth and metastasis and improved survival in mouse orthotopic model	Lung cancer	Soriano et al, 1999; Bunn et al, 2002; Whitehead et al, 2003
Exisulind	Capecitabine; combination well tolerated in breast cancer patients, but synergism appeared to be modest	Breast cancer	Pusztai et al, 2003
Sildenafil	Pemetrexed; suppressed tumor growth in mice that was enhanced with the mTOR inhibitor temsirolimus	Lung cancer	Booth et al, 2017
Sildenafil	Pemetrexed and sorafenib; enhanced activity in multiple cancer cell lines and mouse xenograft models	Lung cancer	Booth et al, 2017
Sildenafil	Doxorubicin; enhanced apoptosis and antitumor efficacy in mouse xenograft models, while attenuating doxorubicin cardiotoxic effects	Prostate cancer	Das et al, 2010
Sildenafil	OSU-03012 (non-COX inhibitor of celecoxib) and sorafenib; synergism to kill cancer cells in vitro and in vivo	Glioblastoma	Booth et al, 2015
Sildenafil	Doxorubicin; enhanced apoptosis and ROS production in rhabdomyosarcoma cell lines	Rhabdomyosarcoma	Urla et al, 2023

COX, cyclooxygenase; mTOR, mammalian target of rapamycin; ROS, reactive oxygen species.

##### d Dual PDE5 and PDE10 inhibition

Research has shown additive, antiproliferative activity following simultaneous blockade of PDE5 and PDE10 with small molecule inhibitors (MY5445 and papaverine, respectively) or hybrid PDE5/PDE10 inhibitors (eg, ADT-094). Similar data were obtained by silencing, simultaneously, PDE5 and PDE10, suggesting that the expression of these 2 enzyme families in neoplastic cells may cooperate to maintain low intracellular cGMP levels and, thus, provide proliferative and survival advantages to neoplastic cells during tumorigenesis (Li et al, 2015a).

Although the concentration range required for PDE5 and PDE10 inhibitors to suppress cancer cell growth is similar to those needed to inhibit cGMP hydrolysis in cell lysates and activate cGMP/PKG signaling in intact cells (Zhu et al, 2017), appreciably lower concentrations are needed to inhibit the isolated enzymes. This suggests that the effect of such inhibitors in cells is not isoenzyme selective and that the high concentrations nonselectively inhibit both isoenzymes. The co-expression of PDE5 and PDE10 in cancer cells may explain the discrepancy between cellular and biochemical experiments involving recombinant enzymes whereby the expression of 1 isoenzyme in cancer cells may compensate for the effects of an inhibitor that is specific for the other isoenzyme, resulting in the need for higher concentrations to inhibit both PDE5 and PDE10. In support of this hypothesis, experiments using dual PDE5/10 inhibitors or dual genetic knockdown of PDE5 and PDE10 resulted in greater growth suppression than inhibition of either isozyme alone (Li et al, 2015b). Furthermore, in the case of PDE5 inhibitors, sildenafil is a known substrate for ATP-binding efflux transporters, which may also account for the apparent discrepancy between potencies involving experiments in cells versus isolated enzymes (Ding et al, 2011).

##### e Neoplastic effects of PDE5 and PDE10 inhibition: conclusions

Mutations in *β*-catenin and RAS and their pathway components (eg, APC, RAF) account for most human cancers. These oncoproteins have been extensively studied but considered challenging or “undruggable” cancer targets, given that the cellular pathways they support are essential for the proliferation and survival of both cancer and normal cells. For example, Wnt-driven activation of *β*-catenin-dependent transcription is well known to be crucial for maintaining the survival of normal stem cells, and growth factor activation of RAS-driven/ MAPK/AKT signaling is needed for normal cell turnover or in response to injury. The observation that PDE10 is overexpressed in certain cancers and essential for cancer cell growth and that PDE10 inhibitors can suppress both *β*-catenin and RAS signaling suggests an unrecognized strategy to kill cancer cells selectively. Although conventional PDE10 inhibitors were developed for CNS conditions and may not be able to achieve adequate systemic levels for anticancer activity in experimental mouse tumor models, novel PDE10 inhibitors that can achieve systemic levels needed to kill cancer cells selectively warrant further investigation for treating cancers harboring mutations in *β*-catenin or RAS or pathway components. There is potential for safety, given that PDE10 has low expression in most peripheral tissues with no known physiological function. Finally, evidence that PDE5 and PDE10 inhibitors can activate mechanisms of antitumor immunity suggests potential benefits in combination with immunotherapy (see Fig. 16).

### G The role of PDEs in immune regulation

#### 1 General background

In previous chapters, the important contribution of inflammation in the role of PDE function in pathophysiology is accented ubiquitously. The immune response and its mediators are involved in the pathogenesis of various diseases, and we here highlighted tissue remodeling effects in internal organs, fibrosis and extracellular matrix formation, and neurodegeneration. Alterations in the immune environment of tissues have categorically become a part of the standard variables to be measured in studies on the pathobiology of diseases. With respect to PDEs, comprehensive knowledge has especially been built up in the framework of tumor progression, which is discussed in depth. In this chapter, the involvement of cyclic nucleotide signaling in the regulation of cells of the innate and adaptive immune system is summarized. This will start with a comprehensive review of the role of tumor microenvironment (TME). Thereafter, the relevance of each individual immune cell effect is illustrated with typical examples in cancer and internal organ disease to facilitate the extrapolation to the expertise of the readership. Due to its comprehensiveness, this latter part uses a 2-layer mode to transfer the knowledge, the first layer being the cell type, and as a second layer the role of the PDE subtype.

#### 2 PDEs in the tumor immune microenvironment

##### a General aspects

It has become increasingly clear that the effectiveness of many anticancer drugs relies, in part, on the host immune system. Mounting evidence indicates that the immune composition of the TME can profoundly influence tumor response to treatments. For example, immunologically “hot” tumors, which show signs of inflammation and are infiltrated with T lymphocytes, tend to respond well to immune checkpoint inhibition (ICI) therapy. In contrast, “cold” tumors, characterized by a lack of T cell infiltration, are resistant or refractory to immunotherapy (Galon and Bruni, 2019). In terms of T cells, their functional status is an important determinant of the host antitumor immunity. It has been well established that chronic antigenic stimulations, which often occur during chronic viral infections and cancer development, can lead to functional exhaustion in CD8^+^ T cells, characterized by gradual loss of the ability to proliferate, persist, and produce inflammatory cytokines (Wherry and Kurachi, 2015; Schietinger et al, 2016). Exhausted CD8^+^ T cells are phenotypically and functionally heterogeneous, consisting of progenitor, transitory, and terminally exhausted cells, each characterized by distinct transcriptional and epigenetic signatures as well as varied responsiveness to anti-PD-1 ICI therapy (Sade-Feldman et al, 2018; Siddiqui et al, 2019; Miller et al, 2019; Beltra et al, 2020). The TME is often enriched in regulatory T cells (T_reg_), which are a subset of CD4^+^ T cells known to suppress antitumor immunity. Besides T lymphocytes, myeloid cells in the TME are also known to impact tumor progression and response to therapies. Extensive studies have demonstrated that a subset of aberrantly developed immature myeloid cells, termed myeloid-derived suppressor cells (MDSCs), promote tumor growth, metastasis, and immune evasion, presenting a major hindrance to the effectiveness of various types of cancer treatments.

It has been shown that *β*-catenin signaling in tumor cells can prevent dendritic cell (DC) recruitment, resulting in T cell exclusion in the TME and tumor resistance to checkpoint immunotherapy (Spranger et al, 2015; Spranger et al, 2017; Luke et al, 2019). Not only does tumor-intrinsic *β*-catenin signaling promote tumor immune evasion (Spranger and Gajewski, 2015) but *β*-catenin activation in DCs also contributes to immune tolerance (Suryawanshi and Manicassamy, 2015). Tumors can induce the activation of *β*-catenin in DCs in the draining lymph nodes, rendering them tolerogenic, which induces T_reg_ cells to suppress antitumor activity.

##### b The role of PDE in inflammation and TEM

The fact that PDE11A loss of function is associated with an increased risk of various tumors may be related to its likely role in regulating inflammation. Interestingly, PDE11A expression can be induced by stress and immune activation signals in cells that do not basally express the enzyme (Witwicka et al, 2007; Bazhin et al, 2010; Zhu et al, 2019b). Furthermore, reduced PDE11A4 expression in the brain correlates with increased expression of the proinflammatory cytokine, IL-6, increased cytokine release, and increased microglial activation (Pathak et al, 2016; Pilarzyk et al, 2021). Conversely, increased PDE activity may also be relevant. As PDE inhibition emerges as an attractive therapeutic strategy for cancer treatment, there is growing interest in understanding whether and how PDE5 and PDE10 inhibitors impact the various immune components in the TME. Mechanistic explanations of the role of PDE5 on tumor biology can be found in their effects on various immune cells. With respect to MDSC, it was first reported that PDE5 inhibition by sildenafil or tadalafil led to enhanced intratumoral T cell infiltration and activation, along with improved tumor growth control in multiple mouse transplant tumor models (Serafini et al, 2006). These beneficial effects were lost in immune-deficient mice, indicating that the antitumor effect of PDE5 inhibitors was immune-mediated. Mechanistically, the restoration of antitumor immunity in tumor-bearing mice was due to abrogation of MDSC-mediated immune suppression as PDE5 inhibitors downregulated arginase 1 and NO synthase–2, the main mediators of MDSC immunosuppressive activity. A subsequent study using a spontaneous mouse melanoma model confirmed that PDE5 inhibition by sildenafil resulted in reduced MDSC accumulation and immunosuppressive function, accompanied by restoration of CD8^+^ T cell effector activity and improvement in mouse survival (Meyer et al, 2011).

In further explanation of the role of PDE5, it is conceivable that suppressing *β*-catenin signaling, either in tumor cells or DCs, with PDE5 inhibitors can overcome some of the major immunosuppressive mechanisms (MDSCs, tolerogenic DCs, and T_reg_ cells) in the TME, thereby improving tumor response to immunotherapy. The observed beneficial effects of PDE5 inhibitors in mouse tumor models and clinical studies, including reduced T_reg_ presence and increased tumor infiltration of activated CD8^+^ T cells, may be driven by fully activated DCs as the result of *β*-catenin suppression secondary to PDE5 inhibition. The mechanisms linking PDE inhibitor-induced *β*-catenin suppression to DC and T cell recruitment/activation in the TME require elucidation.

Evidence for the role of immune modulation by PDE5 in tumor progression has also been found in humans. Clinical trials were conducted to evaluate whether PDE5 inhibition can revert tumor-induced immunosuppression and promote antitumor immunity in patients with head and neck squamous cell carcinoma, metastatic melanoma, or multiple myeloma (Califano et al, 2015; Weed et al, 2015; Hassel et al, 2017). These trials indicate that tadalafil administration correlated with reduced MDSC accumulation and/or suppressive function and improved T cell activation, with 1 trial reporting additional reduction of T_reg_ cells. However, future research should examine whether PDE5 inhibition also affects MDSC induction or recruitment because a reduction in MDSC accumulation was observed in some but not all published studies. Moreover, the exact function and expression kinetics of PDE5 in MDSCs and T_reg_ cells warrant further investigation.

The immunological impact of PDE10 inhibition has remained largely unexplored. It is reasonable to speculate that PDE10 inhibitors mirror PDE5 inhibitors in exerting immunomodulatory effects because their mechanisms of action overlap in terms of activation of cGMP/PKG signaling and suppression of *β*-catenin transcriptional activity. It is worth noting, that some of the newly developed PDE10 inhibitors suppress oncogenic RAS signaling (Zhu et al, 2017b; Borneman et al, 2022), raising the possibility that they may improve tumor immunogenicity. Aberrant RAS activation occurs in about 20% of all malignancies, with high incidences found in pancreatic cancer (90%), colorectal cancer (50%), and lung cancer (30%; Bos, 1989). Oncogenic RAS signaling is known to promote immune suppression (Weijzen et al, 1999). It has been reported that KRAS mutations induce T_reg_ cells (Zdanov et al, 2016; Cheng et al, 2019), upregulate PD-L1 in cancer cells (Sumimoto et al, 2016; Chen et al, 2017; Coelho et al, 2017), but downregulate MHC class I molecules (Atkins et al, 2004; El-Jawhari et al, 2014). In addition, RAS activation promotes tumor production of G-CSF and GM-CSF, which induce and expand MDSCs to facilitate tumor progression, metastasis, and immune suppression (Pylayeva-Gupta et al, 2012; Phan et al, 2013). With the advances in developing mutant-specific inhibitors targeting KRAS G12C (Ostrem et al, 2013), RAS is no longer considered an undruggable target (Molina-Arcas et al, 2021). It is reassuring that in preclinical studies, many of these newly developed KRAS inhibitors significantly improved antitumor immunity by reducing MDSCs, enhancing antigen presentation and CD8^+^ T cell priming. These data validate RAS inhibition as a potent immune-potentiating strategy in addition to its direct tumoricidal effect (Canon et al, 2019; Briere et al, 2021; Mugarza et al, 2022; Zhang et al, 2022; Kemp et al, 2023). Likewise, the emergence of novel PDE10 inhibitors that are capable of suppressing oncogenic RAS and *β*-catenin activities have the potential to reverse an immunosuppressive TME, a feature that awaits to be exploited to drive durable therapeutic outcomes.

#### 3 The role of PDEs in cells of the innate and adaptive immune systems

Cyclic nucleotide signaling in the regulation of immune response has been on the map for a long time. Previous publications described that cAMP plays a critical role as a second messenger (Rall and Sutherland, 1958; Sutherland and Rall, 1958) and has been shown to be a key regulator of the activation and function of cells of the innate (Schafer et al, 2014; Schafer et al, 2019) and adaptive immune system (Bourne et al, 1974; Wang et al, 1978; Amarandi et al, 2016). Numerous anti-inflammatory drugs successfully target molecules of the cAMP signaling pathway including several FDA-approved medications (Rabe, 2011; Schett et al, 2010; Tenor et al, 2011; De Souza et al, 2012; Milara et al, 2012; Wittmann and Helliwell, 2013; Victoni et al, 2014; Schafer et al, 2014; Dong et al, 2016; Jarnagin et al, 2016; Sriram and Insel, 2018; Blokland et al, 2019; Baillie et al, 2019b; Schafer et al, 2019). As described in section General Introduction, cAMP is degraded by cyclic nucleotide PDEs (Lerner and Epstein, 2006; Conti and Beavo, 2007), which constitute a group of enzymes known to hydrolyze cAMP and cGMP and, hence, maintain spatial and temporal control over its activity. Cells of the innate and adaptive immune system play a critical role in inflammation and its modulation (Medzhitov, 2021; Meizlish et al, 2021). Function and regulation of different subpopulations of T cells, B cells, and natural killer (NK) cells, as well as myeloid cells, such as neutrophils, monocytes, macrophages, and DCs involve activation of the cAMP pathway. Additionally, interactions between leukocytes and endothelial cells are critical during the formation of inflammatory lesions and can be regulated by cAMP/cGMP signaling. As a general rule, cAMP levels in cells of both the innate and adaptive immune systems are correlated with their inflammatory activities (Bourne et al, 1974; Wang et al, 1978; Mosenden and Tasken, 2011; Vang et al, 2013; Schafer et al, 2014; Rueda et al, 2016; Schafer et al, 2019). Although PDEs have been recognized as potential drug targets for anti-inflammatory drugs, developing specific PDE inhibitors has faced challenges, primarily due to side effects; nevertheless, progress has been made with the approval and clinical use of selected PDE4 inhibitors for treating major inflammatory diseases (*vide supra*). This chapter will discuss the role of PDEs in cells of the innate and adaptive immune system (Table 3).

**Table 3:** Summary of PDE isoform expression and function in immune cells

Immune Cell Subpopulation	PDE Gene Expression	PDE Protein Expression /Activity	PDE Activity and Methods	Results
Dendritic cells		PDE1, PDE3 membrane bound, PDE4, PDE7	PDE isoenzyme activity	Pro-inflammatory cytokine production
	*PDE4b*	PDE4B	PDE isoenzyme activity	DC mediated Th2-dependent immunopathology
			PDE4 inhibitor studies	
			PDE4B gene knockout studies	Selective suppression of Th2 polarization of T cell subpopulations in vivo
Granulocytes			PDE4 inhibitor studies	In vitro suppression of neutrophil functions
				suppression of chemotaxis, inflammation, neutrophil and eosinophil infiltration in vivo
	*PDE4A, D*	PDE4A, D	PDE4A and D expression studies	Basophils
NK cells		PDE3 and 4	PDE3 and 4 inhibitor studies	
			PDE4 inhibitor studies	Suppression of TNF-*α* production Suppression of IFN-*γ* production
		IBMX sensitive PDEs	Broad PDE inhibitor studies	
Monocytes and macrophages		PDE1	PDE isoenzyme activity, inhibitor studies	
		PDE3		Suppression of TNF-release through PDE4 ±PDE3 inhibition
		PDE4		
Macrophages	*Pde4 deficient mice*	Pde4 deficient mice global		Suppression of cytokine production Protection from LPS-induced shock
	*PDE8A*	PDE8A		Promotion of susceptibility to HIV-1 infection
		PDE10A	Inhibition of PDE10, PDE10A deficient mice	Altered cytokine and chemokine production
Microglia		PDE1	Inhibition of PDE1 activity	Suppression of cytokine expression, Inhibition of cytokine release, suppression of cytokine gene expression, suppression of motility
CD4+ T cells	*PDE1*	PDE1	PDE1 activity	Upregulation under mitogen stimulation
	*PDE2*	PDE2	PDE2 expression and activity	Expressed in mouse T cells but not human T cells
		PDE2A	PDE2 facilitates T cell activation	Mouse T cell activation
	*PDE3*	PDE3, PDE3B	Membrane bound PDE3B activity,	Foxp3 repression of PDE3B in Treg cells
	*Pde3b*		Pde3b deficient mice	Reduced PDE3B expression permits normal Treg cell homoeostasis and Treg cell-specific gene expression
		PDE4A	PDE activity and inhibition	T cell activation and functions
	*Pde4b*	PDE4B	PDE activity and inhibition, PDE4B deficient mice	T cell activation and functions, Th subset polarization
		PDE4D	PDE activity and inhibition	T cell activation and functions
		PDE4	PDE activity, binding, overexpression and inhibition	Modulation of signal transduction through the T cell receptor Regulation of full T cell activation
	*PDE7*	PDE7	Expression and anti-sense inhibition studies	Upregulation of PDE8A1 after polyclonal T cell activation
	*PDE7A1, A3*	PDE7A1, A3		
Mitogen-activated splenocytes, anti-CD3 activated CD4+ T cells, antigen exposed naïve and memory CD4+ T cells	*PDE8A1*	PDE8A1	Induction of PDE8A expression in response to stimulus, PDE8 inhibition	Upregulation of PDE8A1 after polyclonal T cell activation
	*Pde8a*	PDE8A	Induction of PDE8A expression in response to stimulus, PDE8 inhibition via enzymatic inhibitor and peptide disruptor, in vivo suppression of EAE	Induction of PDE8A expression in response to stimulus, Association of PDE8A expression and accumulation of sensitized T cells in draining lymph node of in an animal model of allergic airway disease AAD
CD4+ effector Teff cells	*PDE8A*	PDE8A	PDE expression	PDE8A inhibition by enzymatic inhibitor or a PDE8A-Raf-1 kinase
CD4+ regulatory Treg cells	*Pde1a, Pde1b*	PDE1A, B		
	*Pde2a*	PDE2A		High cAMP levels in T cells
	*Pde 3b*	PDE3B		
	*Pde 4b*	PDE4B		
	*Pde5a*	PDE5A		
	*PDE8A*	PDE8A		
B cells	*PDE4A, B, D*	PDE4A, B, D	Expression	
	*PDE7B*	PDE7B	PDE7 inhibition	Induction of apoptosis through PDE7 inhibition

#### 4 Role of PDEs in specific immune cell types

*Dendritic Cells*. DCs are heterogeneous and are commonly classified into subtypes including plasmacytoid DC (pDC), myeloid or conventional DC1 (cDC1), and myeloid or conventional DC2 (cDC2), as well as tissue-specific DCs such as Langerhans cells (Collin and Bigley, 2018). Early studies indicated that during differentiation of DCs, PDE4 activity decreased, whereas activities of PDE1 and PDE3 increased. Of note, rolipram, at PDE4-selective concentrations, blocked LPS-induced TNF*α* release by ∼37%. In contrast, the PDE3 inhibitor, motapizone, only marginally influenced TNF*α* synthesis, but a synergistic inhibitory effect was noted in combination with rolipram (Gantner et al, 1999). In addition, the PDE4 inhibitor, roflumilast, has been shown to be a potent immunomodulator of DC cell function (Hatzelmann and Schudt, 2001). In mice, the cAMP-PKA-CREB signaling pathway orchestrates many functional aspects of cDC2s (Schafer et al, 2014; Lee et al, 2020). Notably, PDE4B is highly expressed in mouse DCs (Chinn et al, 2022). Using mice deficient in G*α*s, PDE4B was shown to be a key regulator of cellular cAMP concentrations in DCs and played a role in DC-mediated T helper cell type 2 (Th2) dependent immunopathology (Jin et al, 2010; Chinn et al, 2022). PDE4 inhibition in DCs has also been shown to reduce their ability to induce T helper cell type 1 (Th1) cells from naive T cells in vitro, an effect that was largely attributed to an effect on PDE4A (Heystek et al, 2003).

*Granulocytes*. The immunosuppressive effect of the PDE4 inhibitor, roflumilast, on a wide range of neutrophil functions in vivo and in vitro is well documented (Hatzelmann and Schudt, 2001; Wollin et al, 2005; Jones et al, 2005; Sanz et al, 2007; Cortijo Gimeno and Morcillo Sanchez, 2010; Hatzelmann et al, 2010; Nials et al, 2011; Koga et al, 2016; Tsai et al, 2023; Lin et al, 2023a; Chang et al, 2024). Several studies demonstrated that PDE4 inhibitors suppress oxidative stress and chemotaxis in neutrophils (Hatzelmann and Schudt, 2001; Jones et al, 2005; Wollin et al, 2005; Sanz et al, 2007; Cortijo Gimeno and Morcillo Sanchez, 2010; Hatzelmann et al, 2010; Nials et al, 2011; Koga et al, 2016; Tsai et al, 2023; Lin et al, 2023a; Chang et al, 2024). Additionally, PDE10A was shown to regulate neutrophil infiltration in a mouse model of lung inflammation (Hsu et al, 2021). PDE inhibitors (ciclamilast, piclamilast, IBMX) have also been shown to effectively suppress pulmonary eosinophilia in rodent models of allergic airway disease (Zheng et al, 2019; Lee et al, 2020).

##### a Monocytes and macrophages

*Monocyte differentiation*. Changes in cyclic nucleotide levels have been shown to have a profound impact on the phenotypical differentiation of monocytes (Schudt et al, 1995; Gantner et al, 1997a; Hertz and Beavo, 2011; Tenor et al, 2011). Importantly, during in vitro differentiation of human blood-derived monocytes, the PDE profile undergoes significant remodeling, which parallels that in human alveolar macrophages (Schudt et al, 1995; Tenor et al, 1995a). Major changes in PDE1, PDE3, and PDE4 activities are seen, whereas PDE4 activity, the major PDE isotype of peripheral blood monocytes, rapidly declines under in vitro culture (Gantner et al, 1997a). Subsequent investigations have delineated the role and function of various PDEs in monocytes and macrophages, and those findings are described in more detail below.

*PDE1*. Bender and colleagues reported the selective upregulation of PDE1B2 during monocyte-to-macrophage differentiation (Bender et al, 2005). Recently, an inhibitor of Ca^2+^/CaM-dependent PDE1 was shown to suppress LPS-induced expression of genes encoding proinflammatory cytokines and motility in rodent microglial cells (O'Brien et al, 2020; Zhou et al, 2023).

*PDE4*. The expression and function of specific PDE4 isoforms in monocytes and macrophages are differentiation-dependent (Shepherd et al, 2004; Schafer et al, 2014). In gene knockout studies in mice, Conti and colleagues demonstrated, in vivo and ex vivo, the selective regulation of the LPS-Toll-like receptor signaling pathway in macrophages by Pde4b, but not Pde4a or Pde4d (Jin et al, 2005).

*PDE10*. Recent reports indicate a role of PDE10A in lung inflammation mediated by macrophages. Treatment of murine macrophages with LPS in vitro induces a sustained expression of PDE10A, in contrast to a more transient induction of PDE4B. Similarly, LPS-induced cytokine and chemokine production were differentially affected by selective inhibition of PDE10 versus PDE4. These results were supported by in vivo experiments performed in Pde10a-deficient mice, or in mice treated with a PDE10 selective inhibitor (Hsu et al, 2021).

*Natural Killer Cells.* As with other cells of the immune system, raising the levels of cAMP suppresses the activity of NK cells (Shepherd et al, 2004). Studies have focused on the regulation of NK cells by a broad variety of PDEs and selective isoforms including PDE3 and PDE4 (Whalen and Crews, 2000; Walker and Rotondo, 2004; Schafer et al, 2010; Chen and Yan, 2021). The PDE4 inhibitor, apremilast, has been shown to suppress the proinflammatory activity of most cells of the innate and adaptive immune system, including TNF*α* production by NK cells in a model of psoriasis (Schafer et al, 2010). Notably, PGE_2_-mediated NK cell suppression in a tumor environment can be reversed through external exposure to IL-15, which acts, in part, by upregulating the expression of PDE4A (Chen and Yan, 2021). Similarly, the broad-spectrum PDE inhibitor, IBMX, suppresses interferon (IFN) *γ* synthesis by NK cells (Walker and Rotondo, 2004).

*T cells*. PDEs in human lymphocytes, particularly T cells, have proven to be very effective therapeutic targets for treating inflammation, with 3 PDE4-selective inhibitors now approved and in the clinic for the treatment of several diseases, including COPD, psoriasis, psoriatic arthritis, atopic dermatitis, and seborrheic dermatitis. There is evidence that PDEs other than PDE4 may also be effective therapeutic targets for treating certain inflammatory conditions, and in the following sections, we review what is known about PDEs that are expressed in T cells, and how they might be useful as targets for treating inflammation.

*PDE1*. The Ca^2+^/CaM-dependent PDE1 gene family all hydrolyze cGMP with *K*_m_s in the low micromolar range but differ in their affinities for cAMP [1 *μ*M, 7–24 *μ*M and 50–100 *μ*M for PDE1C, PDE1B, and PDE1A, respectively (Lerner and Epstein, 2006)]. Early analyses of quiescent human peripheral blood lymphocytes (HPBL) showed little or no expression of PDE1 (Epstein and Hachisu, 1984; Epstein et al, 1987; Tenor et al, 1995b; Giembycz, 1996). It was also shown in early studies that cAMP PDE activity was highly elevated in murine (Hait and Weiss, 1976) and human (Epstein et al, 1977) transformed lymphocytes associated with hematological malignancies, and PDE activity was greatly induced in HPBL following activation with mitogenic agents such as phytohemagglutinin (Epstein et al, 1980). Subsequently, PDE1 activity was shown to be expressed in a human B lymphoblastoid cell line (Epstein et al, 1987), and further analyses showed that PDE1B1 was present in both T and B lymphoblastoid cell lines and induced in HPBL following mitogenic activation (Jiang et al, 1996; Jiang et al, 1998; Kanda and Watanabe, 2001). The full open reading frame of the PDE1B1 cDNA was cloned from a human lymphoblastoid cell line and antisense oligodeoxynucleotides designed to inhibit the expression of PDE1B1-induced apoptosis in these cells (Jiang et al, 1996), but not in quiescent HPBL (Epstein, 1998).

The recent development of potent, selective inhibitors of PDE1 has now facilitated the testing of PDE1 as a therapeutic target for combating inflammation in a number of experimental systems. As noted in the section on monocytes and macrophages, the PDE1 inhibitors, lenrispodun (PDE1B IC_50_ = 58 pM; O'Brien et al, 2020), and compounds **4a** and **5f** (PDE1C IC_50_s = 2.5 nM and 4.5nM, respectively) discussed by Zhou and colleagues suppressed brain neuroinflammation by preventing microglia migration as well as blocking nuclear factor-*κ*B-mediated release of inflammatory mediators and activation of the cAMP/CREB axis (Zhou et al, 2023). Lenrispodun also reduced inflammatory cytokine levels and ameliorated vascular function and inflammatory responses in an Ercc1^Δ/−^ murine model of aging (Golshiri et al, 2021a). Naringenin, a polyphenolic flavonoid isolated from citrus fruit, was shown to bind to CaM and inhibit CaM-stimulated PDE1 activity; it also inhibited LPS-induced inflammatory cytokine release from the SK-OV-3 and K562 human cell lines, which were immortalized from patients with ovarian serous cystadenocarcinoma and chronic lymphocytic leukemia, respectively (Afshari et al, 2023). Early studies had shown that nimodipine, a 1,4-dihydropyridine calcium channel antagonist, was capable of directly inhibiting PDE1 at low micromolar concentrations (Epstein et al, 1982). Based on this observation, structural modifications of nimodipine were made that increased potency and selectivity for PDE1. One of those compounds, **2g,** synthesized by the Wu laboratory (PDE1C IC_50_ = 10 nM), exhibited anti-inflammatory properties by reducing the expression of TGF*β* and attenuating fibrosis in a bleomycin-induced lung fibrosis rat model (Huang et al, 2022). Another potent PDE1 inhibitor, a quinoline-2 (1H)-1 derivative, compound 10c (PDE1C IC_50_ = 15 nM), inhibited the LPS-induced release of inflammatory cytokines from the murine macrophage cell line RAW264.7 and exhibited appreciable clinical improvement in a dextran sodium sulfate-induced mouse model of inflammatory bowel disease (Zhang et al, 2023a,b). Hence, PDE1 may prove to be an effective target for treating inflammation.

*PDE2*: Human T cells express little or no PDE2 (Tenor et al, 1995a; Giembycz, 1996). In contrast, in murine thymocytes, PDE2 represents as much as 80% of the total hydrolytic activity when activated by cGMP (Michie et al, 1996). A recent study investigated the expression of PDE2A and PDE3B in murine conventional (T_con_) and T_reg_ cells at rest and after activation with anti-CD3/CD28 and cGMP-elevating natriuretic peptides on cAMP levels and on early activation markers, CD25 and CD69 (Kurelic et al, 2021). In that study, engagement of the T-cell receptor (TCR) led to the selective upregulation of PDE2A protein expression in T_con_ cells, whereas no change in expression was detected in T_reg_ cells. In contrast, TCR engagement significantly increased PDE3B levels in both resting and activated T_con_ subsets but not in T_reg_ cells. Thus, it seems that the elevation of cGMP led to an increase in cAMP levels in nonactivated T_con_ cells, presumably through the inhibition of PDE3B (which would be more predominant in the non-activated state), and this led to a decrease in cAMP levels in activated T_con_ cells, where PDE2A, the cGMP-activated PDE, is induced. When assessing if this cGMP/cAMP crosstalk following the activation of T_con_ cells might have functional effects, it was found that cGMP elevation by atrial natriuretic peptide enhanced T_con_ activation as indicated by enhanced expression of the early activation markers CD25 and CD69. Furthermore, this effect was blocked by a PDE2A selective inhibitor indicating that this was due to a cGMP-mediated reduction in cAMP levels secondarily to the activation of PDE2A. Hence, at least in mice, PDE2A may play a functional role in T cell activation.

*PDE3*. An early investigation of PDE activity in HPBL supernatants identified a single, prominent form of cAMP PDE based on DEAE anion-exchange chromatography, isoelectric focusing and glycerol gradient analysis, enzymes kinetics, and inhibitor sensitivity (Epstein and Hachisu, 1984). Subsequently, a study of whole homogenates of purified human T lymphocytes showed 2 separable high-affinity cAMP PDEs on HPLC columns, with 1 peak characteristic of PDE4 being purely cytosolic, and the other peak characteristic of PDE3 localized exclusively in the particulate fraction (Robicsek et al, 1989; Robicsek et al, 1991). Other studies with purified human T cells confirmed the presence of PDE3 in the particulate fractions and showed PDE3 activity to be represented exclusively by PDE3B with no evidence of PDE3A (Tenor et al, 1995a; Ekholm et al, 1997; Giembycz, 1996; Sheth et al, 1997). In contrast to T cells, purified human B cells express no mRNA for PDE3B and only very marginal expression of PDE3A mRNA (Gantner et al, 1998). The expression of PDE3B in T_reg_ cells is considerably reduced compared with that in T_con_ cells, and it appears that the low catalytic activity of PDE3B is critical for the regulation of T_reg_ cell-specific gene expression (Gavin et al, 2007). Functionally, PDE4 inhibitors suppressed PHA- and anti-CD3-induced proliferation of purified human CD4^+^ and CD8^+^ T-lymphocytes and the release of IL-2 and IFN*γ*, whereas inhibitors of PDE3 did not. However, although inactive by themselves, PDE3 inhibitors potentiated the inhibitory activity of PDE4 inhibitors on these processes (Giembycz, 1996). In a similar vein, inhibition of PDE3B augmented PDE4 inhibitor-induced apoptosis in a subset of patients with chronic lymphocytic leukemia who were resistant to PDE4 inhibition alone (Moon et al, 2002).

**Fig. 17:**
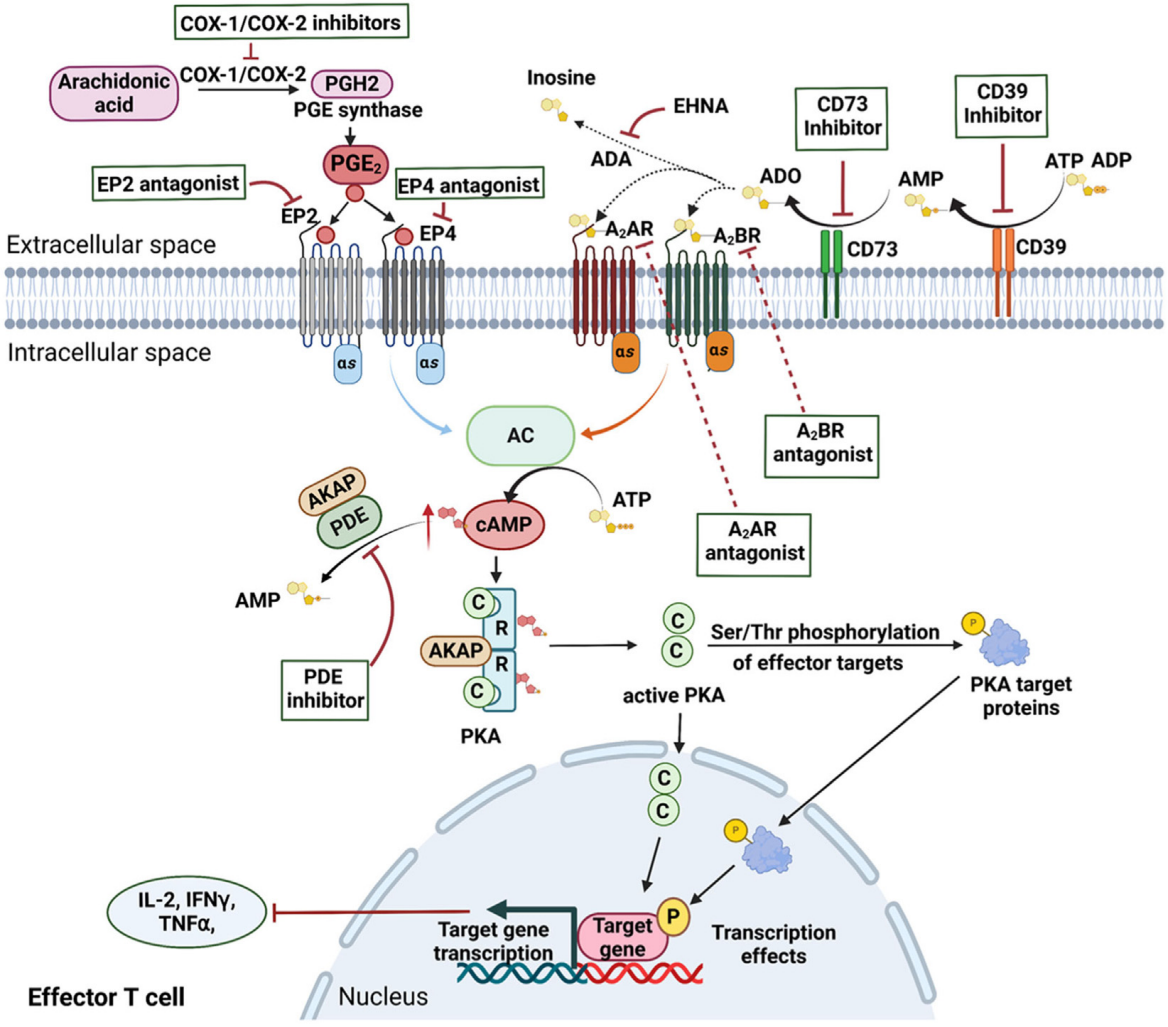
The cAMP-PKA-PDE pathway in T cells. An example of GPCR-mediated signaling through several receptors leading to activation of cAMP signaling in T cells. Here, ADO and PGE_2_ serve as ligands for their cognate GPCRs A2AR/A2BR and EP2/EP4, respectively, allowing the activation of AC, which then catalyzes the synthesis of cAMP from ATP. cAMP can bind to and activate the regulatory subunit of PKA, inducing the release of the active catalytic subunit, which enables dedicated pools of PKA anchored to several AKAPS to phosphorylate downstream proteins involved in immune regulation, including transcriptional programs. cAMP-specific PDEs, on the other hand, can hydrolyze cAMP to AMP in a feedback mechanism to attenuate the signal, thereby sculpting and compartmentalizing the cAMP pool. ADO and PGE_2_ receptor antagonism as well as PDE inhibition, are current strategies for drug development.

**Fig. 18:**
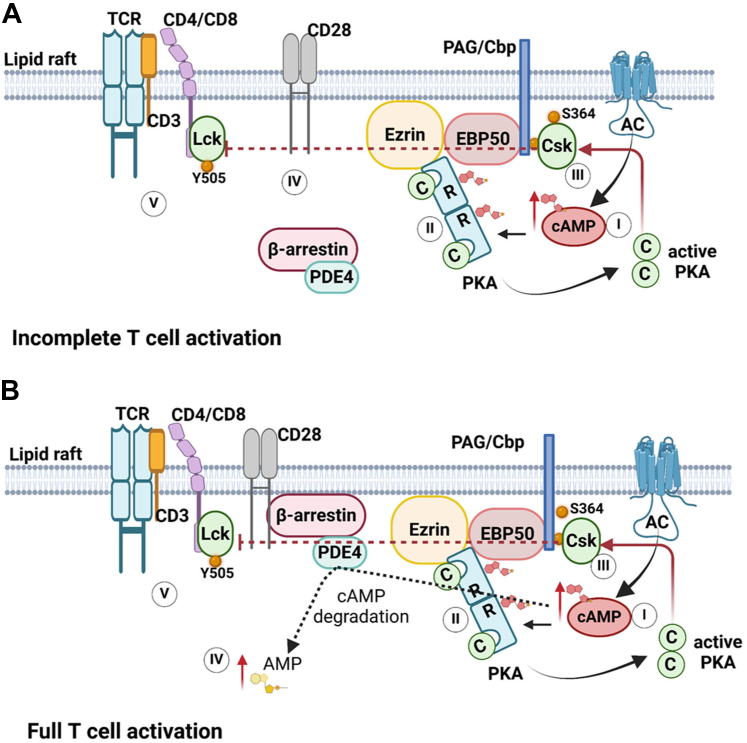
The opposing roles of PKA and PDE4 in proximal T cell signaling. Inhibition of T cell activation by cAMP is facilitated by a signaling complex in lipid rafts, consisting of PKA, Ezrin, EBP50, Cbp/PAG. This complex works as an immunoregulatory pathway, hindering TCR-induced T-cell activation. In T cells, TCR activation through G-protein coupling to AC generates cAMP (I), allowing PKA activation (II). The membrane-bound cytoskeleton linker protein Ezrin then acts as an AKAP and positions PKA to phosphorylate Csk (III) through the PKA-Ezrin-EBP50-Cbp/PAG-Csk complex, which in the absence of CD28 costimulation (IV), allows Csk to phosphorylate the Src family kinase Lck (V) at the C-terminal inhibitory site, hence, preventing full T cell activation (A). CD28 costimulation, however, increases TCR-induced signals by recruiting the PDE4/*β*-arrestin complex, resulting in cAMP degradation (IV), down-modulation of inhibitory signals, and facilitates full T cell activation (B).

*PDE4*. Early studies showed PDE4 to be the predominant isoenzyme in the cytosolic fraction of human lymphocytes (Epstein and Hachisu, 1984) with *PDE4A*, *4B*, and *4D*, but not *4C*, contributing, presumably, to the overall hydrolytic activity (Giembycz, 1996; Gantner et al, 1997c; Jiang et al, 1998; Peter et al, 2007). PDE4 has long been known to play a key role in regulating T cell activation and functions (Michie et al, 1996; Sommer et al, 1997; Gantner et al, 1997b; Gantner et al, 1997c; Ekholm et al, 1997; Erdogan and Houslay, 1997; Barnette et al, 1998; Jin et al, 1998; Michie et al, 1998; Bielekova et al, 2000; Hatzelmann and Schudt, 2001; Kanda and Watanabe, 2001; Arp et al, 2003; Claveau et al, 2004; Asirvatham et al, 2004; Abrahamsen et al, 2004; Jimenez et al, 2004; Bjorgo and Tasken, 2006; Peter et al, 2007; Jin et al, 2010). A key mechanism appears to be the modulation of signal transduction through the TCR by signaling through PGE_2_ via EP2 and EP4 receptors, adenosine via A2a and A2b receptors that yield cAMP (Fig. 17). Activation of the TCR leads to cAMP production localized in lipid rafts, activation of PKA and, subsequently, the inhibition of the TCR signal through a PKa-Csk inhibitory pathway scaffolded by Ezrin-EBP50-PAG/Cbp anchoring complex (Abrahamsen et al, 2004; Bjorgo and Tasken, 2006; Wehbi and Tasken, 2016). However, engagement of the co-stimulatory receptor, CD28, leads to the recruitment of *β*-arrestin and PDE4 to lipid rafts and a decrease in the local cAMP pool and PKA activity (Fig. 18). PDE4 inhibitors downregulate the TCR signal by increasing the local cAMP concentration and PKA activity, which counteract the CD28-induced recruitment of PDE4. Thus, localized activities of cAMP, PKA, and PDE4 regulate the upstream TCR signal necessary for T cell activation and the subsequent initiation of effector functions (Schafer et al, 2014; Wehbi and Tasken, 2016). As for downstream effects of cAMP signaling, inhibition of PDE4 in CD4^+^ T cells by broad-spectrum and selective inhibitors leads to suppression of effector functions, including cell proliferation in response to TCR signals and costimulation, as well as cytokine production and cell motility (Ekholm et al, 1997; Sommer et al, 1997; Gantner et al, 1997c; Pette et al, 1999; Bielekova et al, 2000; Hatzelmann and Schudt, 2001; Jimenez et al, 2001). In a series of in vivo experiments using gene knockout mice, it was shown that cytokine production by Th2 cells is dependent on PDE4B expression, whereas Th1 cells were apparently unaffected (Jin et al, 2010).

*PDE7*. PDE7, primarily PDE7A, is also expressed in human lymphocytes, albeit to a lesser extent than PDE3 and PDE4; PDE7A1 is primarily cytosolic, whereas PDE7A2 mainly associates with a particulate fraction (Bloom and Beavo, 1996; Giembycz, 1996; Bender and Beavo, 2006). Subsequent studies showed that PDE1B, PDE7A, and PDE8A were induced following the activation of human lymphocytes (Jiang et al, 1998; Glavas et al, 2001; Kanda and Watanabe, 2001). Moreover, a critical requirement of PDE7A induction for full T cell activation has been reported (Li et al, 1999; Guo et al, 2009). Although the potential of PDE7 as a therapeutic target to treat inflammation has been investigated in several laboratories, it still remains controversial (Szczypka, 2020; Zorn and Baillie, 2023). In this respect, an antisense oligonucleotides approach has implicated PDE7A in T lymphocyte activation (Li et al, 1999). In contrast, T cells from Pde7a-deficient mice were activated normally by anti-CD3/CD28 (Yang et al, 2003). Similarly, PDE7 inhibitors did not impair CD3/CD28-dependent activation of human CD4^+^ T-lymphocytes (Nueda et al, 2006). It has been suggested that PDE7 may be a target for treating inflammation in conjunction with inhibition of PDE4. Thus, the PDE7 inhibitor, BRL 50481, enhanced the inhibitory effect of rolipram on lymphocyte proliferation and cytokine release (Smith et al, 2004). Additionally, T-2585, a potent PDE4 inhibitor (IC_50_ = 0.013 nM), which also inhibits PDE7 with an IC_50_ = 1.7 μM, inhibited proliferation and cytokine release from T cells under conditions in which the highly selective PDE4 inhibitor, piclamilast, had no effect (Nakata et al, 2002). Similarly, the PDE inhibitor, ASB16165, which inhibits PDE7A with an IC_50_ = 15 nM and PDE4 with an IC_50_ = 2.1 μM, also inhibited anti-CD3/CD28-stimulated T cell proliferation and cytokine release (Kadoshima-Yamaoka et al, 2009c). Additionally, in an in vivo mouse model of smoke-induced lung inflammation, combined antisense inhibition of the expression of PDEs 4B, 4D, and 7A produced a much greater anti-inflammatory effect than the use of the PDE4-selective inhibitor, roflumilast, and alone (Fortin et al, 2009). Given that inhibition of PDE7 can often enhance the effects of a PDE4 inhibitor, medicinal chemistry efforts were initiated to produce compounds that can selectively and potently inhibit both of the cAMP PDEs, although, at the time of writing, a definitive role for PDE7 as a target for mitigating inflammation remains to be established (Huang et al, 2023).

*PDE8*. Few PDEs are currently the targets of FDA-approved drugs, and there is a significant knowledge gap about the potential therapeutic role of other PDE isoforms particularly for the treatment of inflammatory diseases. The cAMP-specific PDEs, PDE8A, and PDE8B, have been the subject of numerous studies (Fisher et al, 1998; Hayashi et al, 1998; Soderling et al, 1998; Glavas et al, 2001; Kobayashi et al, 2003; Dong et al, 2006; Chen et al, 2009b; Dong et al, 2010; DeNinno et al, 2011; Tsai and Beavo, 2012; Maurice, 2013; Brown et al, 2013; Shimizu-Albergine et al, 2016; Vang et al, 2016; Johnstone et al, 2017; Basole et al, 2017; Kelly, 2018b; Dong et al, 2015). PDE8A and PDE8B are expressed widely across human tissues (Wang et al, 2008a) and have been implicated in testosterone and corticosteroid production (Tsai et al, 2010; Demirbas et al, 2013), myocyte contraction (Patrucco et al, 2010), lymphocyte adhesion and chemotaxis (Dong et al, 2006; Vang et al, 2010; Vang et al, 2013; Dong et al, 2015; Basole et al, 2017), memory and coordination (Tsai et al, 2012), human airway smooth muscle relaxation (Johnstone et al, 2017), immune protection against intracellular pathogens (Blanco et al, 2017), brain disorders associated with inflammation (Chimienti et al, 2019), and systemic lupus erythematosus (Orlowski et al, 2008). In addition, a recent study identified SNPs in the *PDE8B* locus that were associated with susceptibility to Sjögren’s Syndrome (Taylor et al, 2017).

T cell activation induces PDE8A1 (Glavas et al, 2001), a splice variant that has an affinity for cAMP that is up to 100 times higher than PDE4 isoforms (Fisher et al, 1998; Soderling et al, 1998; Hayashi et al, 1998; Gamanuma et al, 2003; Bender and Beavo, 2006). This property of the PDE8 family suggests that they may regulate changes in baseline cAMP gradients around cell signaling complexes. The availability of PDE8 inhibitors and disruptors has greatly enhanced the ability of scientists to interrogate the function of PDE8 in vitro and in vivo. It has been shown that PDE8A regulates the motility of lymphocytes and breast cancer cells, including adhesion to endothelial cells under physiological shear stress and chemotaxis (Dong et al, 2006; Vang et al, 2010; Vang et al, 2013; Dong et al, 2015; Basole et al, 2017). These functional effects seem to be uniquely controlled by PDE8 and are distinct from PDE4-regulated outcomes (Vang et al, 2016). The therapeutic activity of biologicals and compounds interacting with molecular targets on pathogenic T cells has been demonstrated in vitro and in vivo (Yednock et al, 1992; Brocke et al, 1999; Steinman, 2005; Healy and Antel, 2016). Current observations reveal PDE8 to be one of those targets for blocking T_eff_ cell motility and, potentially, inflammation (Dong et al, 2006; Vang et al, 2010; Dong et al, 2015; Vang et al, 2013; Vang et al, 2016; Basole et al, 2017). The possible role of PDE8 as an anti-inflammatory target has been examined in vivo. Thus, in experimental autoimmune encephalomyelitis (EAE) induced by immunization with a myelin oligodendrocyte glycoprotein peptide, a model of multiple sclerosis, the PDE8 inhibitor, PF 04957325, suppressed clinical signs of EAE, inflammatory lesion formation and accumulation of Th1 and Th17 effector T cells in the CNS (Brocke et al, 1999; Basole et al, 2022). Collectively, these data demonstrate the efficacy of pharmacologically targeting PDE8 as a treatment of autoimmune inflammation by reducing the inflammatory lesion load.

*PDE9*. PDE9A1 and a novel splice variant, PDE9A5, have been detected in human T cells (Wang et al, 2003b). PDE9A5 was localized to the cytosolic, whereas PDE9A1 was expressed exclusively in the nucleus. The function of PDE9 in T cells and whether it has a regulatory role in controlling inflammation is unknown.

*Regulatory T Cells*. It is well established that T-effector (T_eff_) cells and T_reg_ cells express high and relatively low levels of PDEs, respectively. The low abundance of PDEs in T_reg_ cells and high level of cAMP have been linked to the mechanism by which this T-cell subset suppresses the function of T_eff_ cells through the direct cell-to-cell transfer of cAMP (Bopp et al, 2007; Bopp et al, 2009; Vaeth et al, 2011). Mechanistically, the transcription factor, forkhead box P3 (Foxp3), expressed in T_reg_ cells has been shown to selectively repress genes, including those encoding PDEs, leading to elevated levels of intracellular cAMP (Bopp et al, 2007; Bopp et al, 2009; Vaeth et al, 2011). Remarkably, T_reg_ cell subsets in mice show significantly lower expressions of *Pde1a*, *Pde1b*, *Pde2a*, *Pde3b*, *Pde4b*, *Pde5a*, *Pde7a*, and *Pde8a* compared with naive T_eff_ cell subsets (Vang et al, 2013). Consistent with these findings, Foxp3 represses *Pde3b* and reducing *Pde3*b expression by genetic means permits normal T_reg_ cell homeostasis and T_reg_ cell-specific gene expression (Gavin et al, 2007). In contrast, T_reg_ and T_eff_ cells express comparable levels of *Pde4b3*, *Pde4d*, and *Pde7a* (Vang et al, 2013). It has also been reported that microRNA-mediated repression of *Pde3b* critically regulates peripheral immune tolerance (Anandagoda et al, 2019). Although the regulation of selected PDE isoforms including *Pde8* through Foxp3 in T_reg_ cells is well established, the exact role of PDE isoforms regulating T_reg_ cell function remains to be elucidated.

*B cells*. Studies conducted in the 1990s revealed that *PDE3A*, *PDE4A*, *PDE4B*, *PDE4D,* and *PDE7A* were the predominant isoenzymes in human-isolated CD4^+^ and CD8^+^ T lymphocytes (Tenor et al, 1995a; Giembycz, 1996). Human-isolated CD19^+^ B lymphocytes express a similar complement of PDE mRNA transcripts, but in contrast to T cells, PDE3 activity is marginal. Indeed, *PDE3B* was absent by PCR analysis, and only a weak signal for *PDE3A* mRNA was detected. No evidence for PDE1, PDE2, and PDE5 activity was found in these purified B cells (Gantner et al, 1998). It has been reported that the expression of *PDE7B* mRNA and protein in B-chronic lymphocytic leukemia (B-CLL) cells is 23-fold higher than in normal human B cells, and the most abundant PDE transcript expressed (Zhang et al, 2008b). Inasmuch as PDE7 inhibitors induce apoptosis of B-CLL cells, it has been suggested that PDE7B may be a therapeutic target for the treatment of CLL (Zhang et al, 2008b). The role of PDE7B as a therapeutic target for treating inflammation has not been reported.

## III Chapter 3: General perspectives and future directions

Over the last 50+ years (1972–date), it has become clear that cyclic nucleotide PDEs represent a large superfamily of enzymes that can be exploited to therapeutic advantage with selective inhibitors (Fig. 1). Many diseases of the internal organs and the immune system appear to be associated with aberrant cyclic nucleotide signaling or can be effectively treated with interventions that elevate either cAMP or cGMP. This has resulted in the approval by the FDA of 31 PDE inhibitors for the treatment of a variety of conditions that relate to cardiovascular, respiratory, and male sexual health. PDE inhibitors have also found their way to dermatological application, which has been reviewed elsewhere (Napolitano et al, 2018; Milakovic and Gooderham, 2021; Sidbury et al, 2023). Other inhibitors are undergoing preclinical evaluation or have entered clinical trials for the treatment of cancers, fibrosis, and several neurological disorders. Today, our extensive knowledge of the diversity and properties of the 11 PDE families suggests that therapeutic utility could be improved by developing allosteric PDE inhibitors or interventions that modify protein/protein interactions at specific PDE signalosomes of interest. Resonating throughout this entire review is the awareness that the specificity of nanodomain-level measurements and PDE subtype or isoform inhibition requires improvement. In addition, the development of specific PDE stimulators is an area of interest that needs to grow.

### A Toward drug specificity

PDE subtype-specific functions within the myocardial cell are a typical example of the importance of the development of more specific inhibitors and more precise measurement of nanodomain processes. The cellular mechanisms controlled by each PDE family or subtype unmistakenly point to the need to target specific PDE isoforms in circumscribed microdomains of myocardial cells, rather than using a pan-PDE approach to produce global changes in cyclic nucleotide concentrations. IN the background of this knowledge, the measurement of global tissue, circulating or excreted cyclic nucleotide concentrations seems to lose its meaningfulness. Even PDE isoform-selective modulators can be ineffective when the isoform is expressed in different cell types or compartments, particularly when those compartments serve opposing functions, for instance when it concerns the relocation of a PDE subtype or isoform as a consequence of the pathophysiological status of the cell (see sections Modulation of Cardiac Function by PDEs and Smooth Muscle Cells, Endothelial Cells, and PDEs). The biology of PDE1A, B, and C in smooth muscle cells can serve as a typical example.

On this ground, the search for more selective ways to inhibit and activate specific isoforms and spatially restricted pools of PDEs will continue. The lessons learned during the rapid evolution of PROTACS from proof-of-concept to clinical trials herald a new opportunity for PDE4 modulation (Li and Crews, 2022). Differential targeting of PROTACS to a subset of proteins with similar structures, or a subpopulation of a single target, can be achieved by leveraging small divergencies in protein conformations, oligomerization, cellular location, and activation state (Tao et al, 2022a). These advances are all germane to PDEs, whose structure between members of the same family can be similar and the cellular location of individual “pools” of single PDE isoforms influence the function of that “pool.” The event-driven pharmacology of PROTACs is also a good fit for PDE modulation as targeted degradation occurs at lower concentrations than conventional pharmaceuticals and is much less likely to induce side effects due to less reliance on high target occupancy (Li and Crews, 2022). The synergy between what is known about PDEs and PROTAC development has started to build and may be an important aspect of PDE pharmaceuticals in the future.

Independent of the chemical identity of the drug, beneficial effects might stem from isoform-specific PDE inhibition or activation (for example, by modifying PDE expression, targeting GAF domains or regulatory domains or by post-translational modifications) as well as alteration of PDE location (Baillie et al, 2019). The development of compartment-specific, PDE-isoenzyme-selective modulators (eg, by using cell-permeable peptide disrupters to displace the PDE isozyme from its signaling complex; Blair and Baillie, 2019) could help achieve this goal. In addition, the complex crosstalk between cAMP and cGMP intracellular signaling, and the presence of various PDE isoforms in different compartments, could require multidrug therapy to fine-tune the cyclic nucleotide level in specific subcellular compartments. For example, activating PDE4D near RYR2 or PDE4B or PDE2A near LTCCs while, at the same time, inhibiting PDE3A or PDE5 near phospholamban, could restore normal compartmentalization of cAMP and cGMP during hypertrophy and prevent the transition toward HF.

### B Targeting specific driver mutations

An alternative approach to enhancing PDE specificity involves the concept of driver mutations, particularly relevant in oncology. Having that said, one of the most important incentives to define driver mutations in cancer is that they are powerful predictors of response to therapeutic agents that target that mutation or its associated cellular actions. For example, somatic mutations in codon V600 of the *BRAF* proto-oncogene predict responsiveness to BRAF inhibitors in numerous human cancers, and mutations in each of *EGFR*, *ALK*, and *ROS1* in lung cancer predict responsiveness to inhibitors specifically targeting the protein tyrosine kinases encoded by each of these genes. The impressive therapeutic responses to these drugs in these mutationally defined cancers provide strong support for the role of the associated driver mutations in pathobiology of these cancers. This concept provides impetus for the development of drugs targeting the *PDE8B* and/or *PDE11A* driver mutations in adrenal, testis, and other cAMP-signaling cancers. Because these are loss-of-function mutations, such therapeutics would need to activate residual PDE activity in these cancers, potentially by increasing expression of the non-mutated (wild-type) *PDE8B* or *PDE11A* allele present in the germline state, by augmenting the functions of other PDE families in these cells, or by lowering cAMP levels by other means, thereby compensating for the loss of PDE8 or PDE11 action. Although there are no commercially available drugs that augment the enzymatic action of these PDEs, there is a tool compound that allosterically stimulates PDE11A4 activity (Jager et al, 2012).

**Fig. 19:**
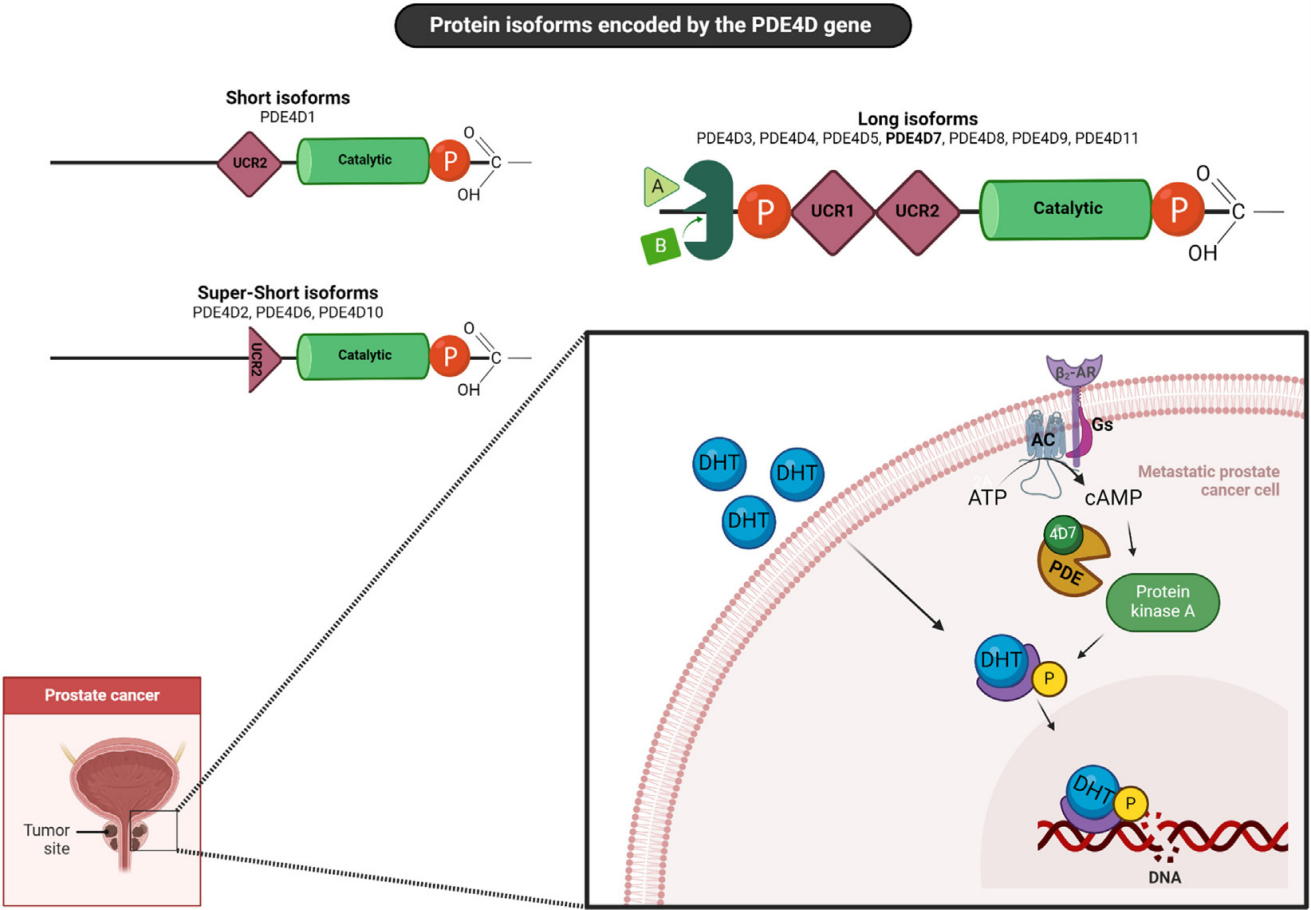
mRNA and protein isoforms encoded by the human *PDE4D* gene. The *PDE4D* gene products are divided into “long” isoforms that contain UCR1, UCR2 and the catalytic region; “short” isoforms that lack UCR1, and “super-short” isoforms lacking UCR1 and a portion of UCR2. Also, the C-terminal region (COOH), common to all PDE4D isoforms but not present in PDE4A, PDE4B, or PDE4C isoforms. Dihydrotestosterone (DHT) act at the androgen receptor which is activated by phosphorylation by protein kinase A. PKA is further regulated by cAMP signaling pathway. Created with BioRender.com.

Further attempts to develop allosteric modulators for therapeutic implicate PDE4 (Baillie et al, 2019; Omar et al, 2019). One area of interest is in targeting PDE4D7 in prostate cancer (Fig. 19; Gretarsdottir et al, 2003; Wang et al, 2003a). Prostate cancer is characterized by its responsiveness to steroid hormones, such as dihydrotestosterone (Huggins and Hodges, 1941). These hormones are agonists at the androgen receptor, which is a ligand-dependent transcription factor that regulates the expression of numerous genes. The androgen receptor is phosphorylated and, thereby, activated by several kinases, including PKA (Nazareth and Weigel, 1996; Cox et al, 2000; Kvissel et al, 2007; Waldkirch et al, 2010; Merkle and Hoffmann, 2011; Desiniotis et al, 2010; Sarwar et al, 2014; Moen et al, 2017; Dagar et al, 2019). There is abundant evidence for active, adenylyl cyclase-cAMP signaling in prostate cancer cells. These include those driven by the *β*_2_-adrenoceptor and receptors for vasointestinal peptide and pituitary adenylate cyclase-activating peptide (Gutierrez-Canas et al, 2003; Zhang et al, 2011a; Flacke et al, 2013). Numerous PDE isoforms have been detected in normal and neoplastic prostate tissue (Uckert et al, 2001; Uckert et al, 2006) and prostatic smooth muscle cells (Kedia et al, 2012). However, in contrast to the extensive and growing list of pharmacologically targetable mutations in many human cancers, there has been no documentation to date of mutations in cAMP signaling in prostate cancer. Early reports of somatic mutations or SNPs in *PDE4B*, *PDE6C*, *PDE7B,* and *PDE10A* in prostate cancer (de Alexandre et al, 2015) have not been reproduced by other groups (Quigley et al, 2018; Wedge et al, 2018; Abida et al, 2019), and their significance as driver mutations has yet to be demonstrated. However, a number of studies have shown PDE4D7 is downregulated in late-stage, aggressive, androgen-independent prostate cancer (Henderson et al, 2014) and that loss of PDE4D7 promotes androgen-independence and alterations in DNA transcription, replication, and repair (Gulliver et al, 2023). Alterations in PDE4D7 mRNA and/or protein abundance in prostate cancer patient specimens may have prognostic value (Bottcher et al, 2015; Bottcher et al, 2016; Alves de Inda et al, 2018; van Strijp et al, 2018; van Strijp et al, 2019), and they have been proposed to represent novel biomarkers to help classify the risk of disease progression (Henderson et al, 2019; Gulliver et al, 2022). These observations have provided the impetus to develop therapies that specifically target PDE4D7 in prostate and possibly other cancers. Because major portions of PDE4D7 are also seen in other “long” PDE4D isoforms, including the regulatory/dimerization UCR1 and UCR2 domains, as well as the catalytic site (Fig. 19), such therapies would probably not target these regions of the protein but, instead, be directed to its unique *N*-terminus.

### C Conclusion

The importance of prioritizing PDE selectivity to enhance clinical effectiveness, while concurrently amplifying selectivity to minimize adverse drug reactions, constitutes a pivotal aspect in the developmental trajectory of PDEs. This narrative traces back to the inception of PDE research, marked by the serendipitous discovery of the beneficial effects of caffeine, epitomized by asthmatic Henry Hyde Slater’s experience in 1886. The field has expanded dramatically over the last 4 decades and moved on to form a vast, multidisciplinary research landscape that occupies a growing part of the pharmacopeia. The versatility and nanodomain functions of PDEs make one aware of the intricacy of cellular signaling, and of the possibility that hitherto we might only have scratched the surface of all the potential the research field offers for the improvement of pharmacotherapy.

The research also forces scientists to look over the edges of current technical possibilities and move to the magnification of increasingly smaller details, leading to the growth of drug development possibilities, not only through allosteric binding of PDEs but also through the modulation of binding partners or perhaps even the correction of gene expression that is affected by driver mutations. The increased importance of bringing more detail in the research also entails the question of what is the proper model for each isoform, per cell type, and even nanodomain. Are cell culture experiments representative or are even rodents or larger animals apt to predict which PDE is a drug target in humans? What about the emerging field of organoid research? These considerations will shape the success of drug discovery. It is expected that the list of possible clinical indications and compounds will be growing in the coming decades. In other words: PDE research will remain an exciting field to follow, from molecular biology all the way to the clinic.

## Conflict of interest

Stefan Brocke is a member of the Scientific Advisory Board of MindImmune, Inc. David A. Kass is on the advisory board of Cardurion Pharmaceuticals that is testing PDE9 inhibitors in humans with heart failure. Gary A. Piazza and Adam B. Keeton are co-founders of ADT Pharmaceuticals, Inc. and consultants. Gretchen Snyder is a full-time employee of Intra-Cellular Therapies, Inc. and holds equity in the company. George S. Baillie is founder and scientific advisor of Disruptyx Therapeutics. Christian Hesslinger and Peter Nickolaus are employees of Boehringer Ingelheim Pharma GmbH & Co. KG, 88397 Biberach an der Riss, Germany. Anton J.M. Roks receives funding from Health-Holland/Erasmus MC/Intracellular Therapies TKI grant # EMCLSH23035 for research on PDE1 in vascular aging. All other authors declare no conflicts of interest.
